# The Genome of *Anopheles darlingi*, the main neotropical malaria vector

**DOI:** 10.1093/nar/gkt484

**Published:** 2013-06-12

**Authors:** Osvaldo Marinotti, Gustavo C. Cerqueira, Luiz Gonzaga Paula de Almeida, Maria Inês Tiraboschi Ferro, Elgion Lucio da Silva Loreto, Arnaldo Zaha, Santuza M. R. Teixeira, Adam R. Wespiser, Alexandre Almeida e Silva, Aline Daiane Schlindwein, Ana Carolina Landim Pacheco, Artur Luiz da Costa da Silva, Brenton R. Graveley, Brian P. Walenz, Bruna de Araujo Lima, Carlos Alexandre Gomes Ribeiro, Carlos Gustavo Nunes-Silva, Carlos Roberto de Carvalho, Célia Maria de Almeida Soares, Claudia Beatriz Afonso de Menezes, Cleverson Matiolli, Daniel Caffrey, Demetrius Antonio M. Araújo, Diana Magalhães de Oliveira, Douglas Golenbock, Edmundo Carlos Grisard, Fabiana Fantinatti-Garboggini, Fabíola Marques de Carvalho, Fernando Gomes Barcellos, Francisco Prosdocimi, Gemma May, Gilson Martins de Azevedo Junior, Giselle Moura Guimarães, Gustavo Henrique Goldman, Itácio Q. M. Padilha, Jacqueline da Silva Batista, Jesus Aparecido Ferro, José M. C. Ribeiro, Juliana Lopes Rangel Fietto, Karina Maia Dabbas, Louise Cerdeira, Lucymara Fassarella Agnez-Lima, Marcelo Brocchi, Marcos Oliveira de Carvalho, Marcus de Melo Teixeira, Maria de Mascena Diniz Maia, Maria Helena S. Goldman, Maria Paula Cruz Schneider, Maria Sueli Soares Felipe, Mariangela Hungria, Marisa Fabiana Nicolás, Maristela Pereira, Martín Alejandro Montes, Maurício E. Cantão, Michel Vincentz, Miriam Silva Rafael, Neal Silverman, Patrícia Hermes Stoco, Rangel Celso Souza, Renato Vicentini, Ricardo Tostes Gazzinelli, Rogério de Oliveira Neves, Rosane Silva, Spartaco Astolfi-Filho, Talles Eduardo Ferreira Maciel, Turán P. Ürményi, Wanderli Pedro Tadei, Erney Plessmann Camargo, Ana Tereza Ribeiro de Vasconcelos

**Affiliations:** ^1^Department of Molecular Biology and Biochemistry, University of California Irvine, Irvine, CA 92697, USA, ^2^Institute of Technology, Broad Institute of Harvard and Massachusetts, Cambridge, MA 02141, USA, ^3^Laboratório de Bioinformática do Laboratório Nacional de Computação Científica, Petrópolis, RJ 25651-075, Brasil, ^4^Departamento de Tecnologia, Faculdade de Ciências Agrárias e Veterinárias de Jaboticabal, UNESP -Universidade Estadual Paulista, SP 14884-900, Brasil, ^5^Departamento de Biologia, Universidade Federal de Santa Maria, Santa Maria, RS 97105-900, Brasil, ^6^Departamento de Biologia Molecular e Biotecnologia, Centro de Biotecnologia, Universidade Federal do Rio Grande do Sul, Porto Alegre, RS 91501-970, Brasil, ^7^Departamento de Bioquímica e Imunologia, Universidade Federal de Minas Gerais, Belo Horizonte, MG 31270901, Brasil, ^8^Department of Medicine, University of Massachusetts Medical School, Worcester, MA 01655, USA, ^9^Laboratório de Entomologia Médica IPEPATRO/FIOCRUZ, Porto Velho, RO 76812-245, Brasil, ^10^Departamento de Microbiologia, Imunologia e Parasitologia, Universidade Federal de Santa Catarina, Florianópolis, SC 88040-900, Brasil, ^11^Centro de Ciências da Saúde, Universidade Estadual do Ceará, Fortaleza, CE 62042-280, Brasil, ^12^Departamento de Ciências Biológicas, Campus Senador Helvídio Nunes de Barros, Universidade Federal do Piauí, Picos, PI 60740-000, Brasil, ^13^Departamento de Genética, Instituto de Ciências Biológicas, Universidade Federal do Pará, Belém, PA 66075-900, Brasil, ^14^Department of Genetics and Developmental Biology, University of Connecticut Health Center, Farmington, CT 06030, USA, ^15^Informatics, The J. Craig Venter Institute, Medical Center Drive, Rockville, MD 20850, USA, ^16^Departamento de Genética, Evolução e Bioagentes, Instituto de Biologia, Universidade Estadual de Campinas, Campinas, SP 13083-862, Brasil, ^17^Departamento de Genética e Melhoramento, Universidade Federal de Viçosa, MG 36570-000, Brasil, ^18^Centro de Apoio Multidisciplinar, Universidade Federal do Amazonas, Manaus, AM 69077-000, Brasil, ^19^Departamento Biologia Geral, Universidade Federal de Viçosa, Viçosa, MG 36571-000, Brasil, ^20^Laboratório de Biologia Molecular, Instituto de Ciências Biológicas, Universidade Federal de Goiás, Goiânia, GO 74001-970, Brasil, ^21^Divisão de Recursos Microbianos, Centro Pluridisciplinar de Pesquisas Químicas, Biológicas e Agrícolas, Universidade Estadual de Campinas, Paulinia, SP 13140-000, Brasil, ^22^Centro de Biologia Molecular e Engenharia Genética, Universidade Estadual de Campinas, Campinas, SP 13083-875, Brasil, ^23^Departamento de Biotecnologia, Universidade Federal da Paraíba, João Pessoa, PB 58051-900, Brasil, ^24^Departamento de Biologia Geral, Universidade Estadual de Londrina, Londrina, PR 86055-990, Brasil, ^25^Instituto de Bioquímica Médica, Universidade Federal do Rio de Janeiro, Rio de Janeiro, RJ 21941-902, Brasil, ^26^Programa de Pós-graduação em Genética, Conservação e Biologia Evolutiva, Instituto Nacional de Pesquisas da Amazônia, Manaus, AM 69067-375, Brasil, ^27^Laboratório Nacional de Ciência e Tecnologia do Bioetanol – CTBE, Campinas, São Paulo, SP 13083-970, Brasil, ^28^Departamento de Ciências Farmacêuticas, Faculdade de Ciências Farmacêuticas de Ribeirão Preto, Universidade de São Paulo, Ribeirão Preto, SP 14040-903, Brasil, ^29^Coordenação de Biodiversidade, Laboratório Temático de Biologia Molecular, Instituto Nacional de Pesquisas da Amazônia, Manaus, AM 69060-001, Brasil, ^30^Laboratory of Malaria and Vector Research, NIAID, NIH, Bethesda, MD 20817, USA, ^31^Departamento de Bioquímica e Biologia Molecular, Universidade Federal de Viçosa, Viçosa, MG 36570-000, Brasil, ^32^Departamento de Biologia Celular e Genética, Universidade Federal do Rio Grande do Norte, Natal, RN 59072-970, Brasil, ^33^Programa de Pós-Graduação de Genética e Biologia Molecular, Instituto de Biociências, Universidade Federal do Rio Grande do Sul, Porto Alegre, RS 90150-197, Brasil, ^34^Instituto de Ciências Biológicas, Departamento de Biologia Celular, Universidade de Brasília, Brasília, DF 70910-900, Brasil, ^35^Departamento de Biologia, Universidade Federal Rural de Pernambuco, Recife, PE 50740-520, Brasil, ^36^Departamento de Biologia, Faculdade de Filosofia, Ciências e Letras de Ribeirão Preto, Universidade de São Paulo, Ribeirão Preto, SP 14040-901, Brasil, ^37^Instituto de Ciências Biológicas, Universidade Federal do Pará, Belém, PA 66075-970, Brasil, ^38^Programa de Pós-Graduação em Ciências Genômicas e Biotecnologia, Universidade Católica de Brasília, Brasília, DF 70790-160, Brasil, ^39^Empresa Brasileira de Pesquisa Agropecuária Soja, Londrina, PR 86001-970, Brasil, ^40^Empresa Brasileira de Pesquisa Agropecuária, CNPSA, Concórdia, SC 89700-000, Brasil, ^41^Departamento de Biologia Vegetal, Universidade Estadual de Campinas, Campinas, SP 13083-970, Brasil, ^42^Laboratório de Vetores da Malária e Dengue, Instituto Nacional de Pesquisa da Amazônia, Manaus, AM 69067-375, Brasil, ^43^Laboratório de Bioinformática e Biologia de Sistemas, Universidade Estadual de Campinas, Campinas, SP 13083-875, Brasil, ^44^Departamento de Bioquímica e Imunologia, Universidade Federal de Minas Gerais, Belo Horizonte, MG 31270-901, Brasil, ^45^Instituto de Biofísica Carlos Chagas Filho, Universidade Federal do Rio de Janeiro, Rio de Janeiro, RJ 21941-902, Brasil and ^46^Departamento de Parasitologia, Instituto de Ciências Biomédicas, Universidade de São Paulo, São Paulo, SP 05508-000, Brasil

## Abstract

*Anopheles darlingi* is the principal neotropical malaria vector, responsible for more than a million cases of malaria per year on the American continent. *Anopheles darlingi* diverged from the African and Asian malaria vectors ∼100 million years ago (mya) and successfully adapted to the New World environment. Here we present an annotated reference *A. darlingi* genome, sequenced from a wild population of males and females collected in the Brazilian Amazon. A total of 10 481 predicted protein-coding genes were annotated, 72% of which have their closest counterpart in *Anopheles gambiae* and 21% have highest similarity with other mosquito species. In spite of a long period of divergent evolution, conserved gene synteny was observed between *A. darlingi* and *A. gambiae*. More than 10 million single nucleotide polymorphisms and short indels with potential use as genetic markers were identified. Transposable elements correspond to 2.3% of the *A. darlingi* genome. Genes associated with hematophagy, immunity and insecticide resistance, directly involved in vector–human and vector–parasite interactions, were identified and discussed. This study represents the first effort to sequence the genome of a neotropical malaria vector, and opens a new window through which we can contemplate the evolutionary history of anopheline mosquitoes. It also provides valuable information that may lead to novel strategies to reduce malaria transmission on the South American continent. The *A. darlingi* genome is accessible at www.labinfo.lncc.br/index.php/anopheles-darlingi.

## INTRODUCTION

*Anopheles darlingi* is the principal neotropical malaria vector (1–6), sustaining the transmission of more than a million malaria cases per year on the American continent [(7), World Health Organization Malaria Report 2011]. *Anopheles darlingi* has a wide geographic distribution that reaches from Southern Mexico to Northern Argentina and from East of the Andes chain to the coast of the Atlantic Ocean. Although this species has been subjected to extensive study, little is known about the molecular aspects of its biology. The *A. darlingi* genome presented here fills this gap in the knowledge about its genes, transcripts and proteins that determine the biological characteristics of this important malaria vector.

**Figure 1.**
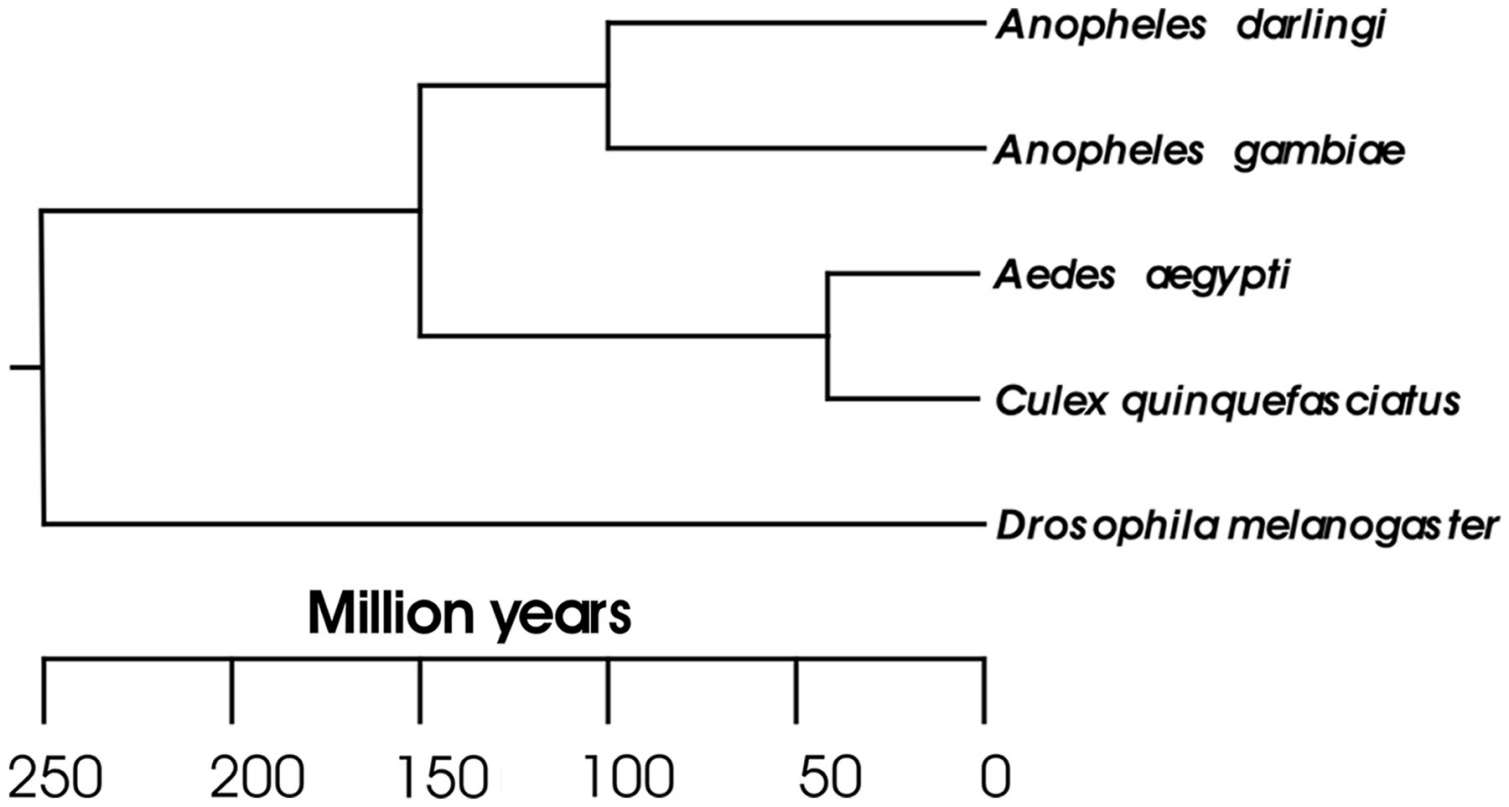
Phylogenetic relationships of five dipteran species (adapted from [11]). The evolution relationship and divergence time of *A. darlingi* in comparison with species of the *Anopheles*, *Aedes*, *Culex* and *Drosophila* genera.

In spite of the availability of published genomes for three other mosquito species [*Anopheles gambiae* (8), *Aedes aegypti* (9), *Culex quinquefasciatus* (10)], the medical and epidemiological significance of *A. darlingi* and its phylogenetic position support the importance of this study. *Anopheles (Nyssorhynchus*) *darlingi* and *A.* (*Cellia*) *gambiae* are considered to have diverged ∼100 mya (11) (Figure 1), suggesting that their most recent common ancestor lived before the geological split of western Gondwana (∼95 mya). This estimation is supported by the absence of the *Cellia* species in the New World and *Nyssorhynchus* in the Afro-Eurasian continents. The most ancient human colonization of the American continent is still a matter of discussion and is estimated to have occurred 30 000–10 000 years ago (12–16), indicating that *A. darlingi* and its ancestral species evolved in an environment devoid of humans or human ancestral species for several million years. Furthermore, European colonialists transferred *Plasmodium falciparum* and *Plasmodium vivax*, the most prevalent malaria parasites, to the American continent in post-Colombian times (17,18). Therefore, interactions between neotropical malaria vectors and humans, and malaria parasites, are relatively recent. The evolutionary history of *A. darlingi* thus allows tackling basic and unanswered questions about vector–parasite and vector–human host interactions as well as about malaria parasite development within its vectors and the mosquito immune responses to the developing parasite.

## MATERIALS AND METHODS

### Genome

Gravid *A. darlingi* female mosquitoes were captured from Coari, Amazonas State, Brazil, and their progeny (F1) was reared at the insectary of the Laboratory of Malaria and Dengue Vectors, Instituto Nacional de Pesquisas da Amazônia, Manaus, Brazil. Larvae were fed powdered fish food (Tetramin®), and pupae were transferred to plastic cups filled with distilled water. Total DNA was extracted from 1884 recently emerged adults (F1, <24 h after emergence), males and females, and was used for sequencing. High-coverage whole-genome data sets were generated by 454 Life Sciences (Roche) technology using single fragment end and paired-end reads. The reads were assembled using Celera Assembler 6.1. Because the sequenced DNA was sampled from a large number of field-captured individuals, the assembly was performed with a relaxed error tolerance of 16%, except during unitig construction where it was 12%. K-mer size overlap generation was also relaxed to 16 bases.

### Transcriptome

The transcriptome of adult *A. darlingi* was derived from two mosquito populations that were captured 524 km apart from each other (Coari, Amazonas State and Porto Velho, Rondonia State, Brazil). The extracted RNA was sequenced using two next-generation sequencing platforms: 454 Life Sciences (Roche) and Illumina (Solexa sequencing). Transcripts were reconstructed using mapping first strategy, Genomic Short-read Nucleotide Alignment Program and Scripture and the assembly first strategy, Velvet/Oases. Reconstructed transcripts were used as supporting evidence on the annotation of the genome (PASA - Program to Assemble Spliced Alignments). Additional details on genomic DNA and RNA extraction, sample preparation, sequencing, assembly and annotation are given in Supplementary Method SA.

## RESULTS AND DISCUSSION

### Genome size, genome and transcriptome sequencing, assembly and annotation

Five and a half billion base pairs of information were generated, resulting in an assembled *A. darlingi* genome that spans 173.9 Mb (Tables 1 and 2) (see Supplementary Tables SA1 and SA2). The size of the *A. darlingi* haploid genome was determined by cytometric analysis to be ∼201 Mb (2C = 0.41 pg) (see Supplementary Method SB and Supplementary Figure SB1), which is ∼30% smaller than the genome of *A. gambiae* [278 Mb, (8)] and three to six times smaller than the genome of culicinae mosquitoes *C. quinquefasciatus* [579 Mb, (10)] and *A. aegypti* [1379 Mb, (9)] but larger than the *Drosophila melanogaster* genome [176 Mb, (19)]. The difference between the cytometrically determined genome size and the sum of all of the contigs and scaffolds is most likely the result of unassembled centromeres, telomeres and other portions of the genome that are rich in repetitive DNA sequences. In fact, 18,66 percent of the reads were not included in the final assembly. Assuming a uniform coverage of 20× and a read average length of 248 bp, the unassembled reads correspond to 32.71 Mb, which accounts for an estimated total genome length of 206.6 Mb, a value that is similar to the value obtained by cytometry. Although the *A. darlingi* genome is smaller than that of *A. gambiae*, the sums of the lengths of all of the protein coding sequences in each of the two genomes are similar (18.2 and 19.3 Mb, respectively), which indicates a more compact genome in *A. darlingi* mosquitoes (see Supplementary Tables SA3 and SA4). *Anopheles darlingi* has shorter intergenic and intronic sequences and fewer transposable elements (TEs; these elements constitute only 2.3% of the genome; see details below). Nevertheless, *A. darlingi* genes display a larger average number of exons per gene (4.6) than *A. gambiae* (4.4) (see Supplementary Table SA5).

**Table 1.** Assembly statistics of *A. darlingi* reference genome

Feature	Statistics
Total number of good sequence reads	16 777 488
Sequence reads in assembly	14 139 351
Total number of scaffolds	8233
Total length of scaffolds	173 918 288
Total number of contigs	13 857
Combined bases in contigs	172 639 290
Combined length of gaps	1 278 998
Sequencing coverage	20
N50 scaffold length	81 222
N50 contig length	37 754
Longest scaffold (number of contigs)	1 087 588 (10)
Shortest scaffold (number of contigs)	473 (1)

**Table 2.** General characteristics of the *A. darlingi* genome

Genome feature	*A. darlingi*	*A. gambiae*^a^	*A. aegypti*^a^
Genome size (Mb)	173.92	278.25	1379
Percent of G + C (%)	48.15	40.9	38.2
Protein coding length (Mb) and (% genome length)	18.2 (10.4)	64.92 (23.3)	224.9 (16.3)
Total number of exons	47 990^b^	56 210	66 827
Number of protein-coding genes	10 457^b^	12 670	15 704
Percent genes with introns (%)	91.59	93.6	90.1
Average number of exons per gene	4.6	4.4	4.0
Average gene length (bp)	1735	5124^c^	14 587^c^
Total tRNAs	346	450	995

^a^Statistics were derived from genome updates for *A. gambiae* AgamP3 (Vectorbase, version 66.3) and *A. aegypti* AaegL1 (Vectorbase, version 66.1).

^b^Includes 13 mitochondrial genes.

^c^Includes introns but not untranslated regions.

DNA sequences of bacterial origin were obtained along with the *A. darlingi* genome. For example, the complete genome of *Aeromonas hydrophila* was assembled during an initial analysis of the 454 reads. DNA sequences of bacterial origin were labeled as contaminants and were screened out during the assembly process. Even after applying the bacterial DNA filter, the assembled *A. darlingi* genome includes genes of apparent bacterial origin. The majority of these are present in small contigs (mostly <10 kb) that do not contain evident mosquito DNA, which suggests that they derive from environmental contaminations or additional microorganisms that are associated with *A. darlingi*. Some scaffolds apparently contain sequences of both prokaryotes and eukaryotes. Further analyses are necessary to determine the legitimacy of these assembled scaffolds and the possibility of horizontal gene transfer events that may have contributed to shaping the *A. darlingi* genome.

Two similar mitochondrial genomes were previously described for this species, corresponding to the Southern and Northern genotypes, which originated from Manaus, Brazil and Central Cayo District, Belize, respectively. The typical 37 genes in animal mtDNA, comprising 13 protein-encoding genes, two rRNA genes (12S rRNA and 16S rRNA), 22 tRNA genes and a control region, are found in the complete *A. darlingi* mitochondrial genome (11). Here, we describe a third mitochondrial genome for this species, from mosquitoes captured in Coari, Brazil, which is more similar to the Southern genotype (see Supplementary Data SC and Supplementary Figure SC1). For the first time, we report the complete *A. darlingi* nuclear ribosomal RNA cistron (AD11084), complementing previously published, partial rRNA sequences (20,21). Sets of 359 nuclear encoded tRNAs and 44 homologs of *A. gambiae* pre-microRNAs (miRNAs) were identified. miRNA precursor candidates conserved in the genomes of *A. darlingi* and *A. gambiae*, which might play important roles in the posttranscriptional regulation of gene expression in these species, were described in a separate publication (22).

### Synteny

**Figure 2.**
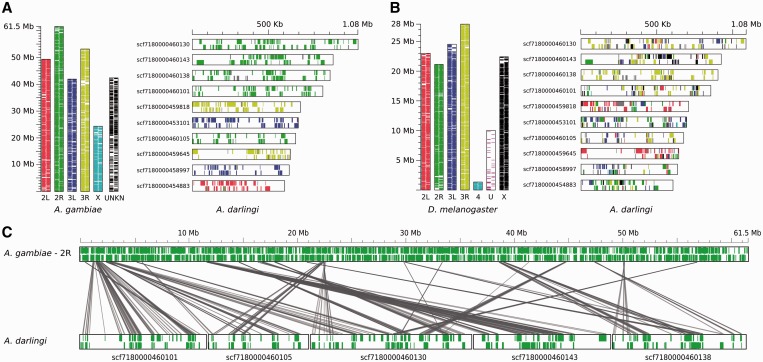
Comparison of gene organization between *A. darlingi*, *A. gambiae* and *D. melanogaster*. (**A**) Gene distribution along *A. gambiae* chromosomes and the location of their respective orthologs on the 12 largest *A. darlingi* scaffolds. Black-edged vertical and horizontal bars represent *A. gambiae* and *A. darlingi* chromosomes and scaffolds. Colored lines within each bar indicate the location and strand of genes: the leftmost or uppermost column indicates the plus strand; the rightmost or bottommost column indicates the minus strand. The color of those genes denotes either the chromosome where *A. gambiae* genes are encoded or, in the case of lines representing *A. darlingi* genes, the *A. gambiae* chromosome where their respective orthologs are encoded. Gray colored lines represent either *A. darlingi* genes without orthologs in *A. gambiae* or genes with two or more homologs in distinct *A. gambiae* chromosomes. (**B**) Gene distribution along *D. melanogaster* chromosomes and the 12 largest *A. darlingi* scaffolds. The results are presented in a schema equivalent to the one on panel A. (**C**) Distribution of *A. darlingi* orthologous genes along *A. gambiae* chromosome 2R. The five scaffolds with the longest alignment against chromosome 2R are depicted here. Each row contains black-edged horizontal bars representing either chromosomes (*A. gambiae)* or genomic scaffolds (*A. darlingi*). The green lines indicate the position and strand of the genes. The gray projections connect orthologous genes across organisms. Some of *A. darlingi* scaffolds had their orientation modified to facilitate the visualization of syntenic blocks.

In spite of ∼100 million years of evolutionary divergence between *A. darlingi* and *A. gambiae*, the gene synteny between their genomes is relatively well conserved. Translocation events have occurred but were mostly restricted to large intra-chromosomal rearrangements (Figure 2). The synteny between *A. darlingi* and *D. melanogaster* presents a different scenario: each one of the 12 largest *A. darlingi* scaffolds have orthologous genes scattered through different *D. melanogaster* chromosomes, which suggests a low degree of synteny (Figure 2B).

**Figure 3.**
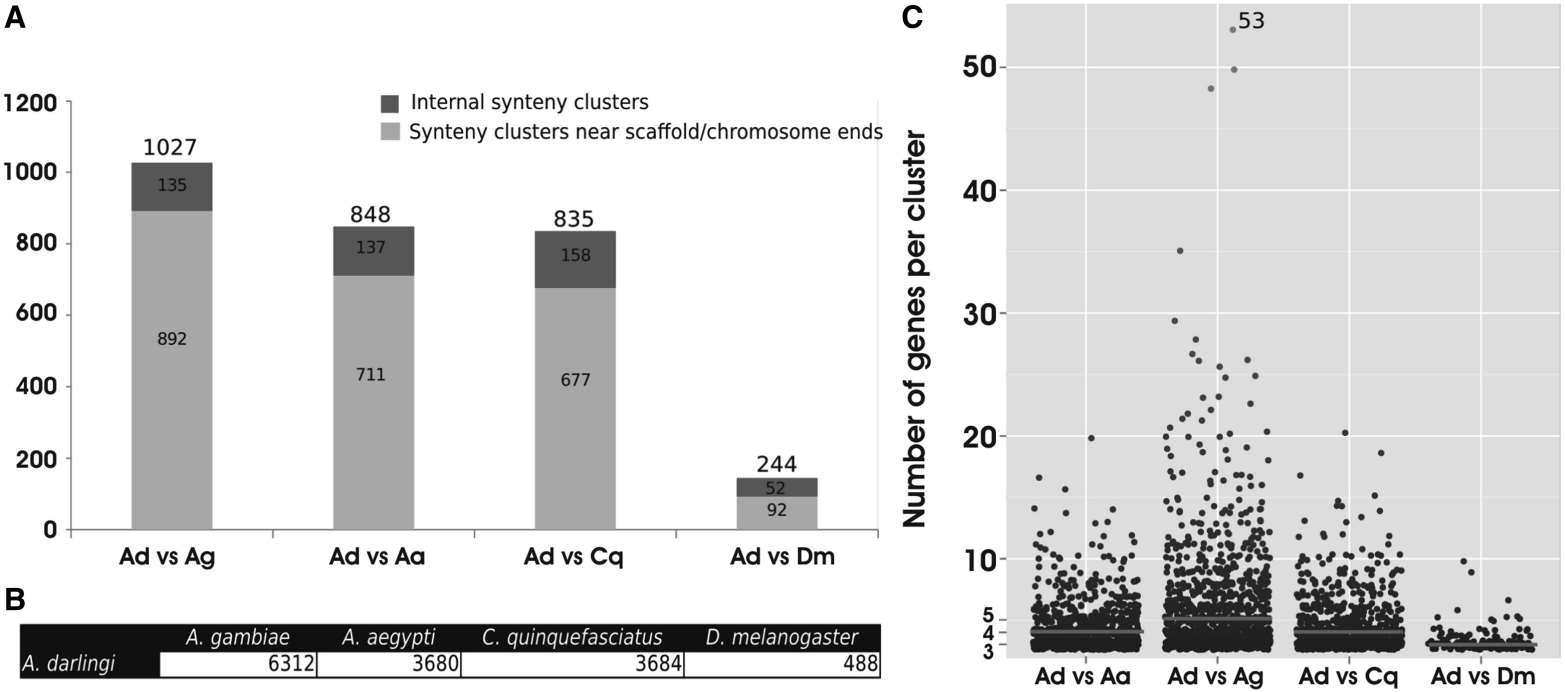
Synteny clusters statistics. (**A**) Distribution of the number of genes per synteny cluster when considering *A. darlingi* (Ad) versus either *A. gambiae* (Ag), *A. aegypti* (Aa), *C. quinquefasciatus* (Cq) or *D. melanogaster* (Dm). Data points represent synteny clusters with more than three protein-coding genes on each pairwise comparison. The points were scattered in each column for the purpose of facilitating visualization. Red horizontal lines indicate the media values of the distribution. (**B**) The total number of syntenic genes between each pair of species. (**C**) Number of synteny clusters identified on each comparison. The whole extent of the bars indicates the total number of clusters that were identified in each analysis, which was further divided into clusters located internally on scaffolds or chromosomes versus those near chromosomes or scaffold ends. Species names were abbreviated, as in panel A.

Systematic synteny evaluation between *A. darlingi* and *A. gambiae* identified 1027 synteny clusters (Figure 3A), comprising 6312 syntenic genes or ∼60% of all *A. darlingi* protein-coding genes (Figure 2B). Apart from giving an idea on how much large-scale rearrangements have been important in the divergence of these species, this analysis will help in future efforts for gene identification on the basis of conserved synteny. Similar analyses between *A. darlingi* and other dipterans, i.e. *A. aegypti*, *C. quinquefasciatus* and *D. melanogaster*, identified 848, 835 and 244 synteny clusters (Figure 3A) and 3680, 3684 and 488 syntenic genes (Figure 3B), respectively. The higher degree of synteny between *A. darlingi* and *A. aegypti* or *C. quinquefasciatus* in comparison with the values obtained by *A. darlingi*–*D. melanogaster* evaluation reflects the estimated divergence time among those species (Figure 1) and suggests that most of the interchromosomal rearrangements have taken place after the split of lineages that lead to Drosophilidae and Culicidae.

The median number of genes per synteny cluster was not significantly different among all of the pairwise synteny evaluations (Figure 3C). This observation is owing to the draft nature of the *A. darlingi* genome, which has a significant number of unclosed genome gaps; these gaps lead to premature ends of the synteny clusters. From all of the identified synteny clusters between *A. darlingi* and *A. gambiae*, 87% occur near scaffold ends (Figure 3), suggesting that those clusters will be extended further when the genome sequence gaps are mended. A detailed *A. darlingi* cytogenetic map has been described (23–28) (see Supplementary Figure SD1). It is expected that mapping of particular genes or clones on chromosomes, together with the described syntheny clusters, will support a more complete and precise assembly of the *A. darlingi* genome.

### Polymorphism within and between two populations

A database with >10 million single-nucleotide variants (SNVs) and short indels with potential use as genetic markers was created (Table 3) (see Supplementary Method SA). Differently from most of the previous studies of sequence polymorphisms in mosquitoes, that analyzed individuals pooled from established colonies in which much of the natural diversity is lost, the *A. darlingi* data presented here was generated from wild caught mosquitoes. The sequencing of the 278 Mb of the *A. gambiae* genome revealed ∼445 thousand single-nucleotide polymorphisms (SNPs), with an average heterozygozity at the nucleotide level of 1.6 per kb (9). The average frequency of nucleotide variation was reported to be 7 and 12 SNPs per kb for *Anopheles funestus* and A.aegypti (29,30), respectively. An SNP frequency of ∼17 per kb was recently reported for selected gene fragments of field-captured *Anopheles arabiensis* (31).

**Table 3.** Number and density of SNVs per genomic feature

Genomic feature	Genome (454)^a^	Transcriptome (454 + Illumina)^b^
Gene	1 643 685 (39.7 per kb)	819 427 (19.8 per kb)
Exon	488 652 (26.2 per kb)	494 539 (26.6 per kb)
Intron	1 155 083 (50.7 per kb)	324 926 (14.2 per kb)
CDS	475 903 (26.1 per kb)	481 588 (26.37 per kb)
Intergenic	6 811 677 (50.0 per kb)	835 447 (6.1 per kb)
Promoter	360 607 (41.8 per kb)	153 431 (17.8 per kb)

^a^Genome data from mosquitoes collected in Coari.

^b^Transcriptome data from Porto Velho and Coari were combined. Samples were sequenced by either 454 Life Science (454) or Illumina technologies.

Promoter = 2 kb upstream from transcript 5′-end.

Because laboratory autonomous colonies of *A. darlingi* are not available, the DNA and RNA sequenced in this project were extracted from >1884 individuals (F1 progeny of field-captured gravid females). While the high degree of polymorphism found in *A. darlingi* reads posed a challenge for genome assembly, the data acquired permitted a better representation of the sequence polymorphisms in two natural populations of this malaria vector. The distribution of SNVs is not homogeneous throughout the genome, and average values as high as 50 SNVs per kb in intergenic and intronic sequences were observed, with lower values in protein coding genes, including untranslated regions (UTRs) (40 SNVs per kb), and even lower values (26 SNVs per kb) in protein coding DNA sequences (CDSs). A total of 792 472 SNVs were uniquely found in the Coari data set, while 654 619 were identified only in the samples collected in Porto Velho. The SNVs identified in this study, though requiring validation, serve as the basis for high-throughput genotyping analysis and future population genetic and association mapping efforts.

### Transposable elements

TEs correspond to 2.3% of the *A. darlingi* genome (Table 4) (see Supplementary Data and Method SE and SF). The set of Class I and II TEs superfamilies is as diverse in *A. darlingi* as in the genomes of other mosquitoes; however, the number of TE copies is smaller in *A. darlingi*. In *A. gambiae*, TEs encompass 17% of the genome (9), and among the genomes of the *Drosophila* species so far analyzed, TE compositions vary from 2.7 to 23% (32).

**Table 4.** Transposable contents in mosquito genomes

TE class—Order	*A. gambiae*		*A. darlingi*		*A. aegypti*	
	Copy number	% of genome	Copy number	% of genome	Copy number	% of genome
Class I—LTR	4348	6.2	241	0.19	28 905	10.51
Class I—Non LTR	392	1.07	200	0.9	61 938	14.37
Class I—SINEs	2389	3.77	4610	0.51	101 838	1.88
Class II—DNA transposons	835	1.1	395	0.02	12 930	3.04
Class II—Helitrons	5	0.2	19	0.02	244	1.04
Class II—MITEs	3399	5.07	6635	0.66	419 955	15.8
Total	11 368	17.41	12 119	2.29	625 810	46.64

TEs were classified as proposed by (33): Class I retrotransposon, with LTR, retroposons without LTR or SINEs (short interspersed nuclear elements).

Class II were classified as DNA transposons, helitrons and MITES (miniature inverted-repeat TEs). *A. gambiae* and *A. aegypti* data from (8,9,34).

Some of the TEs found in the *A. darlingi* genome showed multiple identical copies and intact transposase Open reading frames (ORFs), suggesting that they are active elements. Among the putatively active TEs are the following: gypsy-like from long terminal repeats (LTR) elements order; jockey-like, Chicken repeat 1 (CR1) and retrotransposable element (RTE) families from non-LTR order; and mariner-like and Helitrons from DNA class II elements (see Supplementary Data SE and Supplementary Figures SE1 and Supplementary Table SF). Multiple applications of active TEs have been contemplated for advancing the understanding of mosquito biology as well as for genetic-based vector control strategies. Active TEs can be used in genetic engineering as transformation vectors and can be used for gene and enhancer trapping; they also can be used for genome-wide insertional mutagenesis studies (33).

### Protein coding genes

**Figure 4.**
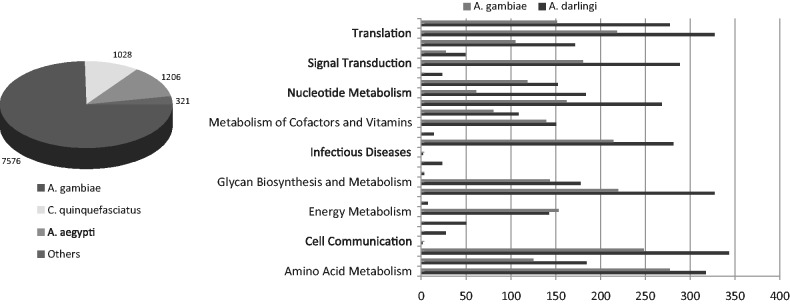
Distribution and functional categories of protein-coding genes predicted in Anopheles species. The best matches distribution of all (10 481) of the *A. darlingi* predicted protein coding genes in the KEGG database, by organisms; and the comparison of the molecular functions of the products of the predicted protein coding genes between *A. darlingi* and *A. gambiae*.

A total of 10 481 protein-coding genes were predicted in the *A. darlingi* genome. For checking the completeness of the *A. darling*i gene set, the core eukaryotic gene-mapping approach (CEGMA) (34) that assess genome completeness and gene structure prediction was applied. CEGMA analysis includes a set of core genes that are supposed to be highly conserved and single-copy genes present in all eukaryotes. The integral sequences of 235 out of 248 highly conserved eukaryotic genes (94.76%) were identified in the *A. darlingi* genome. Other eight highly conserved genes were found as partial loci. Despite these results indicating the efficiency of the gene prediction tools used, additional *A. darling*i protein coding genes are expected to be identified as future sequencing and assembling efforts will close the present gaps between scaffolds and contigs. From the *A. darlingi* protein coding genes, 72.3% have the closest counterpart in the *A. gambiae* genome and 21.3% have a gene that has the highest similarity within the genomes of other mosquitoes (*A. aegypti* or *C. quinquefasciatus*) (Figure 4) (see Supplementary Data SG). A comparative analysis of the functional categories of the genes comprising the *A. darlingi* and *A. gambiae* genomes showed that, in general, functional categories were equally represented (Figure 4). Genes associated with hematophagy (encoding components of mosquito olfaction and saliva), immunity and insecticide resistance are directly involved in vector-human and vector-parasite interactions and efforts to curb malaria transmission. Some of these genes, identified in the *A. darlingi* genome, will be further discussed.

### Olfaction

The chemosensory system plays essential roles in food source or host location, mate choice, predator avoidance, oviposition site selection and toxic-compound avoidance (35). Molecular components of insect chemosensory systems include at least three different types of chemosensory receptors: the odorant (OR), the gustatory (GR) and the ionotropic (IR) receptors (36). Two other types of proteins, i.e. the odorant-binding proteins (OBPs) and chemosensory proteins (CSPs), are involved in the perireceptor events of the chemosensory system (36,37).

#### Odorant receptors

In *A. gambiae*, a family of 79 putative odorant receptor (AgOR) genes have been identified (38,39), including AgamGPRor7, now named Agam\Orco (40), and the ortholog of *D. melanogaster* DmelOr83b, which serves as a coreceptor in all OR multimeric complexes (41). In the *A. darlingi* genome, we have identified 18 genes that encode putative ORs, including a gene encoding Adar\Orco (GPROR7) (see Supplementary Table SH1). It appears that the number of OR paralogs is reduced in *A. darlingi*. OR3, which in *A. gambiae* is part of a group of 25 paralogs, is represented in *A. darlingi* by seven paralogous genes; OR33 is represented by six paralogs in *A. gambiae* and by four in *A. darlingi*. Six ORs (OR8, OR23, OR34, OR39, OR42 and OR58) are represented by single genes in *A. darlingi*. OR23 and OR42 are, respectively, represented by 15 and 14 paralogs in *A. gambiae*.

#### Gustatory receptors

Sixty-one genes encoding putative GR have been identified in the *A. gambiae* genome. In the *A. darlingi* genome, 17 GR genes were identified (see Supplementary Table SH1), three of them (AD01104, AD08863 and AD09819) as partial sequences. Among them, four genes (AD07140/GPRGR14, AD08836/GPRGR15, AD08857/GPRGR17 and AD08840/GPRGR20) encode receptors that were described as candidate sugar receptors in *A. gambiae* (42). The proteins encoded by the genes AD09007, AD01029 and AD09985 correspond to the receptors GPRGR22, GPRGR23 and GPRGR24, respectively, and show a high conservation (71–93%) when compared with homologous sequences in *A. gambiae*, *A. aegypti* and *C. quinquefasciatus*. The corresponding orthologs of GPRGR22 and GPRGR24 in *D. melanogaster* (DmGr21a and DmGr63A) function as a heterodimeric receptor for carbon dioxide (43,44).

#### Variant ionotropic glutamate receptors

These receptors function as chemosensory receptors in *D. melanogaster* (45) and *A. gambiae* (46,47). In *A. gambiae*, a family of 46 variant ionotropic glutamate receptors was identified (47). In *A. darlingi*, we found 14 sequences related to variant ionotropic glutamate receptors (see Supplementary Table SH1).

#### Odorant binding proteins

A total of 69 genes encoding OBP were described in *A. gambiae*; many of them possibly originated from recent events of gene duplications. We have found 33 OBP encoding genes (see Supplementary Table SH1) in the present *A. darlingi* genome assembly. The reduced number of OBP genes suggests that duplication events were not as frequent in this species. Alternatively, the missing genes may be located in unassembled portions of the genome. In fact, besides the OBP genes annotated, TBLASTN searches identified sequences that likely correspond to truncated OBP-like genes. Sequences with similarity to 10 *A. gambiae* OBPs could not be identified in any of the *A. darlingi* contigs.

The genes AD02966 (OBP34), AD00512 (OBP37), AD01405 (OBP44) and AD01406 form part of a group of paralogs that in *A. gambiae* is composed of 16 genes. However, in other cases, the number of related sequences is similar in both species, i.e. AD04156 (OBP10), AD03416 (OBP18), AD07879 (OBP21), AD07746 (OBP25), AD03881 (OBP26), AD03880 (OBP28), AD06986 (OBP23) and AD03882 (AGAP012322), which in *A. gambiae* is also represented by eight sequences. The amino acid sequences of OBP34 (AD02966) and OBP37 (AD00512) are highly similar, with only three amino acid changes. In *A. gambiae*, OBP 34 and 37 present identical amino acid sequences (48).

#### Chemosensory proteins

Belonging to a class of soluble proteins that are found in the sensillum lymph of insect antennae, CSP exhibit binding activity toward odorants (49). CSP encoding genes have been identified in several insects, and among the mosquitoes, 21 genes were described in *C. quinquefasciatus* (50) and 8 in *A. gambiae* (51). Six of the CSP genes (AgamCSP1 to AgamCSP6) described in *A. gambiae* are part of a group of paralogs. In the *A. darlingi* genome, we identified four CSP genes (see Supplementary Table SH1), and all presented similarity to representatives of this paralogous group.

### Salivary proteins

The salivary gland (SG) is the only organ of *A. darlingi* that has been submitted to a tissue-specific transcriptome analysis (52,53). A total of 2371 clones from an adult female *A. darlingi* SG cDNA library were sequenced and assembled, allowing the identification of 183 protein sequences, 114 of which code for putatively secreted salivary proteins. A comparative analysis of SG transcriptomes of *A. darlingi* and *A. gambiae* reveals a significant divergence of salivary proteins. On average, salivary proteins are only 53% identical, while housekeeping proteins are 86% identical between the two species. *A. darlingi* proteins were found that match culicine but not anopheline proteins, indicating a loss or rapid evolution of these proteins in the old world *Cellia* subgenus. Additionally, several well-represented salivary protein families in old-world anophelines are not expressed in *A. darlingi*.

### Circadian rhythm

Rhythmic cycles of *Anopheles* mosquitoes command biting activity, mating swarms, nocturnal flight activity and egg laying; however, little work has been performed to elucidate the molecular basis for these daily rhythms (54). Throughout its geographical distribution, *A. darlingi* exhibits distinct patterns of biting behavior. One, two or three daily peaks of biting activity have been observed in different studied sites (55–58). The molecular basis for these differences in behavior is unknown. Here, we describe the *A. darlingi* circadian cycle-associated genes *timeless*, *cycle*, *clock*, *timeout* and *period* (see Supplementary Method and Data SI and Supplementary Figures SI1 and SI2). The identification of these genes will permit assessment of their expression levels and rhythmicity among the diverse *A. darlingi* populations.

### Insecticide resistance

Resistance to insecticides is a major threat to sustained reductions in malaria vector populations and malaria incidence. To date, there has been only a single report of insecticide resistance in natural *A. darlingi* populations. A population from Colombia was found to be resistant to both dichlorodiphenyltrichloroethane (DDT) and lambda-cyhalothrin (59). However, a number of studies reporting insecticide resistance in the African malaria vector *A. gambiae* as well as other vector mosquitoes should caution against complacency (60–64). The changing pattern of land use in the Amazonian region, resulting in increased urbanization and agricultural initiatives, and the associated escalation in insecticide use are expected to strengthen selection for insecticide resistance in *A. darlingi*.

#### Metabolic detoxification

Three gene families that are primarily involved in insecticide metabolism have been described: the cytochrome P450s (P450s), the carboxy/cholinesterases (CCEs) and the glutathione-S-transferases (GSTs) (65). Metabolic resistance is usually a result of overexpression or allelic variation in members of detoxifying enzyme families. We identified 89 P450s, 20 CCEs and 30 GSTs genes in *A. darlingi* (see Supplementary Table SJ1). GSTs are the most conserved among the three superfamilies (66), and this conservation permitted the identification of putative orthologs between *A. darlingi* and *A. gambiae* that had a sequence identity that was >70%. Four classes of cytosolic GSTs were identified: the most conserved theta (five genes), zeta (one gene), the insect-specific delta (three genes) and epsilon (six genes) classes. Only members from the Delta and Epsilon classes have been implicated in insecticide resistance. Among the epsilon members in *A. darlingi*, GSTe2 (AdGSTe2, AD08205) is highly conserved among culicines (*A. gambiae*, *A. aegypti* and *C. quinquefasciatus*) and metabolizes DDT in *A. gambiae* and *A. aegypti* (67,68). Several AdGST genes remained unclassified, with no obvious orthologs in the *A. gambiae* genome, and thus, they might represent novel GSTs.

The CCEs and P450s appear to have undergone a slight expansion in *A. gambiae* in comparison with *A. darlingi*. It is possible, considering the redundancy in these families, that different family members are co-opted for functions in insecticide resistance in different mosquito populations, such as P450s and some GSTs that have increased mRNA accumulation in some, but not all, *A. gambiae* insecticide-resistant populations (60–64). Additionally, genes encoding a superoxide dismutase (AY745234) and a peroxiredoxin (XP_308081.2) also presented increased mRNA accumulation in these populations.

#### Target-site insensitivity

Decreased target site sensitivity to pyrethroids and DDT in *A. gambiae* has been described as being associated with two alternative substitutions at a single codon in the sodium channel gene (L1014F or L1014S) and is referred to as knockdown resistance, or kdr (69–72). A comparison of the voltage-gated sodium channel (VGSC) gene sequence across different insect species showed that it is highly conserved, but different numbers of exons are observed among species (73). In *A. gambiae*, 33 exons have been identified, which can synthesize different mRNAs through alternative splicing. Two putative VGSC genes were identified in the *A. darlingi* genome [AD07884 (2e-75; 98% identity) and AD00168 (3e-38; 45% identity)]. Primers based on the *A. gambiae* sodium channel sequence had previously failed to amplify the *A. darlingi* ortholog (59,69). The now available *A. darlingi* VGSC sequences permit the development of specific diagnostic tools for detecting kdr resistance in this species.

Target-site resistance to carbamates and, to a lesser extent, organophosphates (OP) in culicines result from a mutation in the acetylcholinesterase gene (*ace-1*). This gene is absent in *Drosophila*, possibly because of a secondary loss, and OP resistance in this organism arises from mutations in the *ace-2* gene, which is ubiquitous in insects. The putative *A. darling* ace-1 homolog is AD00377 (4e-38; 98% identity when compared with *Anopheles albimanus*) (74). In *A. gambiae*, a second copy of *ace-1* (*ace-1D*) has been described, and its high frequency and distribution in countries of West Africa points to an association with resistance (75). The availability of *A. darlingi ace-1*, VGSC and other detoxifying gene sequences allow the development of specific diagnostic tools for detecting incipient insecticide resistance in this species. This is especially important in epidemiological vigilance because evolutionary forces acting on *A. darlingi*, when facing continuous and increasing exposure to insecticides, could lead to widespread insecticide resistance.

### Immunity-related genes

The mosquito immune system plays a critical role in limiting the spread of malaria and other vector-borne diseases. We analyzed sequences related to the three major immune response systems in Dipterans, Toll, immune deficiency (IMD) and thioester proteins (TEPs) (see Supplementary Table SK1) because these genes and their associated signaling pathways are known to limit the spread of malaria parasites in anophelines. Identifying the *A. darlingi* orthologous genes relative to each component of the *D. melanogaster* and/or *A. gambiae* pathways is challenging, especially where multigenic families such as Toll receptors or the TEPs are involved. In contrast, one to one orthologs of most of the signaling molecules were more easily identified. Although the *A. darlingi* immune system appears to be organized similar to those of other Diptera, exact orthologs of many of the important receptors have not yet been established. The presently assigned putative homologous functions must be asserted by actual bench experiments to gain a full appreciation of *A. darlingi* immunity.

#### Toll pathway

We identified four *A. darlingi* genes that are related to the Toll ligand known as spätzle (SPZ), when six SPZs were found in both *A. gambiae* and *D. melanogaster* (see Supplementary Figure SK1) (76). Two of these genes are possible orthologs of the SPZ1 group, which include *Drosophila spätzle*, the ligand for Toll. The other two are orthologous to SPZ3 or SPZ6. *Drosophila melanogaster* has nine Tolls; only Toll and Toll7 have established immune functions, while the functions of the *A. gambiae* Tolls are still largely undefined. Clear orthologs to the fruit fly genes could not be identified for most of the seven *A. darlingi* Tolls that were identified, although a Toll7 ortholog was assigned. Conversely, 1:1 orthologs were found for nearly all of the known signaling molecules in the Toll pathway, including MyD88, Tube, Pelle, TRAF6 and the NF-κB/I-κB orthologs Rel1/Cactus.

#### Peptidoglycan recognition proteins and the Immune deficiency pathway

Eight peptidoglycan recognition proteins (PGRPs) were identified in the *A. darlingi* genome, three of which are likely to be catalytic type 2 amidases. PGRP-LC, a well-established receptor for DAP-type peptidoglycan and activation of the IMD pathway in fruit flies, appears to have two orthologs in *A. darlingi*. Additional orthologs of known PGRPs were identified, although only peptidoglycan recognition protein-LB 5′-untranslated region (PGRP-LB) has an established function, which is involved in degradation of PGN, a non-catalytic PGRP. Ten additional IMD pathway members (including the negative regulator CASPAR and essential signaling components such as IMD, (Fas-associated death domain containing protein - FADD and Death-related ced-3/Nedd2-like protein - DREDD) FADD and DREDD) were found on a 1:1 orthology basis.

#### Thioester proteins

TEPs play a role in Diptera that is similar to the role of complement in humans: they directly opsonize bacteria and parasites, which leads to death and melanization. Ten possible TEPs were identified in *A. darlingi*. The *A. gambiae* TEP1 gene product has been proposed as a key regulator of malaria infection. A definite ortholog of TEP1 was not identified in *A. darlingi*, although several of the *A. darlingi* TEPs are in the subfamily in which TEP1 is included.

#### Antimicrobial peptides

*Drosophila melanogaster* has, at a minimum, seven families of antimicrobial peptides. Similar to other mosquito species, most of these antimicrobial peptides were not readily apparent in the *A. darlingi* genome. However, genes encoding two well-known classes of antimicrobial peptides that are found in the genome of other mosquitoes were identified in *A. darlingi*: one member of the Defensin family and three Cecropins.

## CONCLUSIONS

Malaria was once epidemic in most areas in Central and South America (7,77,78). Economic development and the associated environmental changes that have occurred during the 20th century have drastically reduced malaria transmission in subtropical areas. However, malaria is still a major public health problem in the Amazon basin, where >500 thousand malaria cases occur every year. Because *A. darlingi* is the main malaria vector in the Amazon, and also for its interesting phylogenetic position, the Brazilian National Council for Research included this species among those selected as priorities for having their genomes sequenced (79). Here, we present the *A. darlingi* genome as a valuable platform for basic and applied sciences.

Laboratory colonization of *A. darlingi* has proven to be difficult, and presently there are no available autonomous colonies of this species. Nonetheless, large numbers of wild *A. darlingi* mosquitoes are easily captured in the Amazon, and raising the progeny of captured gravid females has allowed the sequencing of the mosquitoes genome and transcriptome, which complements studies of *A. darlingi* biology, behavior, physiology, genetics, biochemistry and insecticide resistance (4,11,23,22,53,80–85). The successful colonization of other neotropical anopheline species (86,87) and older reports of *A. darlingi* that were successfully adapted to breed in laboratory conditions (88–90) indicate that colonizing *A. darlingi* is an attainable task. The availability of this genome will promote efforts to establish an autonomous viable free-mating laboratory *A. darlingi* colony.

As the first neotropical Anopheles species of the subgenus *Nyssorhynchus* with its genome sequenced and annotated, the data presented here open a new window from which we can contemplate the evolutionary history of these mosquitoes. Comparative evolutionary genomics is one of the most rapidly advancing disciplines in the biological sciences and offers the opportunity to study evolutionary changes among organisms, to identify genes that are conserved among species, and to study the genes that give each organism its own specific characteristics (91). Questions that are related to malaria vectorial capacity, anthropophily and hematophagy among anophelines can now be addressed from the perspectives of two distantly related members of the Anopheles genus that diverged ∼100 mya and evolved in two distinct environments (11). *Anopheles darlingi* orthologs of genes associated with insecticide resistance have been identified, allowing a more targeted examination of insecticide resistance status in populations of this vector species (60). A catalog of *A. darlingi* immunity-related genes will help in studies of vector–parasite interactions and will promote research to understand the determinants of vectorial capacity and competence (92). Finally, we identified 349 *A. darlingi* predicted genes that encode products with no hit in the Kyoto Encyclopedia of Genes and Genomes (KEGG) database (see Supplementary Table SL1), thus potentially related to adaptations to the New World environment. This study and other recently published and ongoing efforts to sequence the genomes and transcriptomes of malaria vectors (93,94) (vectorbase.org) will provide a needed and more complete understanding of malaria vector biology.

It is our hope that this report provides valuable information that will lead to novel strategies to reduce the rate of malaria transmission on the South American continent.

## ACCESSION NUMBERS

The sequence of *A. darlingi* has been deposited in the DDBJ/EMBL/GenBank database under the following accession number: ADMH00000000. The version described in this paper is the second version, ADMH02000000.

## SUPPLEMENTARY DATA

Supplementary Data are available at NAR Online: Supplementary Tables SA1-SA5, SF, SJ1, SK1, SH1, SL1, Supplementary Figures SB1, SC1, SD1, SE1, SI1, S12, SK1, Supplementary Methods SA, SB, SE, SI, Supplementary Data SC, SE, SG, SI and Supplementary References [96–104].

Supplementary Data
