# Supplementary material for: The Genome of Anopheles darlingi, the main neotropical malaria vector
Source: Nucleic Acids Res. 2013 Jun 12;41(15):7387–400. doi: 10.1093/nar/gkt484 (PMC3753621; doi:10.1093/nar/gkt484)
Supplement: Supplementary Data [file supp_41_15_7387__index.html]

The Genome of Anopheles darlingi, the main neotropical malaria vector — Supplementary Data 

# The Genome of *Anopheles darlingi*, the main neotropical malaria vector

## 

files

**Files in this Data Supplement:**

- Supplementary Data - zip file
