# Supplementary material for: The Genome of Anopheles darlingi, the main neotropical malaria vector
Source: Nucleic Acids Res. 2013 Jun 12;41(15):7387–400. doi: 10.1093/nar/gkt484 (PMC3753621; doi:10.1093/nar/gkt484)
Supplement: Supplementary Data [file supp_gkt484_nar-00257-h-2013-File006_updated.zip › S-E.docx]

**S-E – Transposable elements**

Transposable elements (TEs) are DNA sequences that can mobilize and increase their copy number in genomes. These elements are present in almost all organisms and are important for genome structure and evolution. Additionally, they have potential as drivers for transgene introduction in insect populations, as a means to control diseases that are spread by these vectors [102]. TEs are classified into two classes based on the transposition intermediary, RNA (class I) or DNA (class II). These classes are further subdivided into subclass, order, superfamily, family and subfamily, according to their sequence similarities and structural relationships [95].

The fraction of genomes that correspond to TEs is extremely variable. For example, 80% of the maize genome, 45% of the human genome and 10% of the Drosophila genome are formed by TEs [103]. In mosquitoes, for example, the difference between the genome sizes of *Ae. aegypti* (1.3 Gb) and *An. gambiae* (278 Mb) is substantially ascribed to TEs. In *An. gambiae*, TEs encompass 17% of the genome, whereas in *Ae. aegypti* they represent 47% of the genome size [8, 9,33]. In this report, we characterize the TE composition of the *An. darlingi* genome and compare it with the other available mosquito genomes.

**Annotation of Transposable Elements**

Two complementary approaches were used to annotate TEs. In the first, the contig sequences were screened for ORFs with the software *Getorf* from the EMBOSS package. The resulting ORFs were subsequently translated into amino acid residues by the *Transeq* software, also from the EMBOSS package. This translation allowed us to use the improved sensibility of the PSI-BLAST algorithm to search the genome using a library of annotated TE proteins. This TE data set was built employing a search in NCBI. Initially a library of 75,569 transposable element sequences was built from NCBI entrez queries using the following keywords “mobile genetic OR transposon* OR retroelement OR retro-element OR retrotransposon* OR retro-transposon* OR helitron* OR transposons OR helitrons OR retrotransposons OR retrotransposition* OR retro-transposons OR retroelements OR retro-elements OR helentron* OR helitron OR MITE* OR MITES OR SINE* OR SINES OR "reverse transcriptase" OR "Long interspersed" OR ALU”. To this dataset we incorporated the GIRINST transposon element databank and the *Anopheles gambiae* transposable element dataset determined in the AnoDB database. This final library contained 123,512 nucleotide sequences from a wide range of transposable elements.

After two filtering steps that were executed with the aim of discarding insignificant hits, a data set with 1496 highly significant hits that potentially represent individual transposable element sequences was produced. A total of 1,273 hits were identified the search using an e value lower than e-10 (the current default value for a minimal significant match). To the BLASTp search results we filtered out sequences with less than 40% of identity and alignment size shorter than 150 residues. A total of 1,273 hits were identified the search using an e value lower than e-10 (the current default value for a minimal significant match). To the BLASTp search results we filtered out sequences with less than 40% of identity and alignment size shorter than 150 residues. This final data set was then manually annotated to place each sequence into the corresponding transposable element class and family. A second approach was used to annotate MITEs and SINEs. These TEs were annotated through the *ab initio* MITE/SINE detection software MUST.

Transposable elements in the *An. darlingi* genome are represented by the main TEs superfamilies and are almost as diverse as in other mosquitoes. Among the RNA-mediated TEs (class I), the SINEs are the prevalent elements, and the *Ty3-gypsy* are also well represented (Fig. 1). Concerning the DNA-mediated TEs (class II), the non-autonomous MITEs are by far the most abundant, whereas among the autonomous elements, the *Helitrons* are the most profuse. Nevertheless, the copy number of annotated TEs in the *An. darlingi* genome is significantly lower than that observed in *An. gambiae*. For example, 4,348 copies of the LTR retrotransposon and 835 DNA transposons were annotated in *An. gambiae*, values that decrease, respectively, to only 441 and 414 copies in *An. darling*. The genome size difference observed between *An. gambiae* and *Ae. aegypti* has been attributed to a variation in the TEs number [10]. However, although *An. darlingi* and *An. gambiae* have genomes of similar size, TEs represent only 2.3% of the former genome but 17% of the latter. In the same way, the *Drosophila* genomes so far analyzed have shown differences in TE composition that vary from 2.7 to 23% [32].

Some of the TEs found in the *An. darlingi* genome showed intact ORFs and identical copies, suggesting that they are active elements. Active TEs can be used as transformation vectors as well as for gene and enhancer trapping, or genome-wide insertional mutagenesis studies [33]. Among these putatively active *An. darlingi’*s TEs, we found the following: *gypsy*-like from LTR elements’ order; *jockey*-like, *CR1* and *RTE* from non-LTR order; and *mariner*-like and *Helitrons* from DNA class II elements. Understanding the activity and regulation of TEs and their behavior in mosquito populations is important for achieving the success of such genetic approaches.

We have performed an analysis of similarity between the TEs encountered in the mosquito genomes (*An. darlingi*, *An. gambiae*, *Ae. aegypti* and *Cu. quinquefasciatus*) and do not find any evidence of horizontal gene transfer (HGT) events. HGT events between the *An. gambiae* and *Ae. aegypti* have been shown [9] and no evidence were found for this phenomenon. Our results also suggested that HGT is not common in mosquito genome evolution.


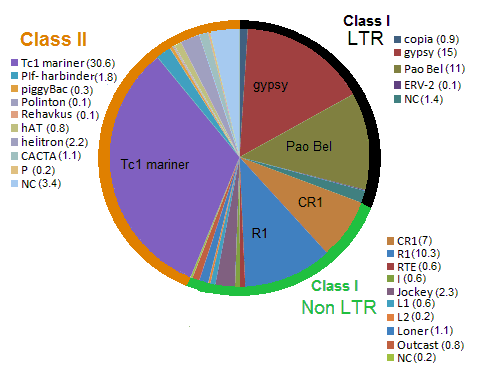


**Figure S-E1. Classes and superfamilies representation, in percentage, present in *An. darlingi* genome.** The external circle represents the classes or subclasses. The inner circle represents the superfamilies found. The numbers inside parenthesis are the fraction in % of each superfamily. The SINEs and MITEs are excluded due the elevated copy number.

**Excel worksheets (S-F)**.

**Worksheet 1** - Annotated elements in *An. darlingi* genome. Column A= Class and subclass; column B= superfamily; column C= scaffold number in that the element was found; column D= the best Blast hit in our TE databank; column E= % of identity with the Blast hit; F= Matches with the Blast hit; G= mismatches with the Blast hit; H = number of GAPs; I = position in the scaffold of start for the Blast hit; J= I = position in the scaffold of end for the Blast hit; K= position in the 3´end of TE sequence for Blast hit, L= position in the 5´end of TE sequence for Blast hit; M= e-value of Blast hit; N= score for Blast hit; O= size of annotated TE in bp.

**Worksheet 2 -** Summary of copy number (column B) and sums of sequence length for each class or sub class (Class I – LTR and non LTR; Class II) (column C).

**Worksheet 3 -** Summary for TE composition in *An. darlingi* genome in different superfamilies. Column A-Classes and subclasses; Column B–Superfamily; Column C–copy number; Column D – percentage of each family in the TE total.

Annotated TEs in *An. darlingi* genome. MITEs and SINES are not shown in this annotation due their elevated copy number.
