# Supplementary material for: The Genome of Anopheles darlingi, the main neotropical malaria vector
Source: Nucleic Acids Res. 2013 Jun 12;41(15):7387–400. doi: 10.1093/nar/gkt484 (PMC3753621; doi:10.1093/nar/gkt484)
Supplement: Supplementary Data [file supp_gkt484_nar-00257-h-2013-File006_updated.zip › S-G.docx]

**S-G Protein coding genes**

A number of proteins that are related to gene expression have been found in the *An. darlingi* genome: 27 genes for RNA polymerase subunits, 29 genes for basal transcription factors, 68 genes for snRNA binding proteins and splicing auxiliary factors, 65 genes for ribosomal proteins, 36 genes related to tRNA aminoacylation, 45 genes for ribosome biogenesis, 133 genes for translation factors, and 359 tRNA genes. We found 83 genes related to nucleo-cytoplasmic RNA transport, including 19 components of the nuclear pore complex and 15 components of pre-tRNA-, pre-rRNA- and pre-mRNA-specific transport pathways. In addition, 41 genes related to the mRNA surveillance pathways were found, including 9 components of the exon-junction complex, 7 components of the nonsense-mediated decay pathway and 2 components of the no-go decay pathway. Several transcription factor protein families are represented in the *An. darlingi* genome, and the majority is conserved among insect genomes. For example, basic helix-loop-helix, homeobox, RING, PHD Zn finger domain proteins are present in similar numbers of genes in *An. darlingi* and *An. gambiae*. On the other hand, POZ, RNP-1 RNA recognition motif, CCHC Zn finger and MYND Zn finger domain proteins appear to be underrepresented, while C2H2 domain proteins appear to be overrepresented, in the *An. darlingi* genome.

Interest in developmental gene regulation in *Anopheles* and other mosquitoes has been influenced by potential applications of the resulting knowledge. Specifically, the regulation of genes related to reproduction, the immune system, olfaction and blood feeding are examples of adaptive variations that make the Anopheles a successful group of insects. The *An. darlingi* genome contains at least 196 ORFs engaged in signaling pathways that have been predicted within the KEGG database. The components of these signaling pathways are conserved, and many of these signaling cascades are employed in a variety of developmental processes. The most represented is the mTor (mechanistic target of rapamycin) pathway, with 56 identified ORFs. Tor (target of rapamycin), Raptor (regulatory-associated protein of mTOR) and S6K (a serine/threonine-protein kinase) genes were identified. The last component acts downstream from the mTOR signaling in response to growth factors and nutrients, promoting cell and tissue proliferation, maintaining tissue homeostasis, and controlling responses to environmental stress and aging. The WNT pathway is composed of at least 45 ORFs. The Wnt/β-Catenin pathway regulates cell fate decisions during development in vertebrates and invertebrates. The genes for Frizzled receptor and the most major downstream components of the Wnt pathway were identified, such as Disheveled and B-catenin proteins. Similar to Wnt components, the Hedgehog pathway is highly conserved in mosquito species. The *An. darlingi* genome presents at least 15 ORFs of this pathway, including the Patched segmentation polarity protein, which acts as a receptor for the hedgehog protein (HH) and associates with the smoothened protein to transduce the signal leading to the activation of other genes and also Patched. The Notch signaling pathway, which contains at least 13 ORFs, is also evolutionarily conserved and regulates multiple cell differentiation processes during embryonic and adult life, among others. The Jak-STAT signaling pathway uses a combination of tyrosine kinases and Stat proteins in cytokine/growth factor signaling. More than 10 ORFs of this pathway were identified, such as the tyrosine-protein kinase Hopscotch, Stam (signal transducing adapter molecule) and the tyrosine protein phosphatase Corkscrew. The MAPK signaling pathway is also present with at least 12 ORFs, including the tyrosine protein kinase receptor Torso and several downstream genes, which are important for terminal patterning in developmental processes. The phosphatidylinositol signaling pathway for information processing was identified as being present with 27 ORFs; this pathway acts on phosphatidylinositol and is committed to producing the second messenger inositol-triphosphate. The TGF-beta (transforming growth factor beta) signaling pathway is involved in many cellular processes in both the adult organism and the developing embryo, including cell growth, cell differentiation, apoptosis and other cellular functions. The *An. darlingi* genome contains 18 ORFs that are related to this pathway, including the BMPR (bone morphogenetic protein receptor type) Activin receptor and the Smad family transcription regulators, which form cooperative complexes that can activate specific genes with other DNA-binding and coactivator (or co-repressor) proteins. The components of these signaling pathways are highly conserved among insect species, and few differences have been found.
