# Supplementary material for: The Genome of Anopheles darlingi, the main neotropical malaria vector
Source: Nucleic Acids Res. 2013 Jun 12;41(15):7387–400. doi: 10.1093/nar/gkt484 (PMC3753621; doi:10.1093/nar/gkt484)
Supplement: Supplementary Data [file supp_gkt484_nar-00257-h-2013-File006_updated.zip › S-I.docx]

**S-I – Circadian rhythm**

Sequences from proteins related to the circadian cycle of *D. melanogaster* *(timeless, cycle, clock, timeout and period)* were retrieved from GenBank and were used on a Blastp search using the *An. darlingi* genome database. The best obtained hits were then used on a new Blastp search against the GenBank non-redundant data set (nr), looking for the best hits with other dipteran species that are vectors of human diseases.

The *timeless, cycle, clock*, *timeout* and *period* proteins from *An. gambiae, Cu. quinquefasciatus* and *Ae. aegypti* were retrieved from GenBank, were aligned with the homologous *An. darlingi* proteins by ClustalW and were compared by the neighbor-joining method (1000 replicates) using the MEGA software, as formerly described [104]. Homologous *D. melanogaster* proteins were used as outgroups.

Sequences from the two *Anopheles* species cluster together in all analyses. *Anopheles* species were clearly separated from *Aedes* and *Culex* with high bootstrap support, except for the *timeless* and *timeout* trees, in which *D. melanogaster* was clustered with *Anopheles* and *Culex* and between *Anopheles* and *Aedes*, respectively. The unexpected result obtained for the *timeless* proteins could be related to the short *Cu. quinquefasciatus* sequence, most likely resulting from an incorrectly predicted start codon.


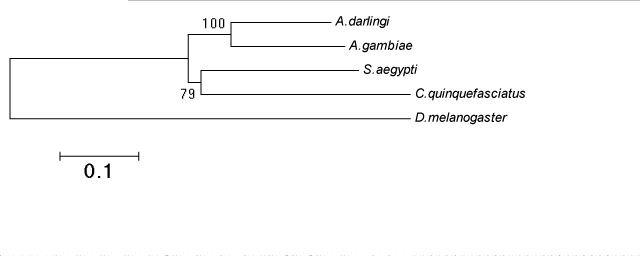

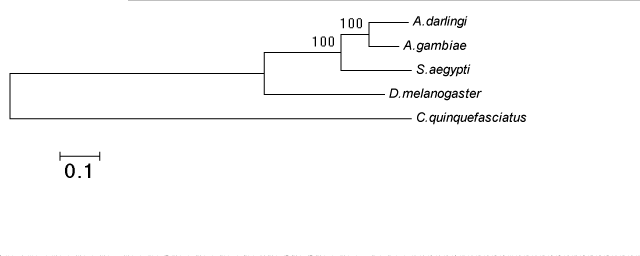

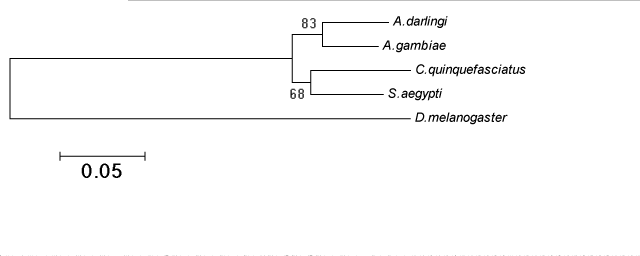

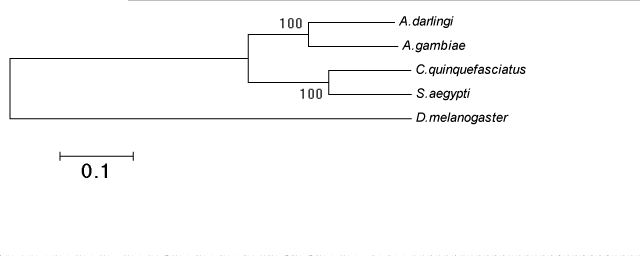

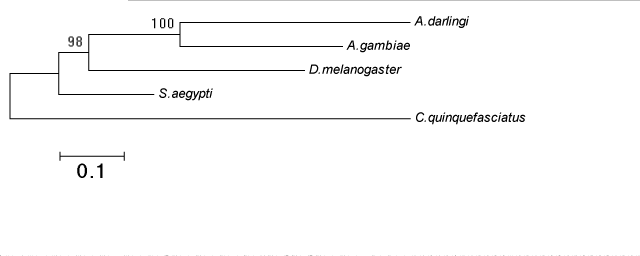


**A**

**B**

**C**

**D**

**E**

**Figure S-I1.** ***Anopheles darlingi* circadian cycle genes dendograms**. Dendograms resulting from the neighbor-joining analysis of the **(A)** *timeless ,* **(B)** *cycle,* **(C)** *clock*, **(D)** *timeout* and **(E)** and *period* genes of *Anopheles darlingi* aligned to homologous genes from dipteran vectors of human diseases (*Anopheles gambiae*, *Culex quinquefasciatus*, *Aedes aegypti*). Homologous *Drosophila melanogaster* genes were used as outgroup. Bootstrap values (1000 replicates) are indicated in tree nodes.

TIMELESS

**10 20 30 40 50 60 70 80 90 100**

**....|....|....|....|....|....|....|....|....|....|....|....|....|....|....|....|....|....|....|....|**

***Anopheles darlingi***  -----------------------MEWLLANPQINSTFGSLGTFVDDAFHVREDCLDTLEEIICKLAVED---ATLRTFRRAIGFGQNVKNDIVPLL----

***Anopheles gambiae***  -----------------------MEWLLANPQINSTFGSLGTFEDDAYHLSEDCLVTLEEIICKLAVED---ATLRTFRRAIGFGQNVKNDIVPLLPA-R

***Culex quinquefasciatus***  -----------------------MAVLLAD--IDATCSNLGHDDGVKYHMEPGTINGLKHLIWILKREG----EDNEYRRYIGQKKVMQTDLIPMLMSNP

***Aedes aegypti***  -----------------------MEWLLANPQINSTFGSLGSFVDDIYLVNDDALRTLEEIICKLALED---TSLRTFRRAIGFGQNVKNDILPLLPA-R

***Drosophila melanogaster*** MSRVRQLHNHIWNNQNFDKVKSVMDWLLATPQLYSAFSSLGCLEGDTYVVNPNALAILEEINYKLTYEDQPARTLRTFRRAIGFGQNVRSDLIPLL----

**110 120 130 140 150 160 170 180 190 200**

**....|....|....|....|....|....|....|....|....|....|....|....|....|....|....|....|....|....|....|....|**

***Anopheles darlingi***  VNAKDAKIIDTTIRLLVNLTVPVECLLPVDPARLVSKSEIGRHTIFELNK---LLITSKEAFVEWKTTKAVIDHMK---GILERDSKLSIQQCDSVNN-C

***Anopheles gambiae***  VNAKDVKIIDTTIRLLVNLTVPVECLLPVD---LVSKSEIGRHTIFELNK---LLITSKEAFVDWKTTKAVIDHMKPARGILERDTKLSIQQCDSVNN-C

***Culex quinquefasciatus***  ARFDNPEVADVLLRLIVNLTYPVLLLYNGN----YPKDSVGRRNFHRLVE---ILQTYKEAFAVQQAWIALGDRLQKVLPARKMDWAERTEEQELIIERI

***Aedes aegypti***  VNTKDAEIIDTTIRLLVNLTVPIECLLPVE---LFSKSEIGCHTIFELNK---LLVTSKEAFIEWKTTKAVVDHMKPARTILEKDSKLSIDNCDSINN-C

***Drosophila melanogaster*** ENAKDDAVLESVIRILVNLTVPVECLFSVD---VMYRTDVGRHTIFELNKPARLLYTSKEAFTEARSTKSVVEYMK---HILESDPKLSPHKCDQINN-C

**210 220 230 240 250 260 270 280 290 300**

**....|....|....|....|....|....|....|....|....|....|....|....|....|....|....|....|....|....|....|....|**

***Anopheles darlingi***  LLLLRNILHVPEMGTGAGSHAG-SYPTAGHNTSFQNQIIWNPARLFTQSVDKL---LIYLMSCTQRAYWGVTMAQLVALMYKDQHISTLQKLLNLWFEST

***Anopheles gambiae***  LLLLRNILHVPEMGTGAGGHGGSCYPTAGHNTSFQNQIIWN---LFTQSIDKLPARLIYLMSCPQRAYWGVTMAQLVALMYKDQHISTLQKLLNLWFEST

***Culex quinquefasciatus***  LMLIRNILQVPS--------CVEAENRYEKAASVHDQVLWA---LHQTGILNLVLYILGSEHEPARHQYHLHSMEITCLIFREQTAISLADAQLTRTAAE

***Aedes aegypti***  LLLLRNILHIPEGNATSG--PGPHHQNSNHNTSFQNQIIWN---LFTQSVDKLLIPARHLMSCPQRAYWAVTMAQLIALMYKDQHMSTLQKLLNLWFEST

***Drosophila melanogaster*** LLLLRNILHIPETHAHCVMPMMQSMPHPARGISMQNTILWN---LFIQSIDKL---LLYLMTCPQRAFWGVTMVQLIALIYKDQHVSTLQKLLSLWFEAS

**310 320 330 340 350 360 370 380 390 400**

**....|....|....|....|....|....|....|....|....|....|....|....|....|....|....|....|....|....|....|....|**

***Anopheles darlingi***  -------LSESSE---DNESNTSPPKQCSGDSSPML---TSDPTSDSSDNG-------------------SGNMPTSMSPARKDSMSLQSETSQTTRMVG

***Anopheles gambiae***  -------LSESSE---DNESNTSPPKQCSGDSSPMLPARTSDPTSDSSDNGECLR-RAGTGLMSAICILGSGKMPSSMS------KECASEAPQVTRMVA

***Culex quinquefasciatus***  KNRDELELIMSRKREKAHQQVRIPVARHSRFGGTYVPARVENMKSISDNNLICHQSLQN--ALKLEFDTDKAPVKKSFRHVKESGTVERKSAFSVRLFLR

***Aedes aegypti***  -------LSESSE---DNESNTSPPQQCSGDSSPMLTSPARDPTSDSSDNGNYTANIILFGVIDRDFSTGSGKMPSSIS-------KATDEVDQASRKTA

***Drosophila melanogaster*** -------LSESSEPARDNESNTSPPKQGSGDSSPML---TSDPTSDSSDNGSNGR------GMGGGMREGTAATLQEVSRKGQEYQNAMARVPADPARKP

**410 420 430 440 450 460 470 480 490 500**

**....|....|....|....|....|....|....|....|....|....|....|....|....|....|....|....|....|....|....|....|**

***Anopheles darlingi***  DYPTQIIFSRAIKSHQ---MYHQTLKDQGCVLVTGNKPSGMG---LQQQSSSQQQQQSPQQHQQLHHHQQQCDHHQQRTINSATDAQEPARVGRNFTKKS

***Anopheles gambiae***  DFPTQIILSRAIKSHQPARMYHQTLKAN----AASSDGCVLG---AGRPAAVRVRVKATELQEVSRNFVCSCALCWVFNLLTFFCSPKSPKVAQ------

***Culex quinquefasciatus***  QFCIEILRASYPARNNLVRQVRRVLERHAGQEAGGGHDDSYLLWAIRFFMEFNRVYKFDLELVTGSYPARRTMYELVARFKVVSPARGSPGVPWMDPTVN

***Aedes aegypti***  EYPSQIIMARVIKSHQMYPARHQSIKVIEGVSCFVKHPSCIKPVTQSEPSSTKQVIDSKQVKPLSVSPDLLDEIHTVRPSLQIQPLQKSPTPARPVSSSP

***Drosophila melanogaster*** DGSEEASDMTGNDSEQPGSPEQSQPAGESMDDGDYEDQRHRQLNEHGEEDEDEDEVEEEEYLQLGPASPAREPLNLTQQPADKVNNTTNPTSSAPQGCLG

**510 520 530 540 550 560 570 580 590 600**

**....|....|....|....|....|....|....|....|....|....|....|....|....|....|....|....|....|....|....|....|**

***Anopheles darlingi***  KSAAKGSLESLVAPVGPTFSKQQPASSGPKETAPAMAADTAPTTITTTATSTTTTSTASASPMNQQKQSQVSLSENS---DCGYGTQVE-KESISPA-RT

***Anopheles gambiae***  -----------AAPARPSVTRIRPAAARRR----ACFANSRPP------------AAKSSDEGEQQAVEWVTISENS---DCGYGTQVE-KESIS----T

***Culex quinquefasciatus***  SAASG------NGEVLSDGGFRRHTETRESRWRGVNLQEQKSSATALLRDKFSIHRNCPQNPPPARFPSPNRTATVG----QGCRSPVPRLECVTRTRCT

***Aedes aegypti***  KS---------ETEISTSEGANKPACSGDNNVFTQPGEDSIPTN----SNTKAKLPASTTNGQTQKEKNQISQSETSPARDCGYGTQVE-KESIS----T

***Drosophila melanogaster*** NEPFK---PPPPLPVRASTSAHAQMQKFNESSYASHVSAVKLGQPARKSPHAGQLQLTKGKCCPQKRECPSSQSELS---DCGYGTQVENQESIS----T

**610 620 630 640 650 660 670 680 690 700**

**....|....|....|....|....|....|....|....|....|....|....|....|....|....|....|....|....|....|....|....|**

***Anopheles darlingi***  SSNEDDTPQQ---KPVHQKPPSNQKQRFNAANKQRNPVSVQEKKELRRKKLVKRGKSN---IINMKGLMHHVPTDDDISHILKEFT---VDFLLKGYGYL

***Anopheles gambiae***  SSNEDDSPHQPARKPVHQKPPSNQKQRFNAANKQRNPVSVQEKKELRRKKLVKRGKSN---IINMKGLMHHVPTDDDISHILKEFTPARVDFLLKGYGYL

***Culex quinquefasciatus***  FRPFSPSAAALVPVWTHRNPPKTFERARERRSHDRTRLRAPARALYQAEAPPSREGSE--TADSTSGWSENPPKFGKLTNCCQNSRKLQARTFKNGQKFV

***Aedes aegypti***  SSNEEDGPNQ---KPLHQKPPPNQKQRFNAANKSRNPTTIHEKKELRRKKLVKRSKSNPARIINMKGLMHHAPSDDDISHILKEFT---VDFLLKGYGFL

***Drosophila melanogaster*** SSNDDDGPQG---KPQHQKPPCNTKPRP-ARNKPRTIMSPMDKKELRRKKLVKRSKSS---LINMKGLVQHTPTDDDISNLLKEFT---VDFLLKGYSYL

**710 720 730 740 750 760 770 780 790 800**

**....|....|....|....|....|....|....|....|....|....|....|....|....|....|....|....|....|....|....|....|**

***Anopheles darlingi***  VQELHTQLPARLSDLQVQIDTSHFFWLVTYFLKFATQ---LELDLEHINSVLSFDIISYLTYEGV---MLCEQLEQLSRAAETDIKPC---LRRIHLVVT

***Anopheles gambiae***  VHELHTQL---LSDLQVQIDTSHFFWLVTYFLKFATQ---LELDLEHINSVLSFDIISYLTYEGVPARMLCEQLEQLSRATETDIKPC---LRRIHLVVT

***Culex quinquefasciatus***  FVIRNAGTP--------------------SYGDLQPARLIAAFDRNSLNRFAHLRTANPSKFAAN-----ALLLAFRPRNPPKNRTTN---LFRDARVDL

***Aedes aegypti***  VQELHAQL---LSDLQVQIDTSHFFWLVTYFLKFASQPARLELDLEHINTVLSFEIVSYLAYEGV---MLCEQLEQLNREPHNDLLPC---LRRIHLVVT

***Drosophila melanogaster*** VEELHMQPARLLSNAKVPIDTSHFFWLVTYFLKFAAQ---LELDMEHIDTILTYDVLSYLTYEGVS---LCEQLELNARQEGSDLKPARPYLRRMHLVVT

**810 820 830 840 850 860 870 880 890 900**

**....|....|....|....|....|....|....|....|....|....|....|....|....|....|....|....|....|....|....|....|**

***Anopheles darlingi***  AIREFLQALDTYKKSTHLTVPAREDKEKLKMLQQQISSTEDLRC---LFVLLLRCYNPNIQSRQY---LQDLIVTNHTLLLLLDGVRELQQSDALPG-DM

***Anopheles gambiae***  AIREFLQALDTYKKSTHLTK---EDKEKLKLLQLQISCTEDLRCPARLFVLLLRCYNPNIQSRQY---LQDLIVTNHTLLLLLDGVRELT-ADGNPG-DM

***Culex quinquefasciatus***  EIQSPLRCRPRRRQLEPARQNGAENPPKSPHEKLPLLPLVHYRSAYGSFLLLLLMLEGPCASCWLASKFVSGRPG-VPRQRLAAKSSAMAPARAFRRHKK

***Aedes aegypti***  AIREFLQALDTYKKSSPARHLTKEDKDRLKLLQLQICATEDLRC---LFVLLLRCYNPNVQSRQY---LQDLIVTNHILLLLLDGVQGIAKANNGPARDM

***Drosophila melanogaster*** AIREFLQAIDTYNKVTHLNE---DDKAHLRQLQLQISEMSDLRC---LFVLLLRRFNPSIHSKQYPARLQDLVVTNHILLLILDSSAKLG---GCQTIRL

**910 920 930 940 950 960 970 980 990 1000**

**....|....|....|....|....|....|....|....|....|....|....|....|....|....|....|....|....|....|....|....|**

***Anopheles darlingi***  LGHIKQFATVEIMHQYGLLLED---FRENGAYVPARNDCIFTMMHHV---GGDLGQINVLFQPNILKTYSQIWETEY---ELCDDWSDLIEYVIH--KFI

***Anopheles gambiae***  LGHIKQFATVEIMHQYGLLLEDPARFRENGAFV---NDCIFTMMHHV---GGDLGHINVLFQPSILKTYSQIWETEY---ELCDDWSDLIEYVIH--KFI

***Culex quinquefasciatus***  YRTVLEFLVLQKASTEATYGHTAGIFKPSLEAP-LSTHGGTQVGHNGGITAQEAGSMQAVLTRGPARRRSRPWRAAPRPLALFRGGTQAANHHYHGHEAA

***Aedes aegypti***  LVHIKQFATVEIMHQYGLLLED---FRDNGAFV---NDCIFTMMHHV---GGDLGQINVLFQPNILKTYSQIWETEYPAREICDDWSDLIEYVIH--KFI

***Drosophila melanogaster*** SEHITQFATLEVMHYYGILLED---FNNNGEFV---NDCIFTMMHHIPARGGDLGQIGVLFQPIILKTYSRIWEADY---ELCDDWSDLIEYVIH--KFM

**1010 1020 1030 1040 1050 1060 1070 1080 1090 1100**

**....|....|....|....|....|....|....|....|....|....|....|....|....|....|....|....|....|....|....|....|**

***Anopheles darlingi***  NTPQPAP---LTLSTTLPEIS---TQLLSGNLLGSWTQEEKDSLHWYYVQPARCRQSKCLVADIL---KLFQENGNQQKTRLSIIEQL---LEQDIIALG

***Anopheles gambiae***  NTPQPAPARPLTLSTTLPDIG---TQLLSGNLLVTWTQEEKDSLHWYYVQ---CRQSKCVVADIL---KLFQENGNQQKTRLSIIEQLPARLEQNIVTLV

***Culex quinquefasciatus***  QPVQHTAGGAQAALPQQKTTQHTVHTHKAARTLQLRLPARAATRPHSYDPRWAAHEPLTPAHPSNEDESLWKPWARSPLRRMLVPARRVLQLDQSIQLLL

***Aedes aegypti***  NTPQPSP-----LASTLPEIS---TQLLSG--INPWTQEEKDSLQWYYVQ---CKQSSCLVADILPARNLFQESGNQQKTRVSIIEQL---WEQDIISLM

***Drosophila melanogaster*** NTPPKSP--LTIPTTSLTEMTKEHNQEPARHTVCSWSQEEMDTLYWYYVQ---SKKNNDIVGKIV---KLFSNNGNKLKTRISIIQQL---LQQDIITLL

**1110 1120 1130 1140 1150 1160 1170 1180 1190 1200**

**....|....|....|....|....|....|....|....|....|....|....|....|....|....|....|....|....|....|....|....|**

***Anopheles darlingi***  QYDELMKLE---NPEYERNVQTPALSVASLDSTGRRDDGDSKSSSPKAVDDIQVLRDRLQKEPARNRGKLVAWLQKSLLDCCFVKLNLLSGNDFDATSVV

***Anopheles gambiae***  QYDDLMKVE---NPDYERNVQTPALSVASADSP-KPEDGDSKSSS-KAVDDIQVLRDRLQKENRGKPARLIAWLQKSLLDCCFVKLNLLSGN-IYVTAGI

***Culex quinquefasciatus***  MPNRFFDDEPPARYDMMIDDDDGGVERDWQQVSLYTVAAGCWPIVGIHRSLVHIRLEGPCSKHRVEQEQKYKFDLELVRVCVVPARGESSIMCVVVCCAT

***Aedes aegypti***  QYDDLMKVE---NPGYERNVQTPAFSVASCNSGKRDDENKTPSMPARAIDDIQVLRDRLLKENKSN---MIVWLQKSLLNCCFVKLNVLSGN-MYISSSI

***Drosophila melanogaster*** EYDDLMKFEPARDAEYQRTLLTTPTSATTESGIEIKECAYG-----KPSDDVQILLDLIIKENKAQ---HLLWLQRILIECCFVKLTLRSPA-----RGL

**1210 1220 1230 1240 1250 1260 1270 1280 1290 1300**

**....|....|....|....|....|....|....|....|....|....|....|....|....|....|....|....|....|....|....|....|**

***Anopheles darlingi***  NGVLSVSVMEPVSYHCILKK---KSIPVVPWNQDQFAVLSYQP---FILLLHKLGFHLPADAKKMFVRIPEPARFWTADILYNIALKLGPLDKSILKFDL

***Anopheles gambiae***  GGSTGVVVMEPVSYHCILKK---KSIPVVPWNQDQFAILSYQPPARFILLLHKLGFHLPADAKKMFVRIPE---FWTADILYNIALKLGPLDKSILKFDL

***Culex quinquefasciatus***  STKVGDFGKISSKLGKARPPPPKPGHHTPPEARTEAFLCSYTEPYPQICIRSLIMLPARLLLLQVITRSESLSVPCFHWIITRIEHYIDMMRSDKTRARL

***Aedes aegypti***  DENSNVVVMESVSYHCITKKQPARSIPVVPWNQEQFSILTYQP---FVLLLHKLGFHLPADAKKMFVRIPE---FWTADILYNIALKLGPVEQSIIKFDL

***Drosophila melanogaster*** KVPEGDHIMEPVAYHCICKQ---KSIPVVQWNNEQSTTMLYQP---FVLLLHKLGIQLPADAGSIFARIPDYWTPARPETMYGLAKKLGPLDKLNLKFDA

**1310 1320 1330 1340 1350 1360 1370 1380 1390 1400**

**....|....|....|....|....|....|....|....|....|....|....|....|....|....|....|....|....|....|....|....|**

***Anopheles darlingi***  --------------------KYLNKVLSMEKQA-------------------------KADSCPPT---DSRLENFG-LSRFTSQIT---TNWLDVVMRS

***Anopheles gambiae***  --------------------KYLNKVLSMEKQA-------------------------KADPCPPARSNDARLENFG-LSRFSPQIT---TNWLDVVMRN

***Culex quinquefasciatus***  WARRLHVAVQAYR----EMLQSLNTLQKFQDDK-----------------PARAKDLFAMLQNNVFYVLEYREVILHLLINYNENDS--TRAYLRDVIET

***Aedes aegypti***  PA-----------------RNCLQKVLLMEKDV-------------------------KVEPGPMS---NSTLDGYTVLASFTSDTN---TGWLHVVMRN

***Drosophila melanogaster*** SELEDATASSPSRYHHTGPRNSLSSVSSLDVDLGDTEELALIPEVDAPARAVEKAHAMASTPSPSEIFAVPKTKHCNSIIRYTPDPTPPVPNWLQLVMRS

**1410 1420 1430 1440 1450 1460 1470 1480 1490 1500**

**....|....|....|....|....|....|....|....|....|....|....|....|....|....|....|....|....|....|....|....|**

***Anopheles darlingi***  KA----------------AQSKR-KFDLPGPSKVIDAANATHPPARGASL------------------KPGSKSTSQTKILHDLSIIVESN-------DD

***Anopheles gambiae***  KA----------------VQSKRNKLDLPGPSKVIDTANATHPSATLKSG------------------GKPAAPSPARKMLHDLSIIVESN-------DD

***Culex quinquefasciatus***  AHMFFKMLEKYCQGTGGVRVQSKKRPARAKPKRNNNKDKQGKASQQPEMDLES---------------LEGLWLTMAGEVSTCLANQITLPEEDHPIPFD

***Aedes aegypti***  KN----------------VDTAR-LVDMPGPSKVIDSAPARNVVHPSAKA------------------ASSPTVITQSKLTHDLSVIVEASNDDEVPTED

***Drosophila melanogaster*** KCNHRTGPSGDPSDCIGSSSPARTTVDDEGFGKSISAATSQAASTSMSTVNPTTTLSLNMLNTFMGSHNENSSSSGCGGTVSSLSMVALMSTGPARAAGG

**1510 1520 1530 1540 1550 1560 1570 1580 1590 1600**

**....|....|....|....|....|....|....|....|....|....|....|....|....|....|....|....|....|....|....|....|**

***Anopheles darlingi***  DDDDAVLPEDDDGLDSSEVPVLEEHD----------------VVS--------ACETASVASDLTR--------------MYVSDEDDKHDIVP----PI

***Anopheles gambiae***  DDD-ELPADEDDGLDSTEVPVLEEHD----------------VVS--------ACETASVASDLTR--------------MYVSDEDDKHDIVPP-ARPI

***Culex quinquefasciatus***  AASDVPIDDQKYVPARPAMESFRLARINPSSLLLFNQLRGDCMIRIHSLLRDGKYEHAIILMRSARLSHPGHLSVKVWFGEMKPNSPARCALIRTSDFWD

***Aedes aegypti***  DVNADDDDDDDDDDDALEGPCLEEHD----------------VVSPAR-----ACETASVASDLTR--------------MYVSDEDDKHDIVP---SSI

***Drosophila melanogaster*** GGNTSGLEMDVDASMKSSFERLEVNGSHFSRANNLDQEYSAMVASVYEKEKELNSDNVSLASDLTRPAR-----------MYVSDEDDRLERTEIRVPHY

**1610**

**....|....|**

***Anopheles darlingi***  LPARPAR---

***Anopheles gambiae***  LPARPAR---

***Culex quinquefasciatus***  SPARPARPAR

***Aedes aegypti***  LPARPAR---

***Drosophila melanogaster*** HPARPAR---

TIMEOUT

**10 20 30 40 50 60 70 80 90 100**

**....|....|....|....|....|....|....|....|....|....|....|....|....|....|....|....|....|....|....|....|**

***Anopheles darlingi***  MVIGLHTIDAISTSLGYGDIEKYYPDPQAIDGLKHLIWILRNDGPDHEFRRYIGEKRLVQTDLLPFLLHS---FHEPDVADVVLRLLVNLTFPTDLIYKE

***Anopheles gambiae***  MSAYFADIDAVCSSLGWMDGEVYKMDPEAVQGLKHLIWILKQDRPAHECRRYIGGKRIVQTDLIPMVISNPARFDKPDVVDVLLRLLVNLSFPTLLLYNG

***Culex quinquefasciatus***  MAVLLADIDATCSNLGHDDGVKYHMEPGTINGLKHLIWILKREGEDNEYRRYIGQKKVMQTDLIPMLMSNPARFDNPEVADVLLRLIVNLTYPVLLLYNG

***Aedes aegypti***  MSILLADIDATCSALGWDDGCKYHMEPDAIMGLKHLIWILKRDSDDHEYRRYIGQKKVMQTDLIPMLMSNPARFDNPDVADVLLRLIVNLTYPTLLLYQG

***Drosophila melanogaster*** MSILLADIDATCAALGYSDGQKYQAEPDAAEGLKHLIWILRRDLDNHEYRRHLGRSKVLQTDLVYMLPDYPARVHHEELSDLLIRLLVILTNPTLLLYRE

**110 120 130 140 150 160 170 180 190 200**

**....|....|....|....|....|....|....|....|....|....|....|....|....|....|....|....|....|....|....|....|**

***Anopheles darlingi***  NHPPARKNASDRKKMLQLMEIAEGYKEAFTVNAVWSVLGEKMQSILRIKSLE---RSEAQAITVERILMLARNILQVPANVEKEKQLDNEDSLHDRLVWA

***Anopheles gambiae***  NYP---KDAAEQRKFIRLMEICEEYKEAFSLKSVWSVLGDRLEKILPARHTDWALRSEADGLIIERILVLIRNVLQVPANVEREKRFDNDASQHDCLLWA

***Culex quinquefasciatus***  NYP---KDSVGRRNFHRLVEILQTYKEAFAVQQAWIALGDRLQKVLPARKMDWAERTEEQELIIERILMLIRNILQVPSCVEAENRYEKAASVHDQVLWA

***Aedes aegypti***  TQP---KDAVGRRNYLHLVEILQGYKEAFAVQQAWAALGDRLQKVLPARQVDWAERIEEQELIIERILTLTRNVLNVPSNMDMEKRFDNDASLHDQVLWA

***Drosophila melanogaster*** GAP---KDNHGRKVFMELIDILQGYKAAFAKDKVWSALFEKLKQALPAREIAFAIRSEEQNLLIERILVLVRNVLQVPANPEAECRADNDASLHDQVIWA

**210 220 230 240 250 260 270 280 290 300**

**....|....|....|....|....|....|....|....|....|....|....|....|....|....|....|....|....|....|....|....|**

***Anopheles darlingi***  LDQTCTINIPARVSYILGSSSE---QQYFMHALEIIYLVFREQQPANLVNATMQRIASEKQEDEVKLLEALQ---------QPKTAKLPLPPRHS---RF

***Anopheles gambiae***  LHQAGILDI---ILYILGSPHEPARNRFLIHTLEILSLAYREQSAVHLADATLQRTNTEKKTDELLLIKARQPTAAAAAATTPAALGKPPAGRHSPARRF

***Culex quinquefasciatus***  LHQTGILNL---VLYILGSEHEPARHQYHLHSMEITCLIFREQTAISLADAQLTRTAAEKNRDELELIMSRK-------REKAHQQVRIPVARHS---RF

***Aedes aegypti***  LHEAGVLDL---ILYILGSEHEPARHQYHLHALEITCLAYREQSATSLADASYQRSATEKHRDELELLAARK-------REKAKQLSRIPAARHS---RF

***Drosophila melanogaster*** LHQTGMLDL---VLFVISSPDEPAREQFHLHGLEIVCLLFREQSAESLADASLQRSLSEKQRDQQELLAARR-------RERARRQARPPPGRHS---RF

**310 320 330 340 350 360 370 380 390 400**

**....|....|....|....|....|....|....|....|....|....|....|....|....|....|....|....|....|....|....|....|**

***Anopheles darlingi***  RGTYMSG---SVKSISDNAAIAHQSLSKIIPARHADLGIEKERAKKSARLTRTVDEVERKSILSVRMCLRQYCV----EILNLFNNIVRQAKRYLGS--A

***Anopheles gambiae***  RGTYTFN---NVKSISDSDAVCHQSLGKIMR---MDFSSEKHRQKVSFRNAKETETFERKSIFSVRLFLREYCMPAR-EILRVYNNMVRQARRYLNQ--H

***Culex quinquefasciatus***  GGTYVPARVENMKSISDNNLICHQSLQNALK---LEFDTDKAPVKKSFRHVKESGTVERKSAFSVRLFLRQFCIEILRASYPARNNLVRQVRRVLER-HA

***Aedes aegypti***  GGTYVPARIQNMKSISDNDLICHQSLQKALQ---MEFDTDKKRVKKSFRNVRETGTIERKSAFSVRLFLREYCIEVLRSSYPARNNLVRQVRKILERNAH

***Drosophila melanogaster*** GGTYVPARIRNMKSVSDRDVICHQALERVSS---IDFDREKQQQKRSHRHIQEEAQVTRRSAFTVRLCLREYCIEVLRSAYPARNTLVRQVRRVLER--N

**410 420 430 440 450 460 470 480 490 500**

**....|....|....|....|....|....|....|....|....|....|....|....|....|....|....|....|....|....|....|....|**

***Anopheles darlingi***  DQLSSDFHDDTYLLWAIRFFMAFNRMNGMNIELIGEALSPARMNTFHWIISRIES---YNSSITCDKTRKSIWSKRLHNAVQTYDEMLCNLRALSLKED-

***Anopheles gambiae***  GTSVSEFHDDSYLLWAIRFFLEFNRHSGFKIELVSESLN---IDTFHWTVTRMPARDTYTENITTDKTRKAVWARRLHLTIQTYRELLNNMHALEKIKD-

***Culex quinquefasciatus***  GQEAGGGHDDSYLLWAIRFFMEFNRVYKFDLELVTGSYP------ARRTMYELVARFKVVSPARGSPGVPWMDPTVNSAASGNGEVLSDGGFRRHTETRE

***Aedes aegypti***  GQEASGGHDDSYLLWAIRFFMEFNRASGFKVELVSESLS---VPCFHWIITRIEHFVPARDMMGSDKTRARLWARRLHVAVQAYREMLHSLQALQKMPD-

***Drosophila melanogaster*** AGSSS--HDDSYLLWAIRFFMEFNRLSGLQLQLVSESLS---VQCFHWVLTRMQHDMDMIVPARSDKKQARLWAKRLHVALKTFRELLQSLLALQKLKDD

**510 520 530 540 550 560 570 580 590 600**

**....|....|....|....|....|....|....|....|....|....|....|....|....|....|....|....|....|....|....|....|**

***Anopheles darlingi***  PESKELLSMLQNNIFYAVEYRETVVHL---LTNYNESYQTRNFLRSVVEPARVANRFYSLLEKFCDGTVRVQTRVK----SKRKKQKGRKAGKSDRPPIT

***Anopheles gambiae***  TAASELLSVLQNNIFYVVEYRETTLHLPARLTSFKEALHTKAYLRDVIE---VAHLFFSMLQRFCKGTVRVQERVK----AKRKKAKKQPRKKTTEE--E

***Culex quinquefasciatus***  SRWRGVNLQEQKSSATALLRDKFSIHRNCPQNPPPARFPSPNRTATVGQG---CRSPVPRLECVTRTRCTFRPFSPS---AAALVPVWTHRNPPKTFERA

***Aedes aegypti***  EKAKDLFLMLQNNIFYVLEYREVVLHLLINYPARKETNSTRAYLRDVVE---TAHMFFKMLEKYCQGTVRVQSKKRAK--QKRNKNRQDNAGSRKDPELN

***Drosophila melanogaster*** NNARALFDMLLNNVCYVLEYRETVLHLLMNYNEAPARHSTKVFLRDVVE---TANVFIKMMERFCQDSVVVQDKKRGRGASRKKKQAATKSKPPAAPQPT

**610 620 630 640 650 660 670 680 690 700**

**....|....|....|....|....|....|....|....|....|....|....|....|....|....|....|....|....|....|....|....|**

***Anopheles darlingi***  PESLEFDWLSM---APKIAAVLP-QVHATEENLPTPFDVASSVPIDDQKGDCMIRIHRLLPARRDEKYEDALKLMYAARSVWTN---EDCFGSMHATADE

***Anopheles gambiae***  EENIENRWLPARQEAPTIASLLE-NGELKQQELPTPFDSASSVPVEEQKGDCLKSIHSLL---RKKNYEQAIKMLYAARSVWTTSSPARCFGSETATPDE

***Culex quinquefasciatus***  RERRSHDRTRLRAPARALYQAEAPPSREGSETADSTSGWSENPPKFGKLTNCCQNSRKLQARTFKNGQKFVFVIRNAGTPSYGDLQPARLIAAFDRNSLN

***Aedes aegypti***  ADELEELWLPARTMAGEVSTCLANQITLPEEDHPIPFDAASDVPIDDQKGDCMIRIHSLL---RDGKYEHAIILMRSAREVWPKDPARDCFGSLASAPED

***Drosophila melanogaster*** EEELSSKWAEPARLATEVCSLLSTELEMPEDEQPLPFDAASEKSIDDQREDCMIRINKLL---RSEKLDQAIALLRAAREVWPENEPARVFGAISAAPED

**710 720 730 740 750 760 770 780 790 800**

**....|....|....|....|....|....|....|....|....|....|....|....|....|....|....|....|....|....|....|....|**

***Anopheles darlingi***  DLMCLK----EIFMANIAEDHETQHSD-EDEEDDDEEDEVEEYEAR-------TKYGEKDFKF---EDFSKRLVDPKVVRNPARCVIVLSDWETIKTPTM

***Anopheles gambiae***  DLITLQ----DIFAANLSSATDADDNDGEVQEDSEEEDELEEEGKQCLEEFEIVKHEEKDFKFPAREEFSKRLVDHRVVY---ACTTVLTDWEKIKTTCL

***Culex quinquefasciatus***  RFAHLRTANPSKFAANALLLAFRPRNPPKNRTTNLFRDARVDLEIQSPLRCRPRRRQLEPARQNGAENPPKSPHEKLPLLPLVHYRSAYGSFLLLLLMLE

***Aedes aegypti***  ELMLLK----EIFLANLPRNEFIDQNE-ADDYDDEEEDEEED-----------NHMTEIDFRF---EDFAKRLLNPKPARVVHACILVLSDWEEISVESL

***Drosophila melanogaster*** ELLLLR----EIFMSNITTVESEDKENEKDQKQDEYEDEDDDLDNEEYEEQD-NGLTEKTFKFDPARDFARRLLNPKIVR---ACTLVLSDWADIPTRSL

**810 820 830 840 850 860 870 880 890 900**

**....|....|....|....|....|....|....|....|....|....|....|....|....|....|....|....|....|....|....|....|**

***Anopheles darlingi***  KAAIHILHRIAVQHKTPAMLFQASLFRIFQRALHAK---PSEHTAPLQQLGLY---VIRQLQKKAP-----DNEVIFAELLFYKSAKICYGLEEGPARYD

***Anopheles gambiae***  KAAVTLLYRICVEHKMPSMLFQVSLLRIFQAVLNAIEDEPARHSRELRRLAIY---TVRQLQQKVLTGAEDDGGLLFAELLFAKTSRIACALEIG---YD

***Culex quinquefasciatus***  GPCASCWLASKFVSGRPGVPRQRLAAKSSAMAPARAFRRHKKYRTVLEFLVLQKASTEATYGHTAG----IFKPSLEAPLSTHGGTQVGHNGGITAQEAG

***Aedes aegypti***  KATVTILHRIAVGSKMPALLFQASLFRIFQRVFHAP---KDAHHEELRRLGVYPARIVRQFTALAP-----NNPKIYAELLFYKSIKEANGIEMG---YE

***Drosophila melanogaster*** KAAVTILHRIAYGCKCAGMLFQAKLFRIFQQVFSVERDVHPARQEELRRLAIF---VVRKFVEVAP-----TNPKIYAELLFYKGIREANELESG---YC

**910 920 930 940 950 960 970 980 990 1000**

**....|....|....|....|....|....|....|....|....|....|....|....|....|....|....|....|....|....|....|....|**

***Anopheles darlingi***  EVFNRPAGAAGSSAKNWSEEQEEE---LR------RLFMENQENPQTDQDVIDWLLDNMIDRTRTRRAVIKKLKELGLIFKAPTKRSNANQHQKD-SWNG

***Anopheles gambiae***  EVKS------TASQKAWTEEQEQEPARLH------RLFMENQENPQTDQDVIDWLLENLIDQTRTRRGVMKKLKEMGLTFKAPTKKSNANRQQKANAWTA

***Culex quinquefasciatus***  SMQAVLTRGPARRRSRPWRAAPRPLALFRGGTQAANHHYHGHEAAQPVQHTAGGAQAALPQQKTTQHTVHTHKAARTLQLRLPARAATR-PHSYDPRWAA

***Aedes aegypti***  DIYG-----------------------------------------------------NNVPARPAR----------------------------------

***Drosophila melanogaster*** DAYE------AGTKGAWTEEQESELRFLF------EPARENQRNPETDKDVIDWILDNLVDKTRNRRTVLKKLKELGLLFKAPTKRSTKSAQSGKNVWQP

**1010 1020 1030 1040 1050 1060 1070 1080 1090 1100**

**....|....|....|....|....|....|....|....|....|....|....|....|....|....|....|....|....|....|....|....|**

***Anopheles darlingi***  EQIERLTQPARLYEEHR--FERNVVKKIRDAFGQAFSRQAIVKQMVR--------------MGLIADESEVKVRKSAASKPNRRR------------KER

***Anopheles gambiae***  DEDPARGKLGVLYEELR--LDKNPLKSIAEAFDKKFTRPAIARRMVY--------------LGLIADVAEIMPSKRRANKERRASGSDGS------SQPA

***Culex quinquefasciatus***  HEPLTPAHPSNEDESLWKPWARSPLRRMLVPARRVLQLDQSIQLLLMPNRFFDDEPPARYDMMIDDDDGGVERDWQQVSLYTVAAGCWPIVGIHRSLVHI

***Aedes aegypti***  ----------------------------------------------------------------------------------------------------

***Drosophila melanogaster*** EEDDELRSLPARYDQHR--IEPDCLERLVNEFAERRSKQQIIKRMLQ--------------IHLIADKSEILPAKKGRGKDKPKKDVEMEGEGDEFDFPA

**1110 1120 1130 1140 1150 1160 1170 1180 1190 1200**

**....|....|....|....|....|....|....|....|....|....|....|....|....|....|....|....|....|....|....|....|**

***Anopheles darlingi***  RDSASSSESYDSEEREEDNDGNDDGPAVVLDQKVAEKMMPARKLMGNSMKPCIDWVSSSMDFVCSSG------AIRDLLRGGRTIYPKEEHQILALKDGR

***Anopheles gambiae***  RDDERNSNSTDEEEEEEKEEEFNQSINKQLNEPLVKKNLKA--LRGDDMKQAVRWIVECFEEVRSLYEDSDPSPAREDTEGGVPMIPIAPYQTAALKKPE

***Culex quinquefasciatus***  RLEGPCSKHRVEQEQKYKFDLELVRVCVVPARGESSIMCVVVCCATSTKVGDFGKISSKLGKARPPPPKPGHHTPPEARTEAFLCSYTEPYPQICIRSLI

***Aedes aegypti***  ----------------------------------------------------------------------------------------------------

***Drosophila melanogaster*** RAEEPMFEDQGYKKPKSKPKKVQKRQMVRTPLDVGTIRALIGQVDSEKYQSAIEWLQECLQDASEDT--EEAVPAREDDDG-VPLLPLMENQKNAMEDGD

**1210 1220 1230 1240 1250 1260 1270 1280 1290 1300**

**....|....|....|....|....|....|....|....|....|....|....|....|....|....|....|....|....|....|....|....|**

***Anopheles darlingi***  FR----QFLLALGMEATN----ASWCIPEEFEAADLKQRIELLQTLCSSRETSPAAPARAD------------------------------RSIEEEDVV

***Anopheles gambiae***  FK----RLLRSLGVMDTGR-QFVYHRIPFQLTPEELKKRMEILTAFCEAP--------------------------------------------------

***Culex quinquefasciatus***  MLPARLLLLQVITRSESLSVPCFHWIITRIEHYIDMMRSDKTRARLWARRLHVAVQAYREMLQSLNTLQKFQDDKPARAKDLFAMLQNNVFYVLEYREVI

***Aedes aegypti***  ----------------------------------------------------------------------------------------------------

***Drosophila melanogaster*** FQ----KVLVALGMQPPISGMEAYWRIPIYLNSADLILRSKILAGEEVDAEPARPEDEAADDEDGEEAEESDEEEDFLEKHSRQRKENIATQQQRKLDSL

**1310 1320 1330 1340 1350 1360 1370 1380 1390 1400**

**....|....|....|....|....|....|....|....|....|....|....|....|....|....|....|....|....|....|....|....|**

***Anopheles darlingi***  VQSNSTQPSVGGVPSEGSD---------------------------------------------------------------------------------

***Anopheles gambiae***  ------------TPARAAD---------------------------------------------------------------------------------

***Culex quinquefasciatus***  LHLLINYNENDSTRAYLRDVIETAHMFFKMLEKYCQGTGGVRVQSKKRPARAKPKRNNNKDKQGKASQQPEMDLESLEGLWLTMAGEVSTCLANQITLPE

***Aedes aegypti***  ----------------------------------------------------------------------------------------------------

***Drosophila melanogaster*** MFNQSDDETEQRAPPKAKDKPKASPAREKKAKRKVKGKAKESKDCNDDGTDKAELEKPNTDDLFNQLRAKRATKIKLSDMALNESTNAAQEADDDAFPAR

**1410 1420 1430 1440 1450 1460 1470 1480 1490 1500**

**....|....|....|....|....|....|....|....|....|....|....|....|....|....|....|....|....|....|....|....|**

***Anopheles darlingi***  --------------EDEMPSF-------------------------------------------------SKRKRSPSIAGKRDRSDSSDSMESSTYANK

***Anopheles gambiae***  --------------EDEE-------------------------------------------------------------AG--DRSVVSPSAASEDEMDK

***Culex quinquefasciatus***  EDHPIPFDAASDVPIDDQKYVPARPAMESFRLARINPS--------------------SLLLFNQLRGDCMIRIHSLLRDGKYEHAIILMRSARLSHPGH

***Aedes aegypti***  ----------------------------------------------------------------------------------------------------

***Drosophila melanogaster*** NFNSEDYRARLLELEDEEQNSKDADENGGDKENITNKSLQRARRVNVIDSDSDNDDDGALPADKTNKRPRPARSDDDGDSGHMDANAEEDSDEEANFLAR

**1510 1520 1530 1540**

**....|....|....|....|....|....|....|....|...**

***Anopheles darlingi***  KSRKRM-------------VVSSDDED--PARPAR--------

***Anopheles gambiae***  NN-------------------HYEKER--PARPAR--------

***Culex quinquefasciatus***  LSVKVWFGEMKPNSPARCALIRTSDFWDSPARPARPERIDPAR

***Aedes aegypti***  -------------------------------------------

***Drosophila melanogaster*** KKPAKVD--GEPAKRRRLAIIDDDDEDDFPARPAR--------

CLOCK

**10 20 30 40 50 60 70 80 90 100**

**....|....|....|....|....|....|....|....|....|....|....|....|....|....|....|....|....|....|....|....|**

***Anopheles darlingi***  MEELKHNPSRGNAFDEEDGFGSAIDIDHHHHHHHHHHHHHGGDSYSAMDEDGDDKDDTK------RKSRNLSEKKRRDQFNLLVNELSSMVSSNSRKMDK

***Anopheles gambiae***  -----------------------------------------------MDEDPDDKDDTK------RKSRNLSEKKRRDQFNLLVNELSSMVSSNSRKMDK

***Culex quinquefasciatus***  ----------------------------------------------------------------------------------------------------

***Aedes aegypti***  -----------------------------------------------MEDDPDDKDDTKRFVVYDSKSRNLSEKKRRDQFNLLVNELSSMVSSNSRKMDK

***Drosophila melanogaster*** -----------------------------------------------MDDESDDKDDTK------RKSRNLSEKKRRDQFNSLVNDLSALISTSSRKMDK

**110 120 130 140 150 160 170 180 190 200**

**....|....|....|....|....|....|....|....|....|....|....|....|....|....|....|....|....|....|....|....|**

***Anopheles darlingi***  STVLKSPARTIAFLKSHNEIAVRSRV---HEIQTDWKPSFLSNEEFTHLILEALDGFIIVFSSTGRVFYASESITSLLGHLPSDLLNMTVYDMVYEDDQ-

***Anopheles gambiae***  STVLKS---TIAFLKSHNEIAVRSRVPARHEIQTDWKPSFLSNEEFTHLILEALDGFIIVFSSTGRVFYASESITSLLGHLPSDLLNMTVYDMVYEDDQP

***Culex quinquefasciatus***  ----------------------------------------------------------------------------------------------------

***Aedes aegypti***  STVLKS---TIAFLKSHNEIPARAVRSRVHEIQTDWKPSFLSNEEFTHLILEALDGFIIVFSSTGRVFYASESITSLLGHLPSDLLNMTVYDMPARVYED

***Drosophila melanogaster*** STVLKS---TIAFLKNHNEATDRSKVPARFEIQQDWKPAFLSNDEYTHLMLESLDGFMMVFSSMGSIFYASESITSQLGYLPQDLYNMTIYDLAYEMDHP

**210 220 230 240 250 260 270 280 290 300**

**....|....|....|....|....|....|....|....|....|....|....|....|....|....|....|....|....|....|....|....|**

***Anopheles darlingi***  --NDLYHILLNPTTIPARVDPLQTGISRENQVTFSCYIKRGTVDYRADVSYEHVQFTGYFR------------SDVDTESLMTTSRF-------------

***Anopheles gambiae***  ARNDLYNILLNPTTV---VDPLQTGISRENQVTFSCYIKRGTVDYRADVSYEHVQFTGYFR------------SDVDTESLMTTSRFPA-----------

***Culex quinquefasciatus***  ----------------------------------------------------------------------------------------------------

***Aedes aegypti***  DQNDLYNILLNPAAV---VDPLQTGISRENQVTFSCYIKRGTADYRTEVSYELVQFTGYFSEYDRWSSCPARGSDVDADSLMTTSRF-------------

***Drosophila melanogaster*** AREALLNIFMNPTPV---IEPRQTDISSSNQITFYTHLRRGGMEKVDANAYELVKFVGYFRN--------DTNTSTGSSSEVSPARNGSNGQPAVLPRIF

**310 320 330 340 350 360 370 380 390 400**

**....|....|....|....|....|....|....|....|....|....|....|....|....|....|....|....|....|....|....|....|**

***Anopheles darlingi***  -SGYTSDADSRLVFVGTGRLQTPQLIREMSIVDNTKSEFTSRHSPARLEWKFLFLDHRAPP---IIGYLPFEVLGTS---GYDYYHFDDLEKVVACHEAL

***Anopheles gambiae***  RSGYTSDADSRLVFVGTGRLQTPQLIREMSIVDNTKSEFTSRHS---LEWKFLFLDHRAPP---IIGYLPFEVLGTSPARGYDYYHFDDLEKVVACHEAL

***Culex quinquefasciatus***  ----------------------------------------------------------------------------------------------------

***Aedes aegypti***  -SGYMTDADTRLIFVGTGRLQTPQLIREMSIVDSSKSEFTSRHS---LEWKFLFLDHRAPARPPIIGYLPFEVLGTS---GYDYYHFDDLEKVVSCHEAL

***Drosophila melanogaster*** QQNPNAEVDKKLVFVGTGRVQNPQLIREMSIIDPTSNEFTSKHS---MEWKFLFLDHRAPARPPIIGYMPFEVLGTS---GYDYYHFDDLDSIVACHEEL

**410 420 430 440 450 460 470 480 490 500**

**....|....|....|....|....|....|....|....|....|....|....|....|....|....|....|....|....|....|....|....|**

***Anopheles darlingi***  MQKGEGTSCYYRFLTKGQQWIWLQTRFYITYHQWN---SKPEFVVCTHRVVSYADVPARMKQMRN--QTVADGK----------FSED---ADSVSVHGV

***Anopheles gambiae***  MQKGEGTSCYYRFLTKGQQWIWLQTRFYITYHQWN---SKPEFVVCTHRVVSYPARADVMKQMRN--QTAGDSK----------FSED---ADSISVHGV

***Culex quinquefasciatus***  MQKGEGTSCYYRFLTKGQQWIWLQTRFYITYHQWN---SKPEFVVCTHRVVSY---ADVMKQFRN--QTGGENK----------FAEDPARADSVSVG-V

***Aedes aegypti***  MQKGEGTSCFYRFLTKGQQWIWLQTRFYITYHQWNPARSKPEFVVCTHRVVSY---ADVMKQMRN--QAGGEGK----------FSED---TDSVSIG-V

***Drosophila melanogaster*** RQTGEGKSCYYRFLTKGQQWIWLQTDYYVSYHQFNPARSKPDYVVCTHKVVSY---AEVLKDSRKEGQKSGNSNSITNNGSSKVIASTGTSSKSASATTT

**510 520 530 540 550 560 570 580 590 600**

**....|....|....|....|....|....|....|....|....|....|....|....|....|....|....|....|....|....|....|....|**

***Anopheles darlingi***  ERKFQQSS------SQSLLATSPWSSKSSRTSR---VAPTPGVSPTGLSN---RGRNRYNTYNGP------GSDSATSIS---TESHTSRQSLVTPARQH

***Anopheles gambiae***  ERKFQQSS------SQSLLATSPWSSKSSRTSR---IAPTPGVSPTGLSNPARRGRHRYNTYHGP------GSDSATSIS---TESHTSRQSLVT---QH

***Culex quinquefasciatus***  ERKFQPGS------SQSLLATSPWSSKSSRTSR---IAPTPGVSPTGTSS---RGRHRYNTYQGP------GSDSATSISPARAESHASRQSMMT--THS

***Aedes aegypti***  ERKFQPSS------SQSLLATSPWSSKSSRTSRPARIAPTPGGSPTGAPS---RGRHRYNTYQGP------GSDSATSMS---AESHVSRQSMMT--QHS

***Drosophila melanogaster*** LRDFELSSQNLPARDSTLLGNSLASLGTETAATSPAVDSSPMWSASAVQP---SGSCQINPLKTSRPASSYGNISSTGISPKAKRKCPARYFYNNRGNDS

**610 620 630 640 650 660 670 680 690 700**

**....|....|....|....|....|....|....|....|....|....|....|....|....|....|....|....|....|....|....|....|**

***Anopheles darlingi***  SRSRTRTSTFPSKVSSQSQGSQQDRQPVSHFLLQQQQQQHQQQQQQQQQQAFQQQQQQQQPPPPELQQSLQQSQHHQLQLQQNQLQQQLQQQQQQHRTPA

***Anopheles gambiae***  SRSRMRTSTFPSKVS--SHGSQSDRNSMSHFLLQQPQPARQSHGGHQLPSGQLQHQQQHLPQQQQHQQQQQQQPQHPLQHQQPHPLLQPHQQLQGHQAQQ

***Culex quinquefasciatus***  SKSRMRFNSYQSKTPSQH-SQDGGQPPATHFILQQ----------------------QMLPNP---QQTVQTESYQQMQLR---PARPP-----------

***Aedes aegypti***  --SVIRSNTFHSKPSSQDGSSQPARHTAAHFILQQ----------------------QMMPNP---QQPSP-ESFQQMLATTQHQIRPP-----------

***Drosophila melanogaster*** DSTSMSTDSVTSRQSMMTHVSSQSQRQRSHHREHHRENHHN---------QSHHHMQQQQQHQSQQQQHPARQQHQQLQQQLQHTVGTPKMVPLLPIAST

**710 720 730 740 750 760 770 780 790 800**

**....|....|....|....|....|....|....|....|....|....|....|....|....|....|....|....|....|....|....|....|**

***Anopheles darlingi***  RQHQQLQQLQAHQQHQPAPQQQPQQAQLMVQTLSEPEMYGSKMAMVNQSS----AHQGLAHNQHDTSQQHQQQQPMDQQQQQQQQQQQHQQLIQQTT---

***Anopheles gambiae***  PHQQLPPQQQPARPHPAMIQNPPDVYPHQLQTQHPLQQQQQQQQQSQQPSQQQQSHQQLAGLQQPGTVQQQQSHQQPQHQQPQPARQQQPPLPQPSTTAA

***Culex quinquefasciatus***  ----------------------------------------------------------------------------------------------------

***Aedes aegypti***  ----------------------------------------------------------------------------------------------------

***Drosophila melanogaster*** QIMAG-----------------------------------------------------------------------------------------------

**810 820 830 840 850 860 870 880 890 900**

**....|....|....|....|....|....|....|....|....|....|....|....|....|....|....|....|....|....|....|....|**

***Anopheles darlingi***  ---APATILTPPARTVTQIIGAPGFIEPQQYLTAIPVQ---PVASFTADGASGVLSPISPTSP---HGAY----HPAVTASGGVVLTPTQNQVQDQLQRK

***Anopheles gambiae***  LRPPPATIITPP---VTQILTATGFIEPQQYLTAIPVQ---SVAGFTET-APGVLSPIQSTSPPARHGAYAVAHHPAVTGTGGVVLTPSQNQVQDQLQRK

***Culex quinquefasciatus***  ---VTAAIITPPS--VTQIIGSAGFMEPQQCLAAIPVQ---PVAAFAPDASAAVLSPIPPTSP--ATFAVHPSVSSAAPARGGVVLTPTQNQVQDQLHRK

***Aedes aegypti***  ---PAAAIITPP---VTQIISPAGFIEPQQYLAAIPVQPARPVAGFPPDSSTGVLSPIPPTSP--AAYAVHPTVPSAA---GSVVLTPAQNQVQDQLQRK

***Drosophila melanogaster*** ---NACQFPQPAYPIASPQLVAPTFLEPPQYLTAIPMQP--VPARIAPFPVAPVLSPLPVQSQ-------------TDMLPDTVVMTPTQSQLQDQLQRK

**910 920 930 940 950 960 970 980 990 1000**

**....|....|....|....|....|....|....|....|....|....|....|....|....|....|....|....|....|....|....|....|**

***Anopheles darlingi***  HEELQHLILQQQEELR---RVQEQLLMPARARYGLLPSIVSLPFPAAAP-AATPANAGPSVVPQ-DRGSNGSSFMQHHPAAGHGSSFHHHHHHYHPSSSG

***Anopheles gambiae***  HEELQHLILQQQEELR---RVQEQLLM---ARYGLLPSIVSLPARPFPPGTGNGPGPGPSVGPPGERCASGGSFLQAHHQGNS-----YHHHHYHPSAAQ

***Culex quinquefasciatus***  HEELQHLIMQQQDELR---RVQEQLLM---ARYGLLPSIVTIPFTSASS-STNDINSRP-------ARCSSSAYLQHHQSPHS-----SHQYQTHASLMG

***Aedes aegypti***  HEELQHLIMHQQEELRPARRVQEQLLM---ARYGLLPSIVTLPFTSASS-STIDISSR---------CSSSTSYLQHHQSSHS-----SHQYPTHPSLAN

***Drosophila melanogaster*** HDELQKLILQQQNELR---IVSEQLLLSRYTPARYLQPMMSMGFAPGNMTAAAVGNLGASGQRGLNFTGSNAVQPQFNQYGFALNSEQMLNQQDQQMMMQ

**1010 1020 1030 1040 1050 1060 1070 1080 1090 1100**

**....|....|....|....|....|....|....|....|....|....|....|....|....|....|....|....|....|....|....|....|**

***Anopheles darlingi***  SSS-------VQSHHTQSSHQHHSHPQDHQHQHAMQQQQPARQQSQQQMCIPPPDQKPILHPDSEQLNFTDTGQGK-LAETG----EMVSYMQLTPVPLH

***Anopheles gambiae***  AQ---------QQQQQQQQQHPPSHGGVHPARHAQQPQHPLQD--QQQLCIGPPDQKPILHPDPEQLNFTDTGQGK-LGEPN----EMISYMQLTPVPLH

***Culex quinquefasciatus***  ---------------------------------NNPQQQPLPQ-----LCIPP-DQKPIFHPN-QQLNFTDTGQLRQPGDPTGPAREGTSYMQLTPVPIH

***Aedes aegypti***  QP--------------------QNLLNPARPQPHQSQQQPDQQ-----MSIPL-DQKPIFHPN-QQLNFTDTGQPKLPGDPN---SEAISYMQLTPVPIH

***Drosophila melanogaster*** QQQNPARLHTQHQHNLQQQHQSHSQLQQHTQQQHQQQQQQQQQQQQQQQQQQQQQQQQQQQQQQQQQLQLQQQNDILPARLREDIDDIDAFLNLS--PLH

**1110 1120 1130 1140 1150 1160 1170 1180 1190 1200**

**....|....|....|....|....|....|....|....|....|....|....|....|....|....|....|....|....|....|....|....|**

***Anopheles darlingi***  HLQQQQQQQQQQQQQQQQQQQQQQQHHQHHPASASNSTVTPAPSVLNPARQQQQQMQQQQQLMQLGIPADGTAGPGAPGANSSNG--MGLLQYQMVQEQA

***Anopheles gambiae***  HLQHQSSQS---------------------PARSTSSTPATSMMSQQQKQHQQQQHQQQQQHMQMGAPPDGGTGAGGNSNNNSNGNTIGLLQYQMAAEQA

***Culex quinquefasciatus***  HLQQH----------------------------------------PQPSQQPQQQQQQQQ--------LPG-TGNS-----------MELLQYQMASEQA

***Aedes aegypti***  HLQQQ----------------------------------------SQPLQPARQQQQHHHQMQNPGMNLPGPSGNS-----------MELLQYQMAEEQA

***Drosophila melanogaster*** SLGSQSTIN-----------------PFNSSSNNNNQSYNGGSNLNNGNQNNNNRSSNPPQNNNEDSLLPARSYMQMATESSPSINFHMGISDDGSETQS

**1210 1220 1230 1240 1250 1260 1270 1280 1290 1300**

**....|....|....|....|....|....|....|....|....|....|....|....|....|....|....|....|....|....|....|....|**

***Anopheles darlingi***  QTL---FTSG---MEQQQCQP------------------QQQQQSHPPSQPSTIQQQQQQQHQQGAS-----------------SSDAGSRTCPARPSQT

***Anopheles gambiae***  QILPARFTSG---MEQQQGQQ------------------QQQPAVQQQ------QQQQQQQQHQGAG-----------------SSDAGSRTCPS---QT

***Culex quinquefasciatus***  HVL---FTSG---MEQQQQQQ------------------QPARQQQQQ---IHPLHHHHHHHQQQQ------------------GSSLHSPGGST-SHSR

***Aedes aegypti***  QTL---FTSG---MEQQQQQQQHLPQPQQQHHHHQQQQQQPARQMHHQHSHHHQHHHQHHHHQQQQQQQQQQPQHHQSQHHHSQSSSVGSPGGSTRSHPR

***Drosophila melanogaster*** EDNKMMHTSGSNLVQQQQQQQQ------------QQQILQQHQQQSNSFFSSNPPARFLNSQNQNQNQLPNDLEILPYQMSQEQSQNLFNSPHTAPGSSQ

**1310 1320**

**....|....|....|....|**

***Anopheles darlingi***  SELPARPAR-----------

***Anopheles gambiae***  SEMPARPAR-----------

***Culex quinquefasciatus***  SDVPARPAR-----------

***Aedes aegypti***  SDMPARPAR-----------

***Drosophila melanogaster*** PARPARPARTIMELESSPAR

CYCLE

**10 20 30 40 50 60 70 80 90 100**

**....|....|....|....|....|....|....|....|....|....|....|....|....|....|....|....|....|....|....|....|**

***Anopheles darlingi***  MVARNFTLQDMPYNRALQVQPDEEREFLTLDELKPHQLTELGVTVSSVAMPSVNSHHPTAQPQHRVSGPNAADTAPNHPAITTAGGLMQNYHHHHNVFYE

***Anopheles gambiae***  ----------------------------------------------------------------------------------------------------

***Culex quinquefasciatus***  MVARNFSLQDLPYN-NLLQVPEEEREFLTLEELKPHQLTELGVSIVTSPGSNTLVTSIGGGGGTTMVASDIPARTSHSSAASTAGNEHLTATTASSVVSL

***Aedes aegypti***  ----------------------------------------------------------------------------------------------------

***Drosophila melanogaster*** ----------------------------------------------------------------------------------------------------

**110 120 130 140 150 160 170 180 190 200**

**....|....|....|....|....|....|....|....|....|....|....|....|....|....|....|....|....|....|....|....|**

***Anopheles darlingi***  PARLGATHSPAANSTAAVPGDHMTGPDASLLVDPSLAGTTMSAVTNLSSLSAAAAAAAAAAAAAATGSGNHHHHHHLHADNPAGHQHHNNSHHHQTTGSG

***Anopheles gambiae***  -----------------------------MLTSP----ILICFFPLLFSL--------------------------------------------------

***Culex quinquefasciatus***  DPMQGSSSVVGTTAVAHQPGDQHHYHQHHHQHHHHQLHSQHTDTPARDGGGSE-----------------------------------------------

***Aedes aegypti***  ----------------------------------------------------------------------------------------------------

***Drosophila melanogaster*** ----------------------------------------------------------------------------------------------------

**210 220 230 240 250 260 270 280 290 300**

**....|....|....|....|....|....|....|....|....|....|....|....|....|....|....|....|....|....|....|....|**

***Anopheles darlingi***  GGGPARGGGGGGGSRKRKFSFNSHFSDTSDVEDDTCDDSKSVRTADESKKQNHSEIEKRRRDKMNTYITEL---SAMIPMCHAMSRKLDKLT---VLRMA

***Anopheles gambiae***  ------------------FISSPYCSDTSDIEDDTCDDSKSVRTADESKKQNHSEIEKRRRDKMNTYITELPARSAMIPMCHAMSRKLDKLT---VLRMA

***Culex quinquefasciatus***  ------NDGGGGGSRKRKFSYN----DNSDIEDDTGDDAKSVRTTDDNKKQNHSEIEKRRRDKMNTYITELSAMPARIPMCHAMSRKLDKLT---VLRMA

***Aedes aegypti***  -------------------LLISVFSDNSDIEDDTGDDAKSVRTTDENKKQNHSEIEKRRRDKMNTYITEL---SAMIPMCHAMSRKLDKLTPARVLRMA

***Drosophila melanogaster*** ------------------MEVQEFCENMEEIEDENYDEEKSARTSDENRKQNHSEIEKRRRDKMNTYINEL---SSMIPMCFAMQRKLDKLPARTVLRMA

**310 320 330 340 350 360 370 380 390 400**

**....|....|....|....|....|....|....|....|....|....|....|....|....|....|....|....|....|....|....|....|**

***Anopheles darlingi***  VQHLKTIRGAVHPARSYTEGHYKPAFLSDQELKMLILQAAEGFLFVVGCD---RGRILYVSESVSHVLNYS---QGDLLGQSWFDILHPKDVAKVKEQLS

***Anopheles gambiae***  VQHLKTIRGAVH---SYTEGHYKPAFLSDQELKMLILQAAEGFLFVVGCDPARRGRILYVSESVSHILNYS---QGDLLGQSWFDILHPKDVAKVKEQLS

***Culex quinquefasciatus***  VQHLKTIRGAVH---SYTEGHYKPAFLSDQELKMLILQAAEGFLFVVGCDRGRPARILYVSESVSQILNYS---QGDLLGQSWFDILHPKDVAKVKEQLS

***Aedes aegypti***  VQHLKTIRGAVH---SYTEGHYKPAFLSDQELKMLILQAAEGFLFVVGCD---RGRILYVSESVSQILNYSPARQGDLLGQSWFDILHPKDVAKVKEQLS

***Drosophila melanogaster*** VQHLRGIRGSGS-LHPFNGSDYRPSFLSDQELKMIILQASEGFLFVVGCD---RGRILYVSDSVSSVLPARNSTQADLLGQSWFDVLHPKDIGKVKEQLS

**410 420 430 440 450 460 470 480 490 500**

**....|....|....|....|....|....|....|....|....|....|....|....|....|....|....|....|....|....|....|....|**

***Anopheles darlingi***  SSDLSPRERLIDAKTMLPVKTPARDVPQGVTRLCPGARRSFFCR---MKC---KANVQIKEEADATSSTTNSATVCHRRKKQVNSDKKYSVIQCTGYLKS

***Anopheles gambiae***  SSDLSPRERLIDAKTMLPVKTDVPQGPARVTRLCPGARRSFFCR---MKC---KANVQVKEEADQPNSVSSVNNVCHRRKKQVNSDKKYSVIQCTGYLKS

***Culex quinquefasciatus***  SSDLSPRERLIDAKTMLPVKTDVPQGVTRPARLCPGARRSFFCR---MKC---KTNVQVKEEAESGNTSSSS---CHRRKGKVNSDKKYSVIQCTGYLKS

***Aedes aegypti***  SSDLSPRERLIDAKTMLPVKT---DVPQGVTRLCPGARRSFFCR---MKCPARKTNIQVKEEAESNGSTSS----CHRRKNKVNSDKKYSVIQCTGYLKS

***Drosophila melanogaster*** SLEQCPRERLIDAKTMLPVKT---DVPQSLCRLCPGARRSFFCRPARMKLR-TASNNQIKEESDTSSSSRSS----TKRKSRLTTGHKYRVIQCTGYLKS

**510 520 530 540 550 560 570 580 590 600**

**....|....|....|....|....|....|....|....|....|....|....|....|....|....|....|....|....|....|....|....|**

***Anopheles darlingi***  WAPAK---IGLEEN---ETDGEGDSCNLSCLVAVGRPARIQPNLHQPPQHGTAMQRPSDAGTGNGSNGGGPPTNGGGGGGPNSNGGNTGSAAAAGGGGGG

***Anopheles gambiae***  WAPAKPARIGLEEN---ETDGEGDSCNLSCLVAVG---RVQPNLSQS-----------------------------------------------------

***Culex quinquefasciatus***  WAPAK---IGLEEHPARETDGEGDSCNLSCLVAVG---RVQPSLFQPPIAPSGTNLSNQRPTMEG--------------------------------AGA

***Aedes aegypti***  WAPAK---IGLEEH---ETDGEGESCNLSCLVAPARVGRNQISGNGN-----------------------------------------------------

***Drosophila melanogaster*** WTPIK-----DEDQ---DADSDEQTTNLSCPARLVAIGRIPPN---------------------------------------------------------

**610 620 630 640 650 660 670 680 690 700**

**....|....|....|....|....|....|....|....|....|....|....|....|....|....|....|....|....|....|....|....|**

***Anopheles darlingi***  AGGSNGTAGKSAVSRNSIPNLR---NVQFISRHAMDGKFLFVPARDQRATLVLGFLPQELLGTSMYEYY---HHEDIPALAESHKAALQGTQCVTTSVYR

***Anopheles gambiae***  CSLSNGKQ----LNRNTIPNLR---NVQFISRHAMDGKFLFVDQPARRATLVLGFLPQELLGTSMYEYY---HHEDIPALAESHKAALQGTQCVTTSVYR

***Culex quinquefasciatus***  GTDRSGTNGCKGTSRNNIPNLRPARNVQFISRHAMDGKFLFVDQ---RATLVLGFLPQELLGTSMYEYY---HHEDIPALAESHKVALQGNQCVTTPVYR

***Aedes aegypti***  --LTNRRVTG--FNRNNIPNLR---NVQFISRHAMDGKFLFVDQ---RATLVLGFLPQELLGTSMYEYYPARHHEDIPALAESHKAALQGNQCVTTPVYR

***Drosophila melanogaster*** --VRNSTVP---ASLDNHPNIR---HVLFISRHSGEGKFLFIDQ---RATLVIGFLPQEILGTSFYEYFHNPAREDIAALMESHKMVMQVPEKVTTQVYR

**710 720 730 740 750 760 770 780 790 800**

**....|....|....|....|....|....|....|....|....|....|....|....|....|....|....|....|....|....|....|....|**

***Anopheles darlingi***  L---RTKESGFVRLQSEWKSFRN---PWTKDIEYLIAKNNVILSELLLGGDVNAPARNRNGSGYGVGSDGLGDGTG---DGTGVSGSTGQPNVGYEFFNH

***Anopheles gambiae***  L---RTKETGFVRLQSEWKSFRNPARPWTKEIEYLIAKNNVILAELGDGGTARA-------GGYGMG--ELGDGTG---EPG--SGAPGQPGVGYEFFNH

***Culex quinquefasciatus***  LPARRIKESGFVRLQSEWKSFRN---PWTKEIEYLIAKNNVILSELVESG-----------------------------------GPSGRTMAGGATVHG

***Aedes aegypti***  L---RTKENGFVRLQSEWKSFRN---PWTKEIEYLIAKNNVILVDLAEPARGTGGSYGGTSANVQSRCNGNGTTTGNEISDGNNNESNGQSTVGYEIFNH

***Drosophila melanogaster*** F---RCKDNSYIQLQSEWRAFKN---PWTSEIDYIIAKNSVFLPAR------------------------------------------------------

**810 820 830 840 850 860 870 880 890 900**

**....|....|....|....|....|....|....|....|....|....|....|....|....|....|....|....|....|....|....|....|**

***Anopheles darlingi***  SNGREIQRMI---NSHVEASKIG---RQIAEQVLDHQRRLGDSSSESSPNPNEA-AMQPAFSSALSEPARTNHSNDASTSGEHSMATSSG---VSPSSVS

***Anopheles gambiae***  TNGREIQRMIPARNSHVEASKIG---RQIAEQVLDHQRRVGDSSSESSPNPNEP-TLQPAFSSALSE---ANHSNDAITSGDHSMVTSTGPARVSPSSMA

***Culex quinquefasciatus***  SNG----------NGGGGGSSSP---ARNSTELSGGDGNNESNGQKSSPDPAEPPGLPANFTPAIIQEA-SNLSSEAIAAVERTLALSSGVSPASSSGGP

***Aedes aegypti***  SNGREIQRMI---NSHVEASKIGRPARQIAEQVLDHQRRIGDSSSESSPDPTDS-AIAQSFNPAIQEAN--NLSSEAIAAVERSLASSSGVSPASSSGAG

***Drosophila melanogaster*** ----------------------------------------------------------------------------------------------------

**910 920 930 940 950 960 970 980 990 1000**

**....|....|....|....|....|....|....|....|....|....|....|....|....|....|....|....|....|....|....|....|**

***Anopheles darlingi***  VVPSS------------------------VVTTRINGTLPGYSHVQTN--AIISPEHDVSQAQAN--------STDGNDEAAM---AVIMSLLEADAGLG

***Anopheles gambiae***  VVPP-------------------------TVTTRINGTLPGYSHVQTN--AIISPEHDVSQTQAS--------STDGNDEAAM---AVIMSLLEADAGLG

***Culex quinquefasciatus***  ARGAP------------------------GTPQRVNGTLPGYNHVRNNSNAIMSPEHEVPHVQVNNSSAAAAAAADGNDEAAM---AVIMSLLEADAGLP

***Aedes aegypti***  PARATGGANGSGGAGTASTVGPNGGQATGPPPQRINGTLPGYNHVRNN--EIVSPEHEVPHSQAN--------STDGNDEAAMPARAVIMSLLEADAGLG

***Drosophila melanogaster*** ----------------------------------------------------------------------------------------------------

**1010 1020**

**....|....|....|....|..**

***Anopheles darlingi***  G---PVDFSGLPWPARPLPPAR

***Anopheles gambiae***  GPARPVDFSGLPWPLPPARPAR

***Culex quinquefasciatus***  ARGGPVDFSGLPWPLPPARPAR

***Aedes aegypti***  G---PVDFSGLPWPLPPARPAR

***Drosophila melanogaster*** ----------------------

PERIOD

**10 20 30 40 50 60 70 80 90 100**

**....|....|....|....|....|....|....|....|....|....|....|....|....|....|....|....|....|....|....|....|**

***Anopheles darlingi***  MSTTGMETMEGAESTHNTKVSDSAYSNSCSNSQSQRSGSSKSRHSGSNSSGSSGYGGKASTQASSI---PPVPQPAIKRTKDKDRKKKKLKTSTDG----

***Anophelesgambiae***  MSTTGMENLEGADSTHNTKVSDSAYSNSCSNSQSQRSGSSKSRHSGSNSSGSSGYGGKASTQASSIAPLPPARQAAAKRTKDKERKKKKLKTSTDSGTVP

***Aedes aegypti***  -------------------------------------GSSKSRHSGSNSSGSSGYGGKGNIQAG---------------TDDKDRKKKKLKTSVEP----

***Culex quinquefasciatus***  --------------------------MTVLSKFGQNRGSSKSRHSNSSSGGSSGYGGKNNTPGAIPG--PITQHPVIKRTKDKDRKKKRIRTSIEA----

***Drosophila melanogaster*** -----MEGGESTESTHNTKVSDSAYSNSCSNSQSQRSGSSKSRLSGSHSSGSSGYGGKPSTQASSSD-------MIIKRNKDPARKSRKKKKNKGAGQG-

**110 120 130 140 150 160 170 180 190 200**

**....|....|....|....|....|....|....|....|....|....|....|....|....|....|....|....|....|....|....|....|**

***Anopheles darlingi***  NTNATATPARTSVTVGGVAGATTTPSTGSTLSNNGGGDQG--CNFAEGDHTTATVVSGITGSAGGSASGPTSSSNNTELCSGSGATGNDDIGDPNGSGEV

***Anopheles gambiae***  APNGSAGPGGVTAGTAPNAAGTVANANAVSDVEQQTLAAGGCGPAREPEPMETTTGNSSVASVSGELGAVGAGAGGMQSASHGGESNEHHELGPITGGNG

***Aedes aegypti***  TPGGTGVAGEEPFSTGATAGGNFTTGPARS-----------------GEQVQSSVHASTVG--QGEAQQNQSHVDDTDVPGGDEPTAK---GKPFGRGES

***Culex quinquefasciatus***  SGPARPGTATAACGNGANGGTGSTETDQLMACDGAGPGSA--SGQGETGASTGVHQEMNAQPEMNEASKPNGSPATAPARTCDEPTLASDENQTMHSQPQ

***Drosophila melanogaster*** AGQAQTLISASTSLEGRDEEKPRPSGTGCVEQQICRELQDQQHGEDHSEPQAIEQLPARQQEEEEDQSGSESEADRVEGVAKSEAAQSFPIPSPLSVTIV

**210 220 230 240 250 260 270 280 290 300**

**....|....|....|....|....|....|....|....|....|....|....|....|....|....|....|....|....|....|....|....|**

***Anopheles darlingi***  NEITGHGTNQECPARSSDADQITARPSDEEKAKENGMQTSKTGQSRADRFELQKKLAHSVLPGPAPLPAGVASIVRSITAGDDGLGNCCGAGVRAGATDS

***Anopheles gambiae***  TAELVSVEVAAAAAVAPARQIINEENTKDDRKHLSQEQLHRAGGGGTHDGRQKSLVAQSILPGPAPLPAGVASIVNSIATG-------------------

***Aedes aegypti***  -----------------NGNRVPATPANAKTETEN-----------------------------------------------------------------

***Culex quinquefasciatus***  ----AESANVIDAAMPSSSGVVPMEEGGVEGEGVEGAEGAPKTPADCN----------------------------------------------------

***Drosophila melanogaster*** PPSMGGCGGVGHAAGLDSGLAKFDKTWEAPARGPGKLESMTGVGAAAAG---------------------------------------------------

**310 320 330 340 350 360 370 380 390 400**

**....|....|....|....|....|....|....|....|....|....|....|....|....|....|....|....|....|....|....|....|**

***Anopheles darlingi***  MQTGKRASTELPGAGPARLLMREALGEQLKQQESSHHQSHQSAQQQHTTLQHHPTVQVQDTAQDNHHHHHHHHHHHHQQQRQGQGTHLQPPSLQLHPPQQ

***Anopheles gambiae***  -------AGLRTGTVPARSSSGTLLGVVQQQQQQQQHQQQQQQQHHGG----LNQCQQKQTQHHHHHHGAHQHPKQQHSSMQDPSLANQKHVPARPQQHS

***Aedes aegypti***  ------------GFCPAR----------------------------------------------------------------------------------

***Culex quinquefasciatus***  ---------PMKAAKPAR----------------------------------------------------------------------------------

***Drosophila melanogaster*** -----------TGQRGER----------------------------------------------------------------------------------

**410 420 430 440 450 460 470 480 490 500**

**....|....|....|....|....|....|....|....|....|....|....|....|....|....|....|....|....|....|....|....|**

***Anopheles darlingi***  QQQQQQQQQQAQQQQQQQPARQQQQQQQQQQQQQQQQQQQSNTPRTNKPDVEDGFCCVISMHDGV---VLFTTPSITHSLGFPKDMWLGRSFIDFVHP--

***Anopheles gambiae***  AQQPHHQQQQPLDKQHLQPQQQPQQQQQQQQQQQQQAQPQSNTPRTNKPDVEDGFCCVISMHDGVPARVLFTTPSITHSLGFPKDMWLGRSFIDFVHP--

***Aedes aegypti***  --------------------------------------------------------CVISMHDGV---VLFTTPSITSSLGFPNDMWLGRSFIDFVHP--

***Culex quinquefasciatus***  ------------------------------------------------PEVEDGFCCVISMLDGV---VLFTTPSITRSLGFPKDMWLGRSFIDFVHP--

***Drosophila melanogaster*** -------------------------------------------------VKEDSFCCVISMHDGI---VLYTTPSITDVLGYPRDMWLGRSFIDFVHLPA

**510 520 530 540 550 560 570 580 590 600**

**....|....|....|....|....|....|....|....|....|....|....|....|....|....|....|....|....|....|....|....|**

***Anopheles darlingi***  -KDRATFASQITSKVVVPLGESKS--GQKPARDQKNSLYVMLRKYR---GLKSAGFGVTKTTVNYEPYRLVLTFREAPVENT----DLVS---GRSILLI

***Anopheles gambiae***  -KDRATFASQITSKVVVPLGESKS-----GQKDQKNSLYVMLRKYRPARGLKTAGFGVTKTTVNYEPYRLVLTFREAPAENA----EIMS---GRSILLI

***Aedes aegypti***  -KDRSTFASQITSKVVVPLGESKNGVGHKDQKPARNSLYVMLRKYR---GLKSAGFGVTGTNVNYEPYRLVLTFREAPNDTS----EDIKNT-GRNILLI

***Culex quinquefasciatus***  -KDRATFASQITSKVVLPLGESKNPARGSNNKDQKNCLYVMLRKYR---GLKSAGFGVTKTTVNYEPYCLVLTFREAPNDNS----GEVSPITGRNILLI

***Drosophila melanogaster*** RKDRATFASQITTG--IPIAESRG----SVPKDAKSTFCVMLRRYR---GLKSGGFGVIGRPVSYEPFRLGLTFREAPEEPARARPDNYMVSNGTNMLLV

**610 620 630 640 650 660 670 680 690 700**

**....|....|....|....|....|....|....|....|....|....|....|....|....|....|....|....|....|....|....|....|**

***Anopheles darlingi***  ISATPVKSIYREP---NESLCERELKFST---RHTTSGVLSYVDGNSVPARESIGYLPQDIL---GRSIMELYHPDDMPILRKTYE---TVMVKGQTA--

***Anopheles gambiae***  ISATPVKSVYKEP---NESLCERDLKFSTPARRHTTSGVLSYVDGNSV---ESIGYLPQDIL---GRSVMELYHPDDMPILRKAYE---TVMVKGQTA--

***Aedes aegypti***  ISATPVKSVYTVSPARNEQLHDKELKFST---RHSTNGVLNYVDGNSV---ESIGYLPQDIL---GRSIMELYHPEDLPSLKNIYE---TVMIKGQTAPA

***Culex quinquefasciatus***  VSATPARPVKSVYTQPNERMNERKLRFST---RHTTNGVLNYVDGNSV---ESIGYLPQDIL---GQSIMELYHPEDMPILKKMYEPARTIMVKGRTA--

***Drosophila melanogaster*** ICATPIKSSYKVP---DEILSQKSPKFAI---RHTATGIISHVDSAAVS---ALGYLPQDLIPARGRSIMDFYHHEDLSVMKETYE---TVMKKGQTA--

**710 720 730 740 750 760 770 780 790 800**

**....|....|....|....|....|....|....|....|....|....|....|....|....|....|....|....|....|....|....|....|**

***Anopheles darlingi***  -GASFVGQPYRFLV---NNGCYIVLSTEWTSFVNPWSRELEFVI---GSHRILQGPSTADVFASGFPARYCQNQNQFPD---EVLKEAKMIEEQI---LR

***Anopheles gambiae***  -GASFVSQPYRFLVPARNNGCYIVLSTEWTSFVNPWSRELEFVI---GHHRILQGPSIADVFAVSP--FYCQTQNQFSD---DTLKEAKMVEEQIPARLR

***Aedes aegypti***  RGASFVSQPYRFLV---NNGCYIVLKTEWASFVNPWSRELEFVI---GNHYIQQGPSNPNVFASKF---YCKDPLLFPDDPARLLKEAKMIEEQI---LR

***Culex quinquefasciatus***  -GASFN-------------GCYLVLKTEWTSFVNPWSRELEFVI---GNHHIMEGPSDPDVFALHFN-----HQHQFADELLKPAREAKTIEEQI---LR

***Drosophila melanogaster*** -GASFCSKPYRFLI---QNGCYVLLETEWTSFVNPWSRKLEFVVPARGHHRVFQGPKQCNVFEAAP-----TCKLKISE---EAQSRNTRIKEDI---VK

**810 820 830 840 850 860 870 880 890 900**

**....|....|....|....|....|....|....|....|....|....|....|....|....|....|....|....|....|....|....|....|**

***Anopheles darlingi***  ILKEPVAKPSDMVKQEVSKRCKALASFM---EELMDEVAQPELKLNLLNESDFTFSERD---SVMLGEISPHHE---YFDSKSSPARSETPPSYNQLNYN

***Anopheles gambiae***  LLKEPVAKPSDMVKQEVSKRCKALASFM---EELMDEVAQPELKLNLLNESDFTFSERD---SVMLGEISPHHEPARYFDSKSS---SETPPSYNQLNYN

***Aedes aegypti***  LLKEPVAKPSDMVKQEVTKRCKALASFM---EELMDEVAQPELKLNLLNESDFTFSERDPARSVMLGEISPHHE---YFDSKSS---SETPPSYNQLNYN

***Culex quinquefasciatus***  LLKEPVTKPSDTVKQEVSKRCKALASFM---EELMDEVTQPELKLNLLNESDFTFSERDSVMPARLGEISPHHD---YFNSKSS---SETPPSYNQLNYN

***Drosophila melanogaster*** RLAETVSRPSDTVKQEVSRRCQALASFMPARETLMDEVSRADLKLELPHENELTVSERD---SVMLGEISPHHD---YYDSKSS---TETPPSYNQLNYN

**910 920 930 940 950 960 970 980 990 1000**

**....|....|....|....|....|....|....|....|....|....|....|....|....|....|....|....|....|....|....|....|**

***Anopheles darlingi***  ENLQRFFDSRPAMNIEEPLKMDSSGGTNTETIDEQT---HVSPNQR----FSASGGGGSGGSAGNFSSESNAQMDSTTNT-TSNTGGTG-TGTSSGPARG

***Anopheles gambiae***  ENLQRFFDSRPAMNIEEQMKMDSSGGTNTETIGDEQQSHAVSPNQRGFSAPARSGGGGSGGSAGNFSSESNAQMDSTTNT-TSNTGGTGGTGTSSG--GG

***Aedes aegypti***  DNLQRFFESRPVMNVKESSKIHSSGGTNTETIDDQ----RFSPAR--------GDGGESGGSAGNFSSESNVQMDSVTNT-TSNTG------TSSG----

***Culex quinquefasciatus***  DNLQRFFDSRPVMNIEESLKNDSSGGTNTEIQDDRT---NVSPNPARQ-RFSASGGGGSGGSAGNFSSESNAQMDSTTNT-TSNTG------TSSG----

***Drosophila melanogaster*** ENLLRFFNSKPARPVTAPAELDPPKTEPPEPRGTCVSG-ASGPMSP------VHEGSGGSGSSGNFTTASNIHMSSVTNTSIAGTGGTGTPARGTG--TG

**1010 1020 1030 1040 1050 1060 1070 1080 1090 1100**

**....|....|....|....|....|....|....|....|....|....|....|....|....|....|....|....|....|....|....|....|**

***Anopheles darlingi***  GSFQPPTLTEELLDKHNEDMQKVMLKKHREARMVARG---TEKNKKGPPDKG-YAAVPVPHGVKRGSSHSWEGDPHKTIKHQHNADPSATGTGGTGLQQQ

***Anopheles gambiae***  GSFQPPTLTEELLCKHNEDMQKVMLKPARKHREARMVARGTDKNKKGPPDKAGYGGGAVGHGVKRGSSHSWEGDAHKTIKHQHNPDGGGCS---AMLQQQ

***Aedes aegypti***  -SYQPPTLTEELLCKHNDDMQKVMLKKHREARSLAPARRVTDKNRKGPPDKT-YANI-IAHGVKRGSSHSWEGDIHKTFKHQHNPDNTCD----------

***Culex quinquefasciatus***  -SVQPPTLTEELLCQHNEDMQKVMLKRHREPARARTTSRGADKSRKGPPDKV-YTNI-ATHGVKRGSSHSWEEDMHKTAKHQHNSNPMRD----------

***Drosophila melanogaster*** TGTGTGTGTGTGTGTGTGTGTGTGTGNGTNSGTGTGTASSSKGGSAAIPPVTLTESLLNKHNDEMPAREKFMLKKHRESRGRTGEKSKKSAN--------

**1110 1120 1130 1140 1150 1160 1170 1180 1190 1200**

**....|....|....|....|....|....|....|....|....|....|....|....|....|....|....|....|....|....|....|....|**

***Anopheles darlingi***  QQQPARQHQKGPVASQALQQQLLIQQQQQQQQQQQQQQQQQQHHHQLQSQQPRQQLSAHGLLHPSSMHQHQSSIPILQQQQQQQQQQQQQQQLMPQLLQQ

***Anopheles gambiae***  QQPARQKMGPVAPHALQQQLLIQQQHQQLQQQQQQQQQQQQQQAQQQQQQLQQQQQQQQQQLYQQTNMHMHYKTQPARHVSHQLQYHHALQGQGSAALQQ

***Aedes aegypti***  --------------------------------------YQPQSSQALATPKPPQTSSAP-----------------------------------ARILDA

***Culex quinquefasciatus***  --------------------------------------YQPQSSSAQHTVLPKPARPTQQRLP-------------------------------VVPMDT

***Drosophila melanogaster*** -----------------------------------DTLKMLEYSGPGHGIKRGGSHSWEGEANKP-----------------------------KQQLTL

**1210 1220 1230 1240 1250 1260 1270 1280 1290 1300**

**....|....|....|....|....|....|....|....|....|....|....|....|....|....|....|....|....|....|....|....|**

***Anopheles darlingi***  SQS------------------------------------------QPTPARHSAGYLSESVAGSALHRLGATGFSTQAPSQLAY-----------SSLAG

***Anopheles gambiae***  TGGGMQTHLQQQQLLHHQQHQQQQQQQHQHLSHPAPSQQQQYPYQSPYPARHNNGPVQSTVAGESLLAGGSNQRSTFTTPSILAPQMAYGGAHTTGSSLT

***Aedes aegypti***  CTS------------------------------------------IVATTAVTATPLLSSVS---------------------------------NAFPM

***Culex quinquefasciatus***  CRA------------------------------------------VVTTTAVTMGQQTATVP-----------------------------------YNL

***Drosophila melanogaster*** GTD------------------------------------------AIKGAAGPARSAGGAVG---------------------------------TGGSS

**1310 1320 1330 1340 1350 1360 1370 1380 1390 1400**

**....|....|....|....|....|....|....|....|....|....|....|....|....|....|....|....|....|....|....|....|**

***Anopheles darlingi***  GRTGDLWPPFSVSVTSMQASA-----GIGTTSFVPSHSI---FPTLYYIPTAAAAAAAAAAAAAAATTPQPARPAAVAAAAAAAVAELPRLNATVPVPYM

***Anopheles gambiae***  GRTGDLWPPFSVSVTTMQASAPARGAGSSAASFVPSHSI---FPTLYYIP-----------------------------AAAAAATAAPQPAP-ITVPYM

***Aedes aegypti***  SRAVELCPPFSVSVTTIQATQ-----SNATTNIMPTSNI---FPTLYYIP---------------------ARPAPPQPTPASSALQIPRLNP-ITLPYM

***Culex quinquefasciatus***  PRAGEMWPPFSVSVTTVQTAQ-----TSSSASFVPSHSIPARFPTLYYIP-----------------------------AAAQPTSINPTLNP-ITVPYV

***Drosophila melanogaster*** FSSAGLFPTFYYIPASLTPTSP----TRSPRMHKHPHKGGTDMPTTSQQA------------------------------AAAAAQAMPLQYP----ARM

**1410 1420 1430 1440 1450 1460 1470 1480 1490 1500**

**....|....|....|....|....|....|....|....|....|....|....|....|....|....|....|....|....|....|....|....|**

***Anopheles darlingi***  AAGLMYPHPQLY-QQSMLYPPMMYHAMP---YQPLPPPCDTRNHQQQHQQQQQQQQ-----------------------QQQQQQQQQQQQQQQQQQQQP

***Anopheles gambiae***  AAGVMYPHPQLYHQQSLLYPPMMYHAMPARPYQPLPPPCGLDSDTRNVQIWSGSSLGTAAEHCAADDIVALALIFPLSKTHQHQQHQQQQQQQQQQQQQQ

***Aedes aegypti***  AG-VMYPHPQLY-QQSVLYPPMMYHAMP---YQPIPPPS---------------------------------------------------QLPSNQSGPA

***Culex quinquefasciatus***  AG-MMYPHPQLY-QQSVLYPPMMYHAMP---YQPAPPPCG-----------------------------------------------------LASDARN

***Drosophila melanogaster*** AG-VMYPHPSLF------YTHPAAAAATAMMYQPMPFPG------------------------------------------------------MANALQI

**1510 1520 1530 1540 1550 1560 1570 1580 1590 1600**

**....|....|....|....|....|....|....|....|....|....|....|....|....|....|....|....|....|....|....|....|**

***Anopheles darlingi***  PARQLQRQQRQTGQCDQQQTNGGCSVVVGTGGNAGVQSGPPGSGTLRSAAAGGGGGGGGGGGSNGAGAAGPQSRETTQQSVDGGGPGTNGGQDRGQHVVG

***Anopheles gambiae***  PARQQQQQQRQQQQQQQAQRLQS------------QPGHTTSTGGPGPAAPGGMAVGLGQPATGGQPSAQSAEVPGVPTNAGSGGP--------------

***Aedes aegypti***  PARRIYAQP---GFLD-------------------------GSGLYDTSNSGGN----------------KQQLQSIPSSIGGGG---------------

***Culex quinquefasciatus***  PARKLQPQPPTSQNPVPG-----------------------SSGQVVPGDMPGG----------------RQVLQTIPPPAGSGS---------------

***Drosophila melanogaster*** PERPLGSQS--------------------------------AYNKSVYTTTPAS---------------MTKKVPGAPARFHSVT---------------

**1610 1620 1630 1640 1650 1660 1670 1680 1690 1700**

**....|....|....|....|....|....|....|....|....|....|....|....|....|....|....|....|....|....|....|....|**

***Anopheles darlingi***  PCGPARSKRPARHTVAPSAGSGSLSQTPFQRPSSQATSVKAEPGSALGSIASASIVANRAFSESSKKDLTDSPVIS---NVDCADCALDEVLEKHTGSVA

***Anopheles gambiae***  ----ARGKHPAHMTVPPSAGSGSQSQTPFQRPSSQATSVKAEPGSALGSIASASIVVNRAFSESSKKDLTDSPLISPARNVDCTDCALDEVHDKHCDTTA

***Aedes aegypti***  -------------------GRGSQSQHLFQRPPSQATSVKTEPGSNMGSIAPA---------RSASIGLADSPRNS---HVEADFPQEDMEKIRSRGSKT

***Culex quinquefasciatus***  --------------------QSQVALTSFQWPPSQATSVKAEPGSNMP-------------------------------------------ARGSIASAS

***Drosophila melanogaster*** ------------------------TPAQVQRPSSQSASVKTEPGSSAAVSDP------------CKKEVPDSSPIP--------SVMGDYNSDPPCSSSN

**1710 1720 1730 1740 1750 1760 1770 1780 1790 1800**

**....|....|....|....|....|....|....|....|....|....|....|....|....|....|....|....|....|....|....|....|**

***Anopheles darlingi***  GGVRSGAGGPARGRDGTRGVSGTTNTGGGVGHRTGAPDKRPESGDHGTSLLGDRATVGLCEISDDMDESSFSSFYSSFLKTDNSSTEGGHNGTEKKDSSE

***Anopheles gambiae***  SGIDRRVNGAGTRRTGSGQVDKLPMGTGIGG---TAGNNGGDRDGGGGGGGGPARVVGLGEVSDDMDESSFSSFYSSFLKTDNSSE--GQNGAERKESSE

***Aedes aegypti***  PGP-------------------------------------------------------LWEISDDMDESSFSSFYSSFLKTDNSS---------------

***Culex quinquefasciatus***  -----------------------------------------------------------------MANRVNYSVQPGIRRRRRRA---------------

***Drosophila melanogaster*** PAN---------------------------------------------------------------NKVCYTPARNEVHWPARCYC--------------

**1810 1820 1830 1840 1850 1860 1870 1880 1890 1900**

**....|....|....|....|....|....|....|....|....|....|....|....|....|....|....|....|....|....|....|....|**

***Anopheles darlingi***  MCWESSSNNANTPARGHEPEDKNRGG--------CGRGGNSDGMVRPGTGDICRAKRRPNPPWLDNVCQTKDLIYRYQINERSLEELLDSDSLALKKISQ

***Anopheles gambiae***  MCWESGSNNATSTTNRMHVDGDGSGNQPARAARDRGGSGNGAGDGTGTLVRPSGAKRRPNPPWLDNVCQTKDLIYRYQINERSLKELLDSDNLALKKISQ

***Aedes aegypti***  ----------------------------------------------------------------------------------------------------

***Culex quinquefasciatus***  ----------------------------------------------------------------------------------------------------

***Drosophila melanogaster*** ----------------------------------------------------------------------------------------------------

**1910 1920 1930 1940 1950 1960 1970 1980**

**....|....|....|....|....|....|....|....|....|....|....|....|....|....|....|....|....|.**

***Anopheles darlingi***  PILVNDQLGQLYLDLELEGLSAKPARLSLSEATSGSSSDDCDTKDKTKVTKRNMKYSKLVMIYEENAPFPPPN---EPEPPARPAR

***Anopheles gambiae***  PARPILVNDQLGQLYLDLELEGLSAKLSLSEATSGSSSDDCDTKDKAKVNKRNMKYSKLVMIYEENAPFPPPNPARDSEPPARPAR

***Aedes aegypti***  ------------------------------------------------------------------------------ESPARPAR

***Culex quinquefasciatus***  -------------------------------------------------------------------------------IPARPAR

***Drosophila melanogaster*** ---------------------------------------------------------------------------------LEPAR

**Figure S-I2. Circadian cycle protein alignments among dipteran species.** Alignments of the *timeless, cycle, clock*, *timeout* and *period* gene products from *An. darlingi*, *An. gambiae*, *Cu. quinquefasciatus*, *Ae. aegypti* and *D. melanogaster* by ClustalW.
