# Supplementary material for: The Genome of Anopheles darlingi, the main neotropical malaria vector
Source: Nucleic Acids Res. 2013 Jun 12;41(15):7387–400. doi: 10.1093/nar/gkt484 (PMC3753621; doi:10.1093/nar/gkt484)
Supplement: Supplementary Data [file supp_gkt484_nar-00257-h-2013-File006_updated.zip › S-J.docx]

**S-J Insecticide resistance**

**Table S-J1. Summary of the putative detoxification genes in *Anopheles darlingi*.**

| **Classes** | **Gene ID** |
| --- | --- |
| **GSTs**  Delta | AD00801, AD07981, AD04770 |
| Epsilon | AD08200, AD08208, AD08204, AD08209, AD08205,AD08212 |
| Theta | AD00084, AD00085, AD00801, AD00835, AD08797 |
| Zeta | AD10694 |
| Unclassified | AD00804, AD00802, AD00803, AD00833, AD00834, AD01355, AD04261, AD04262, AD04639, AD07467, AD07726, AD08211, AD10633,AD0878, AD08859, AD08796 |
| **CCEs** |  |
| No classified | AD00316, AD00367, AD00377, AD01540, AD01782, AD02118, AD04963, AD05454, AD06814,AD7009, AD07716, AD07723, AD07929, AD07930, AD07931, AD08552, AD09160, AD10310, AD10364, AD10705 |
| **CYPs** |  |
| No classified | AD00130, AD00264, AD00387, AD00529, AD00552, AD00657, AD00697, AD00698, AD00699, AD00703, AD00734, AD00735, AD00796, AD01272, AD01504, AD01783, AD01784, AD01791, AD02234, AD02271, AD02544, D02549, AD02552, AD02555, AD02556, AD02557, AD02558, AD02559, AD02560, AD02561, AD02562, AD02563, AD02564, AD02565, AD02566,  AD02567, AD02713, AD02750, AD02753, AD02755,  AD02756, AD02905, AD02907, AD02977, AD03021,  AD03150, AD03151, AD03200, AD03214, AD03223, AD03254, AD03264, AD03280, AD03921, AD03922,  AD03923, AD03924, AD04229, AD04476, AD04477,  AD04503, AD04504, AD05602, AD05619, AD05623,  AD06406, AD06991, AD06992, AD07008, AD07011,  AD07012, AD07789, AD07790, AD07791, AD08108,  AD08109, AD08245, AD08383, AD08385, AD08559,  AD08625, AD08626, AD08743, AD10276, AD10277, AD10278, AD10279, AD10280, AD10281 |
|  |  |
