# Supplementary material for: The Genome of Anopheles darlingi, the main neotropical malaria vector
Source: Nucleic Acids Res. 2013 Jun 12;41(15):7387–400. doi: 10.1093/nar/gkt484 (PMC3753621; doi:10.1093/nar/gkt484)
Supplement: Supplementary Data [file supp_gkt484_nar-00257-h-2013-File006_updated.zip › S-A.docx]

**S-A Methods**

**Genomic DNA Extraction**

Four hundred *An. darlingi* mosquitoes were captured in Coari city (4°04'56''S - 63°08'34''W, Amazonas, Brazil). Gravid females were transferred to the laboratory (INPA, Manaus) and were allowed to lay eggs in plastic cups that were filled with distilled water. Larvae were fed powdered Tetramin, and pupae were transferred to plastic cups that were filled with distilled water. Genomic DNA was extracted from 1,884 recently emerged adults, males and females, according to [96]. The DNA integrity was analyzed by agarose gel electrophoresis, and concentrations were determined by absorption at 260-nm with an ND-1000 spectrometer (Nanodrop Technologies, Wilmington, DE).

**Genomic DNA sequencing and genome assembly**

Libraries constructions and sequencing on a Roche/454 GS FLX were performed at 454 Life Sciences/Roche Branford, CT, USA, following standard protocols. The resulting data set consisted of 14,883,218 whole genome shotgun Roche/454 reads (FLX Standard and FLX Titanium) and 4,905,784 Roche/454 paired-end reads that were derived from FLX Standard 3Kb libraries (Table S1). WGS-assembler software (Celera Assembler v. 6.1) was used to assemble the shotgun and paired-end Roche/454 reads into 13,857 contigs (N50 = 37.754) and 8,233 scaffolds (N50 = 81.222), spanning 177.61 Mpb as determined (Tables 1 and S2). Approximately 4.7 Mbp (2.6%) with high similarity to bacterial genomes were identified and removed prior and also post assembly. The assembly was further evaluated using a fosmids library containing 35kb long inserts. Two hundred paired-sequences were mapped to the genome of which 111 had both ends within the same scaffolds, with the average size of 34,418 ± 3,958 bp. The remaining clones mapped to scaffolds ends.

**RNA-Seq and transcriptome assembly**

The *An. darlingi* transcriptome was derived from two RNA samples:

**Sample 1 -** *Anopheles darlingi* mosquitoes were captured in Coari city (4°04'56''S - 63°08'34''W, Amazonas, Brazil), the same locality from where mosquito genomic DNA was extracted. Larvae were fed powdered Tetramin, and adult females were fed with blood on *Gallus gallus domesticus*. Whole mosquitoes were frozen at 4, 8, 14, 24 and 36 hours after blood feeding and stored at -80ºC for subsequent analysis. Total RNA extraction was performed with 100 mg of mosquitoes from each point in the time-course using the QIAGEN RNeasy kit following the manufacturer’s instructions. Total RNA extraction was quantified using a spectrophometric method, and RNA integrity was evaluated in denaturing agarose gel. All of the RNA samples were sent to Ambry Genetics Genomic Services (http://www.ambry.com) for library construction and subsequent 100bp paired-end sequencing in an Illumina HiSeq2000.

**Sample 2** - *Anopheles darlingi* mosquitoes were captured in Porto Velho city (8°45'36''S - 63°54'36''W, Rondonia, Brazil); Total RNA was extracted by Trizol from 195 head-less adult mosquitoes and was further isolated using the PolyATract - mRNA Isolation System III from Promega, according to the manufacturer’s directions. A strand-specific library was generated using the Directional mRNA-seq library kit (Illumina). PolyA+ RNA was fragmented and ligated to RNA adapters. Reverse transcription was performed using a primer that was complementary to the 3’ adapter sequence. The libraries were PCR amplified and sequenced on an Illumina HiSeq2000, yielding 79,762,682 paired-end reads of 76 nt length. A second library was sequenced by Roche/454 GS FLX technology, yielding 1,318,371 paired-end reads of 245 median lengths.

Illumina reads derived from samples 1 and 2 were processed separately. The seven last bases of the reads derived from sample 1 were trimmed because they had a low median quality value. Illumina reads were aligned to the *An. darlingi* genome using GSNAP with the following parameters values: 3 mismatches were allowed, read trimming was turned off and novel splice junctions were defined in case two segments from the same read of at least 14 nt length aligned apart from each other on the reference genome. Mate pair alignments were separated according to strand and were submitted to two independent runs of Scripture, an application that reconstructs gene models from RNA-Seq short read alignments.

Reads generated by 454 sequencing of sample 2 were assembled using Velvet/Oases, using the following parameter values: data set segmented in kmers of 21 nt of length (velveth parameter: “-k 21”), long reads input enabled (velveth parameter: “-long”), read tracking enabled (velvetg: “-read_trkg yes”) and scaffolding turned off (oases: “-scafolding no”).

**Gene prediction**

Gene prediction was performed using Glimmer, trained with the *An. gambiae* gene set, and Augustus, trained with *An. darlinigi* transcripts aligned against the reference genome. The transcript evidence used by Augustus consisted of 2,576 expressed-sequence tags (EST) downloaded from NCBI-Genbank on September 2010 and transcript assemblies derived from RNA-Seq.

The gene prediction was further improved by transcript evidence incorporation using PASA: complete and partial transcripts were aligned to their respective genomic loci and assembled based on sequence similarity; predicted gene models were then modified according to transcript evidence.

PASA transcript incorporation was performed in two rounds: initial gene prediction models were improved by strand-specific transcripts, the resulting gene set was then further improved by a second round of PASA using strandless transcripts. The strand-specific data set was composed of gene models that were defined by Scripture according to RNA-Seq data from sample 2. The strandless data set comprised assemblies of 454 reads (sample 2), Scripture gene sequences derived from sample 1 and the EST data set mentioned above.

A second round of gene prediction was performed by Glimmer and Augustus. Both applications were trained with transcript assemblies generated by PASA.

Gene models based on similarity to dipterans orthologs, an alternative set of *An. darlingi* gene models, were defined by Exonerate based on exon-aware alignment *of An. gambiae, Cu. quinquefasciatus, Ae. aegypti* and *D. melanogaster* protein sequences against the *An. darlingi* genome. A minimum threshold of 60% of the highest score possible for each alignment was used. The complete set of conceptually translated genes of An. gambiae*, Cu. quinquefasciatus, Ae. aegypti* and *D. melanogaster* genes were downloaded from Ensembl FTP site release 8 (ftp://ftp.ensemblgenomes.org), VectorBase version 1.3 (ftp://ftp.vectorbase.org), VectorBase version 1.3 or FlyBase version 5.25 (ftp://ftp.flybase.net).

Combining *An. darlingi* alternative gene sets – All alternative gene sets were combined into a single gene set using Evidence Modeller. PASA-generated models had the highest weight at defining the correct structure of the gene, followed by Exonerate-based models according to the following species order: An. gambiae*, Ae. aegypti, Cu. quinquefasciatus* and *D. melanogaster.* Augustus gene predictions had the second to the lowest weight, followed by Glimmer predictions. The final gene set was compared with those of other mosquito species (Tables SA-3 and 4).

The completeness of the genome sequence and the quality of the structural annotation was appraised by CEGMA [34]. This application identifies 248 conserved eukaryotic genes. Next, it produces a high quality de novo structural annotation for those genes based on HMM profiles derived from orthologs. Finally, CEGMA reports how many of those genes have integral loci, meaning that they represent more than 70% of the length of their respective HMM profile, and how many are partially represented, below 70% coverage. The integral gene loci found in the *An. darlingi* genome were then used to evaluate the quality of the structural annotation. The coding sequences of those genes, according to CEGMA, were aligned by BLASTP to the coding sequences of the same genes based on our annotation pipeline. The number of identical amino acids between genes predicted by the concurrent methods was computed and reported.

The integral locus sequences of 235 highly conserved eukaryotic genes were identified in the *An. darlingi* genome assembly. Other 8 highly conserved genes were found as partial loci. Approximately 62% of those genes are identical between methods and 91% have more than 80% sequence identity.

**Evaluation of synteny**

Pairwise syntenic blocks between the genomes of *An. darlingi* and *An. gambiae, Cu. quinquefasciatus, Ae. aegypti, and D. melanogaster* were defined by DAGchainer using default values on all of the parameters except for “minimum number of colinear genes”, which was set to 3 (-A 3). The *An. darlingi* genomic scaffolds having the longest alignment against chromosome 2R of *An. gambiae* were selected from whole genome alignments performed by progressiveMauve, with default parameters. The extent of the alignment between a pair of sequences was defined as the length spanning all of their respective collinear blocks.

**Single nucleotide variants (SNVs)**

Samtools package version 0.1.18 was used to call the SNVs using a BAM sorted and converted file through the algorithm *mpileup*. This last algorithm generates a BCF binary file containing the nucleotidic variation for all reads when compared to the reference genome. *Mpileup* considers, as the default parameter, an SNV to be called in any variant observed in at least 3 different reads. A VCF (*Variant Call Format*) was extracted from the BCF file with a maximum coverage number of 100 reads per SNV. Finally, the *snpEFF* software (available at http://snpeff.sourceforge.net) generated a report that described all SNVs that were found in VCF format. Thus, PERL scripts were developed to compare the coordinates of genomic SNVs to positions mapping predicted genes in the GFF genome file. SNVs were considered to be valid only when at least 3 reads were found to support the base call, and the minimum quality of the base was 40. More than 10 million SNVs were reported in the *An. darlingi* genome, 84% of them identified using the genomic reads and the remaining revealed by RNAseq data (Table S-A5).

**Table S-A1. *Anopheles darlingi* whole genome data set.**

| **# Roche/454 run** | **# Shotgun reads** | **Paired-end reads^1,2^** | **Base Pairs** | **Genome coverage (X)** |
| --- | --- | --- | --- | --- |
| 44 FLX Standard | 9,061,157 | 4,905,784 | 3,301,417,472 | 18^3^ |
| 6 FLX Titanium | 5,822,561 | - | 2,252,201,731 | 12 |
| Total | 14,883,718 | 4,905,784 | 5,553,619,203 | 30 |

^1^ - 1,035,196 fragments with paired ends

^2^ - Average insert size: 2550 bp

^3^ - Coverage by fragments from paired-end library: 15 x

**Table S-A2. Assembly statistics of *Anopheles darlingi* reference genome**

| **Feature** | **Statistics** |
| --- | --- |
| Total number of good sequence reads | 16,777,488 |
| Sequence reads in assembly | 14,139,351 |
| Total number of scaffolds | 8,233 |
| Total bases in scaffolds | 173,918,288 (including the estimated size of the gaps) |
| Total number of contigs | 13,857 |
| Combined length of contigs | 172,639,290 |
| Length of sequence gaps in scaffolds | 1,278,998 |
| Sequencing coverage | 20 |
| N50 scaffold length | 81,222 |
| N50 contig length | 37,754 |
| Longest scaffold (number of contigs) | 1,087,588 (10) |
| Shortest scaffold (number of contigs) | 473 (1) |

**Table S-A3. *Anopheles darlingi* protein coding genes best matches in KEGG database, by organism.**

| **Organism** | **Number of protein-coding genes** |
| --- | --- |
| *An. gambiae* | 7,576 (72.28%)* |
| *Ae. aegypti* | 1,206 (11.50%) |
| *Cu. quinquefasciatus* | 1,028 (9.80%) |

*Percentages calculated from the total (10,481) predicted protein-coding genes, including 13 mitochondrial genes.

**Table S-A4. Coverage of best matches of the *Anopheles darling* genes in KEGG database, by organisms.**

| **Query Coverage** | **Subject Coverage** | **Percentage of annotated genes with KEGG hits** | **Best match with *An. gambiae* (%)** | **Best match with *Ae. aegypti* (%)** | **Best match with *Cu. quinquefasciatus* (%)** | **Best match with other organisms (%)** |
| --- | --- | --- | --- | --- | --- | --- |
| ≥ 90 % | ≥ 90 % | 54.65 | 43.56 | 5.83 | 4.53 | 0.68 |
| ≥ 80 % | ≥ 80 % | 65.41 | 51.35 | 7.30 | 5.68 | 1.06 |
| ≥ 70 % | ≥ 70 % | 71.91 | 55.58 | 8.27 | 6.54 | 1.50 |
| ≥ 60 % | ≥ 60 % | 76.70 | 58.85 | 8.85 | 7.20 | 1.78 |
| ≥ 50 % | ≥ 50 % | 80.28 | 61.07 | 9.42 | 7.60 | 2.18 |

*Percentages calculated from the total (10,481) predicted protein-coding genes, including 13 mitochondrial genes.

**TableS-A5. Number of SNVs per genomic feature.**

| **Genomic Feature** | **Genomic (454)*** | **TOTAL transcriptome** | **PV454**** | **PVI**** | **COI**** |
| --- | --- | --- | --- | --- | --- |
| **Gene** | 1,643,685  19,44%  39.7 per Kb | 819,427  49,52%  19.8 per Kb | 120,323  57,91%  2.91 per Kb | 328,325  50,16%  7.93 per Kb | 370,779  46,79%  8.95 per Kb |
| **Exon** | 488,652  5,78%  26.2 per Kb | 494,539  29,88%  26,6 per Kb | 110,879  53,36%  5.96 per Kb | 211,742  32.35%  11.37 per Kb | 171,918  21,69%  9.23 per Kb |
| **Intron** | 1,155,083  13.66%  50.7 per Kb | 324,926  19,63%  14.2 per Kb | 9,446  4.55%  0.42 per Kb | 116,602  17.81%  5.12 per Kb | 198,878  25,10%  8.73 per Kb |
| **CDS** | 475,903  5.63%  26.1 per Kb | 481,588  29.10%  26.37 per Kb | 108,746  52.33%  5.96 per Kb | 206,447  31.54%  11.31 per Kb | 166,395  21,00%  9.11 per Kb |
| **Intergenic** | 6,811,677  80.56%  50.0 per Kb | 835,447  50.48%  6.1 per Kb | 87,460  42.09%  0.64 per Kb | 326,294  49,85%  2.40 per Kb | 421,693  53,21%  3.10 per Kb |
| **Promoter** (2Kb upstream from transcript 5’-end) | 360,607  4.27%  41.8 per Kb | 153,431  9.27%  17.8 per Kb | 17,563  8.45%  2.04 per Kb | 60,120  9.18%  6.97 per Kb | 75,748  9.56%  8.79 per Kb |
| **TOTAL (genic + intergenic)** | **8,455,362** | **1,654,874** | **207,783** | **654,619** | **792,472** |

*Genome data from mosquitoes collected in Coari. **Transcriptome data from Porto Velho (PV) or Coari (CO) and samples were sequenced by either 454 Life Science (454) or Illumina (I) technologies.
