# Supplementary material for: The Genome of Anopheles darlingi, the main neotropical malaria vector
Source: Nucleic Acids Res. 2013 Jun 12;41(15):7387–400. doi: 10.1093/nar/gkt484 (PMC3753621; doi:10.1093/nar/gkt484)
Supplement: Supplementary Data [file supp_gkt484_nar-00257-h-2013-File006_updated.zip › S-B.docx]

**S-B – *Anopheles darlingi* genome size determination by flow cytometry**

The nuclear DNA content of diploid cells of *An. darlingi* from Coari, AM, was measured using standard samples of *D. melanogaster* female strain Iso-1 (2C = 0.36 pg, [19] obtained from the University of California at Berkeley (CA, USA). FCM nuclei suspensions were prepared according to [97]. Basically, drosophila and anopheles brain ganglia were excised in physiologic solution (0.155 mM NaCl). The materials were simultaneously crushed 10 times with a pestle in a tissue grinder (Kontes Glass Company) with 100 μL OTTO-I lysis buffer [98] containing 0.1 M citric acid, 0.5% Tween 20 and 50 μg/ml RNAse-A, pH = 2.3. The suspension was filtered through 30 μm nylon mesh (Partec) and centrifuged at 100x*g* for 5 min. The pellet was incubated for 10 min in 100 μl OTTO-I lysis buffer and was stained for 30 min with 1.5 ml OTTO-I:OTTO-II (1:2) solution [99] supplemented with 75 μM propidium iodide (PI) and 50 μg/ ml RNAse-A, pH = 7.8. The nuclear suspension was then analyzed with a PAS flow cytometer (Partec) equipped with a Laser source (488 nm). PI fluorescence emitted from the nuclei was collected through an RG 610 nm band-pass filter and was converted to 1024 channels.

The equipment was calibrated for linearity and was aligned with microbeads and standard solutions according to the manufacturer’s recommendations. FlowMax software (Partec) was used for the data analyses. The standard nuclei peak was set to channel 200, and more than 10000 nuclei were analyzed. Three independent repetitions were conducted, and the mean genome size (pg) of the samples and standards were measured according to the formula adapted from [100] and were subsequently converted to megabases pairs (1 pg = 978 Mbp) [101]. Analyses were then performed on the nuclei suspensions stained with PI, generating histograms with peaks that correspond to the average relative DNA content of the G_0_/G_1_ nuclei of the *An. darlingi* female and the comparative internal standard (*D. melanogaster* female) (Fig. 1). The histograms showed good resolution levels (CVs ranging from 2.32 to 3.17%). The mean nuclear genome size value of *An. darlingi* (female) was estimated as 2C = 0.41 pg (or 1C= 200.49 Mbp).


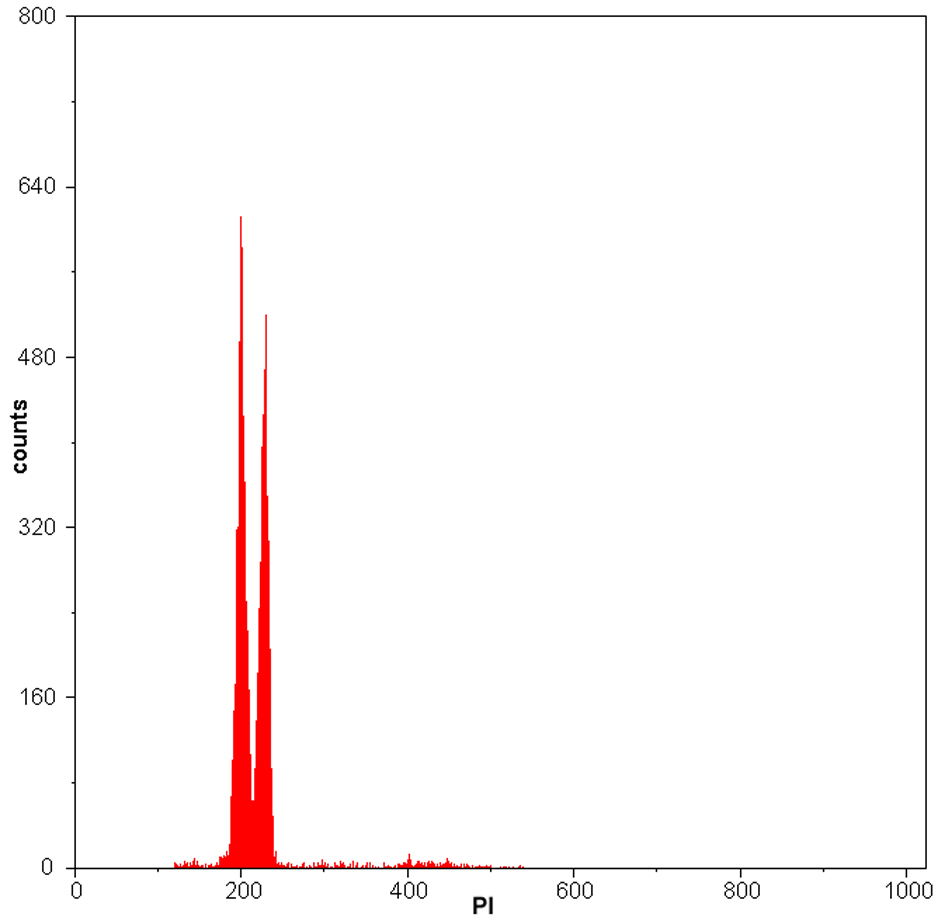


**Figure S-B1.** **Genome size determination through analysis of nuclear suspensions of cerebral ganglion tissue stained with PI.** DNA-histogram of *D. melanogaster* (channel 200), used as internal standard and *An. darlingi* (channel 228).
