# Supplementary material for: The Genome of Anopheles darlingi, the main neotropical malaria vector
Source: Nucleic Acids Res. 2013 Jun 12;41(15):7387–400. doi: 10.1093/nar/gkt484 (PMC3753621; doi:10.1093/nar/gkt484)
Supplement: Supplementary Data [file supp_gkt484_nar-00257-h-2013-File006_updated.zip › S-C.docx]

**S-C Mitochondrial genome alignment**

**GQ918273.1 AATGAATTGCCTGATGAAAAGGATTACCTTGATAGGGTAAATCATGAAATTTATAATTTC 60**

**mtAD AATGAATTGCCTGATGAAAAGGATTACCTTGATAGGGTAAATCATGAAATTTATAATTTC 60**

**GQ918272.1 AATGAATTGCCTGATGAAAAGGATTACCTTGATAGGGTAAATCATGAAATTTATAATTTC 60**

****************************************************************

**GQ918273.1 ATTCATTATATTTAACAGAATTAAACTGTTTCCAAAAGAATCAAAATCTATTGTGCTTCG 120**

**mtAD ATTCATTATATTTAACAGAATTAAACTGTTTCCAAAAGAATCAAAATCTATTGTGCTTCG 120**

**GQ918272.1 ATTCATTATATTTAACAGAATTAAACTGTTTCCAAAAGAATCAAAATCTATTGTGCTTCG 120**

****************************************************************

**GQ918273.1 TACACTAAAATATAAAAAGATAAGCTAAATAAGCTATTGGGTTCATACCCCACTTATAAA 180**

**mtAD TACACTAAAATATAAAAAGATAAGCTAAATAAGCTATTGGGTTCATACCCCACTTATAAA 180**

**GQ918272.1 TACACTAAAATATAAAAAGATAAGCTAAATAAGCTATTGGGTTCATACCCCACTTATAAA 180**

****************************************************************

**GQ918273.1 GGTTATAATCCTTTTCTTTTTAATTAAAAAAATTTCAAACAATATTTTTATTATTACTTT 240**

**mtAD GGTTATAATCCTTTTCTTTTTAATTAAAAAAATTTCAAACAATATTTTTATTATTACTTT 240**

**GQ918272.1 GGTTATAATCCTTTTCTTTTTAATTAAAAAAATTTCAAACAATATTTTTATTATTACTTT 240**

****************************************************************

**GQ918273.1 AATATTTGGAACATTAATTACTATTTCTTCAAATTCTTGATTAGGAGCTTGAATAGGGTT 300**

**mtAD AATATTTGGAACATTAATTACTATTTCTTCAAATTCTTGATTAGGAGCTTGAATAGGGTT 300**

**GQ918272.1 AATATTTGGAACATTAATTACTATTTCTTCAAATTCTTGATTAGGAGCTTGAATAGGGTT 300**

****************************************************************

**GQ918273.1 AGAAATTAATTTATTATCATTTATCCCCCTAATAAATGATAATAAAAAAAATTTACTAAC 360**

**mtAD AGAAATTAATTTATTATCATTTATCCCCCTAATAAATGATAATAAAAAAAATTTACTAAC 360**

**GQ918272.1 AGAAATTAATTTATTATCATTTATCCCCCTAATAAATGATAATAAAAAAAATTTACTAAC 360**

****************************************************************

**GQ918273.1 TTCAGAAAGTTCATTAAAATATTTTTTAACTCAAGCTTTTGCTTCATCAATTCTATTGTT 420**

**mtAD TTCAGAAAGTTCATTAAAATATTTTCTAACTCAAGCTTTTGCTTCATCAATTCTATTGTT 420**

**GQ918272.1 TTCAGAAAGTTCATTAAAATATTTTCTAACTCAAGCTTTTGCTTCATCAATTCTATTGTT 420**

*************************** ************************************

**GQ918273.1 TGCAATTATTATTTTAATATTTTTTTATAATAATAATTTTTCACAATTTTATAGACTTAA 480**

**mtAD TGCAATTATTATTTTAATATTTTTTTATAATAATAATTTTTCACAATTTTATAGACTTAA 480**

**GQ918272.1 TGCAATTATTATTTTAATATTTTTTTATAATAATAATTTTTCACAATTTTATAGACTTAA 480**

****************************************************************

**GQ918273.1 TGAAATTTTAATTTTATCAACTTTAATATTAAAGAGAGGAGCAGCTCCTTTTCATTTTTG 540**

**mtAD TGAAATTTTAATTTTATCAACTTTAATATTAAAGAGAGGAGCAGCTCCTTTTCATTTTTG 540**

**GQ918272.1 TGAAATTTTAATTTTATCAACTTTAATATTAAAGAGAGGAGCAGCTCCTTTTCATTTTTG 540**

****************************************************************

**GQ918273.1 GTTCCCTGAAGTAATGGAAGGTCTATCTTGAATTAATGGACTAATTTTAATAACTTGACA 600**

**mtAD GTTCCCTGAAGTAATAGAAGGTCTATCTTGAATTAATGGACTAATTTTAATAACTTGACA 600**

**GQ918272.1 GTTCCCTGAAGTAATGGAAGGTTTATCTTGAATTAATGGACTAATTTTAATAACTTGACA 600**

***************** ****** ***************************************

**GQ918273.1 AAAAATTGCTCCTTTAATATTAATTTCATATAATTTTTGTTATAATTTTTTTATAATATC 660**

**mtAD AAAAATTGCTCCTTTAATATTAATTTCATATAATTTTTGTTATAATTTTTTTATAATATC 660**

**GQ918272.1 AAAAATTGCTCCTTTAATATTAATTTCATATAATTTTTGTTATAATTTTTTTATAATATC 660**

****************************************************************

**GQ918273.1 AATTATTTTATCAATACTAATTGGATCATTAGGAGGATTAAATCAAACTTCAATTCGTAA 720**

**mtAD AATTATTTTATCAATACTAATTGGATCATTAGGAGGATTAAATCAAACTTCAATTCGTAA 720**

**GQ918272.1 AATTATTTTATCAATACTAATTGGATCATTAGGAGGATTAAATCAAACTTCAATTCGTAA 720**

****************************************************************

**GQ918273.1 ATTAATAGCTTTTTCATCAATTAATCATTTAGGATGAATATTATTAGCAATAATAAATAA 780**

**mtAD ATTAATAGCTTTTTCATCAATTAATCATTTAGGATGAATATTATTAGCAATAATAAATAA 780**

**GQ918272.1 ATTAATAGCTTTTTCGTCAATTAATCATTTAGGATGAATATTATTAGCAATAATAAATAA 780**

***************** **********************************************

**GQ918273.1 TGAGTTATTATGAATAACATATTTTTTATTATATTCATTATTATCAATTTCAATTATTAT 840**

**mtAD TGAGTTATTATGAATAACATATTTTTTATTATATTCATTATTATCAATTTCAATTATTAT 840**

**GQ918272.1 TGAGTTATTATGAATAACATATTTTTTATTATACTCATTATTATCAATTTCAATTATTAT 840**

*********************************** ****************************

**GQ918273.1 AATATTTAATAATTTTAAATTATTTTATTTTAATCAAATTTTTAATATTTCAATAATAAA 900**

**mtAD AATATTTAATAATTTTAAATTATTTTATTTTAATCAAATTTTTAATATTTCAATAATAAA 900**

**GQ918272.1 AATATTTAATAATTTTAAATTATTTTATTTTAATCAAATTTTTAATATTTCAATAATAAA 900**

****************************************************************

**GQ918273.1 TCCAATTATTAAATTTTTAATTTTTTTAAATTTATTATCATTAGGAGGATTGCCTCCATT 960**

**mtAD TCCAATTATTAAATTTTTAATTTTTTTAAATTTATTATCATTAGGAGGATTGCCTCCATT 960**

**GQ918272.1 TCCAATTATTAAATTTTTAATTTTTTTAAATTTATTATCATTAGGAGGATTGCCTCCATT 960**

****************************************************************

**GQ918273.1 TTTAGGATTTTTACCAAAATGATTAGTAATTCAAAACTTAACTAGTATAAATCAATTATT 1020**

**mtAD TTTAGGATTTTTACCAAAATGATTAGTAATTCAAAACTTAACTAGTATAAATCAATTATT 1020**

**GQ918272.1 TTTAGGATTTTTACCAAAATGATTAGTAATTCAAAACTTAACTAGTATAAATCAATTATT 1020**

****************************************************************

**GQ918273.1 TATTTTAACTATTTCAGTTTGTTTAACATTAATTACTTTATATTTTTATTTACGATTATC 1080**

**mtAD TATTTTAACTATTTCAGTTTGTTTAACATTAATTACTTTATATTTTTATTTACGATTATC 1080**

**GQ918272.1 TATTTTAACTATTTCAGTTTGTTTAACATTAATTACTTTATATTTTTATTTACGATTATC 1080**

****************************************************************

**GQ918273.1 TTACAGTATTTTTATGCTAAATTATCAAAAAAATACATGAATATTAAAAAATAATTACAA 1140**

**mtAD TTACAGTATTTTTATGTTAAATTATCAAAAAAATACATGAATATTAAAAAATACTTACAA 1140**

**GQ918272.1 TTACAGTATTTTTATGTTAAATTATCAAAAAAATACATGAATATTAAAAAATACTTACAA 1140**

****************** ************************************ ********

**GQ918273.1 TATAAAAATATCATCAATAAGCTTAATTTTAAATTTTATTTCAATTGGGGGATTATTAAT 1200**

**mtAD CATAAAAATATCATCAATAAGCTTAATTTTAAATTTTATTTCAATTGGGGGATTATTAAT 1200**

**GQ918272.1 TATAAAAATATCATCAATAAGTTTAATTTTAAATTTTATTTCAATTGGGGGATTACTAAT 1200**

********************** ********************************* ******

**GQ918273.1 AATTTTAATATTTTATATAATTTTATAAGAATTTAAGTTAAATAAACTAATAGCCTTCAA 1260**

**mtAD AATTTTAATATTTTATATAATTTTATAAGAATTTAAGTTAAATAAACTAATAGCCTTCAA 1260**

**GQ918272.1 AATTTTAATATTTTATATAATTTTATAAGAATTTAAGTTAAATAAACTAATAGCCTTCAA 1260**

****************************************************************

**GQ918273.1 AGCTGAAAATATTTGTATTAATCTTTTAATTCTTAAGCTTTAATAAATTATTTATTCCTT 1320**

**mtAD AGCTGAAAATATTTGTATTAATCTTTTAATTCTTAAGCTTTAATAAATTATTTATTCCTT 1320**

**GQ918272.1 AGCTGAAAATATTTGTATTAATCTTTTAATTCTTAAGCTTTAATAAATTATTTATTCCTT 1320**

****************************************************************

**GQ918273.1 TAGAATTGCAGTCTAATATCATTATTGACTATAAAGCCTGATTAAAGAGATAAATCCCAT 1380**

**mtAD TAGAATTGCAGTCTAATATCATTATTGACTATAAAGCCTGATTAAAGAGATAATTCCCAT 1380**

**GQ918272.1 TAGAATTGCAGTCTAATATCATTATTGACTATAAAGCCTGATTAAAGAGATAAATCCCAT 1380**

******************************************************* ********

**GQ918273.1 AAATAAATTTACAATTTATTGCCTAAACTTCAGCCATTTAATCGCGACAATGATTATTTT 1440**

**mtAD AAATAAATTTACAATTTATTGCCTAAACTTCAGCCATTTAATCGCGACAATGATTATTTT 1440**

**GQ918272.1 AAATAAATTTACAATTTATTGCCTAAACTTCAGCCATTTAATCGCGACAATGATTATTTT 1440**

****************************************************************

**GQ918273.1 CAACAAATCATAAGGATATTGGAACATTATATTTTATTTTTGGAGCTTGAGCTGGAATAG 1500**

**mtAD CAACAAATCATAAGGATATTGGAACATTATATTTTATTTTTGGAGCTTGAGCTGGAATAG 1500**

**GQ918272.1 CAACAAATCATAAGGATATTGGAACATTATATTTTATTTTTGGAGCTTGAGCTGGAATAG 1500**

****************************************************************

**GQ918273.1 TTGGAACTTCATTAAGAATTTTAATTCGTGCTGAATTAGGTCATCCTGGAGCATTTATTG 1560**

**mtAD TTGGAACTTCATTAAGAATTTTAATTCGTGCTGAATTAGGTCATCCTGGAGCATTTATTG 1560**

**GQ918272.1 TTGGAACTTCATTAAGAATTTTAATTCGGGCTGAATTAGGTCATCCTGGAGCATTTATTG 1560**

****************************** *********************************

**GQ918273.1 GTGATGATCAAATTTATAATGTAATTGTTACAGCACATGCATTTATTATAATTTTTTTTA 1620**

**mtAD GTGATGATCAAATTTATAATGTAATTGTTACAGCACATGCATTTATTATAATTTTTTTTA 1620**

**GQ918272.1 GTGATGATCAAATTTATAATGTAATTGTTACAGCACATGCATTTATTATAATTTTTTTTA 1620**

****************************************************************

**GQ918273.1 TAGTAATACCAATTATAATTGGAGGATTTGGTAATTGATTAGTGCCTTTAATATTAGGAG 1680**

**mtAD TAGTAATACCAATTATAATTGGAGGATTTGGTAATTGATTAGTGCCTTTAATATTAGGAG 1680**

**GQ918272.1 TAGTAATACCAATTATAATTGGAGGATTTGGTAATTGATTAGTGCCTTTAATATTAGGAG 1680**

****************************************************************

**GQ918273.1 CTCCTGATATGGCTTTCCCACGAATAAATAATATAAGTTTTTGAATACTTCCTCCTTCTT 1740**

**mtAD CTCCTGATATGGCTTTCCCACGAATAAATAATATAAGTTTTTGAATACTTCCTCCTTCTT 1740**

**GQ918272.1 CTCCTGATATGGCTTTCCCACGAATAAACAATATAAGTTTTTGAATACTTCCCCCTTCTT 1740**

****************************** *********************** *********

**GQ918273.1 TAACTTTATTAATTTCTAGAAGTATAGTAGAAAATGGAGCTGGAACAGGATGAACTGTTT 1800**

**mtAD TAACTTTATTAATTTCTAGAAGTATAGTAGAAAATGGAGCTGGAACAGGATGAACTGTTT 1800**

**GQ918272.1 TAACTTTATTAATTTCTAGAAGTATAGTAGAAAATGGAGCTGGAACAGGATGAACTGTTT 1800**

****************************************************************

**GQ918273.1 ATCCTCCTCTATCATCAGGAATTGCTCATGCTGGAGCTTCTGTTGATTTAGCTATTTTTT 1860**

**mtAD ATCCTCCTCTATCATCAGGAATTGCTCATGCTGGAGCTTCTGTTGATTTAGCTATTTTTT 1860**

**GQ918272.1 ATCCTCCTCTATCATCAGGAATTGCTCATGCTGGAGCTTCTGTTGATTTAGCTATTTTTT 1860**

****************************************************************

**GQ918273.1 CTCTTCATTTAGCAGGAATTTCATCTATTTTAGGAGCAGTAAATTTTATTACTACAGTAA 1920**

**mtAD CTCTTCATTTAGCAGGAATTTCATCTATTTTAGGAGCAGTAAATTTTATTACTACAGTAA 1920**

**GQ918272.1 CTCTTCATTTAGCAGGAATTTCATCTATTTTAGGAGCAGTAAATTTTATTACTACAGTAA 1920**

****************************************************************

**GQ918273.1 TTAATATACGATCTCCAGGAATTACTTTAGATCGAATACCATTGTTTGTATGATCAGTAG 1980**

**mtAD TTAATATACGATCTCCAGGAATTACTTTAGATCGAATACCATTGTTTGTATGATCAGTAG 1980**

**GQ918272.1 TTAATATACGATCTCCAGGAATTACTTTAGATCGAATACCATTATTTGTATGATCAGTAG 1980**

********************************************* ******************

**GQ918273.1 TAATTACTGCTATTTTATTATTATTATCTTTACCTGTATTAGCTGGAGCTATTACTATAT 2040**

**mtAD TAATTACTGCTATTTTATTATTATTATCTTTACCTGTATTAGCTGGAGCTATTACTATAT 2040**

**GQ918272.1 TAATTACTGCTATTTTATTATTATTATCTTTACCTGTATTAGCTGGAGCTATTACTATGT 2040**

************************************************************ ***

**GQ918273.1 TATTAACAGATCGAAATTTAAATACTTCTTTCTTTGATCCAGCTGGAGGAGGAGACCCTA 2100**

**mtAD TATTAACAGATCGAAATTTAAATACTTCTTTCTTTGATCCAGCTGGAGGAGGAGACCCTA 2100**

**GQ918272.1 TATTAACAGACCGAAATTTAAATACTTCTTTCTTTGATCCAGCCGGAGGAGGAGATCCTA 2100**

************ ******************************** *********** ******

**GQ918273.1 TTCTATACCAACATTTATTTTGATTTTTTGGACATCCTGAAGTTTACATTTTAATTTTAC 2160**

**mtAD TTCTATACCAACATTTATTTTGATTTTTTGGACATCCTGAAGTTTACATTTTAATTTTAC 2160**

**GQ918272.1 TTCTATATCAACATTTATTTTGATTTTTTGGACATCCTGAAGTTTACATTTTAATTTTAC 2160**

********* ******************************************************

**GQ918273.1 CAGGATTTGGAATGATTTCACATATTATTACTCAAGAAAGAGGAAAAAAGGAAACTTTTG 2220**

**mtAD CAGGATTTGGAATGATTTCACATATTATTACTCAAGAAAGAGGAAAAAAGGAAACTTTTG 2220**

**GQ918272.1 CAGGATTTGGAATAATTTCACATATTATTACTCAAGAAAGAGGAAAAAAGGAAACTTTTG 2220**

*************** ************************************************

**GQ918273.1 GAAATTTAGGAATAATTTATGCAATATTAGCCATTGGATTATTAGGATTTATTGTATGAG 2280**

**mtAD GAAATTTAGGAATAATTTATGCAATATTAGCCATTGGATTATTAGGATTTATTGTATGAG 2280**

**GQ918272.1 GAAATTTAGGAATAATTTATGCAATATTAGCCATTGGATTATTAGGATTTATTGTATGAG 2280**

****************************************************************

**GQ918273.1 CTCATCATATATTTACTGTTGGTATAGATGTAGATACACGAGCTTATTTTACTTCTGCTA 2340**

**mtAD CTCATCATATATTTACTGTTGGTATAGATGTAGATACACGAGCTTATTTTACTTCTGCTA 2340**

**GQ918272.1 CTCATCATATATTTACTGTTGGTATAGATGTAGATACACGAGCTTATTTTACTTCTGCTA 2340**

****************************************************************

**GQ918273.1 CTATAATTATTGCTGTGCCTACAGGAATTAAAATTTTTAGTTGATTAGCTACTCTTCATG 2400**

**mtAD CTATAATTATTGCTGTGCCTACAGGAATTAAAATTTTTAGTTGATTAGCTACTCTTCATG 2400**

**GQ918272.1 CTATAATTATTGCCGTACCTACAGGAATTAAAATTTTTAGTTGATTAGCTACTCTTCATG 2400**

*************** ** *********************************************

**GQ918273.1 GTACTCAATTAACATATAGCCCAGCTATACTATGAGCCTTTGGATTTGTCTTTTTATTTA 2460**

**mtAD GTACTCAATTAACATATAGCCCAGCTATACTATGAGCCTTTGGATTTGTCTTTTTATTTA 2460**

**GQ918272.1 GTACTCAATTAACATACAGCCCAGCTATATTATGAGCTTTTGGATTTGTCTTTTTATTTA 2460**

****************** ************ ******* ************************

**GQ918273.1 CTGTAGGAGGATTAACTGGAGTAGTATTAGCTAATTCTTCTATTGATATTGTATTACATG 2520**

**mtAD CTGTAGGAGGATTAACTGGAGTAGTATTAGCTAATTCTTCTATTGATATTGTATTACATG 2520**

**GQ918272.1 CTGTAGGGGGATTAACTGGGGTAGTATTAGCTAATTCTTCTATTGATATTGTATTACATG 2520**

********* *********** ******************************************

**GQ918273.1 ATACTTATTATGTAGTTGCTCATTTTCATTATGTATTATCAATAGGAGCTGTATTTGCAA 2580**

**mtAD ATACTTATTATGTAGTTGCTCATTTTCATTATGTATTATCAATAGGAGCTGTATTTGCAA 2580**

**GQ918272.1 ATACTTATTATGTAGTTGCTCATTTTCATTATGTATTATCAATAGGAGCTGTATTTGCAA 2580**

****************************************************************

**GQ918273.1 TTATAGCAGGATTTATTCATTGATACCCCTTATTAACAGGTTTAACTATAAATCCTACAT 2640**

**mtAD TTATAGCAGGATTTATTCATTGATACCCCTTATTAACAGGTTTAACTATAAATCCTACAT 2640**

**GQ918272.1 TTATAGCAGGATTTATTCATTGATACCCTCTATTAACAGGTTTAACTATAAATCCTACAT 2640**

****************************** ********************************

**GQ918273.1 GATTAAAAATTCAATTTTTTATAATATTTGTAGGAGTAAATTTAACATTTTTCCCTCAAC 2700**

**mtAD GATTAAAAATTCAATTTTTTATAATATTTGTAGGAGTAAATTTAACATTTTTCCCTCAAC 2700**

**GQ918272.1 GATTAAAAATTCAATTTTTTATAATATTTGTAGGAGTAAATTTAACATTTTTCCCTCAAC 2700**

****************************************************************

**GQ918273.1 ATTTCTTAGGATTAGCTGGTATACCTCGACGTTATTCAGATTTTCCAGACAGTTATTTAA 2760**

**mtAD ATTTCTTAGGATTAGCTGGTATACCTCGACGTTATTCAGATTTTCCAGACAGTTATTTAA 2760**

**GQ918272.1 ATTTCTTAGGATTAGCTGGTATACCTCGACGTTATTCAGATTTTCCAGATAGTTATTTAA 2760**

*************************************************** ************

**GQ918273.1 CTTGAAATATTGTTTCATCATTAGGTAGAACAATTTCATTATTTGCTATTTTATACTTTT 2820**

**mtAD CTTGAAATATTGTTTCATCATTAGGTAGAACAATTTCATTATTTGCTATTTTATACTTTT 2820**

**GQ918272.1 CTTGAAATATTGTTTCATCATTAGGTAGAACAATTTCATTATTTGCTATTTTATACTTTT 2820**

****************************************************************

**GQ918273.1 TATTTATTATTTGAGAAAGTATAATTACTCAACGAACTCCTGCTTTCCCAATACAATTAT 2880**

**mtAD TATTTATTATTTGAGAAAGTATAATTACTCAACGAACCCCTGCTTTCCCAATACAATTAT 2880**

**GQ918272.1 TATTTATTATTTGAGAAAGTATAATTACTCAACGAACCCCTGCTTTCCCAATACAATTAT 2880**

*************************************** ************************

**GQ918273.1 CTTCATCTATTGAATGATATCATACATTACCTCCTGCAGAACACACTTATGCAGAATTAC 2940**

**mtAD CTTCATCTATTGAATGATATCACACATTACCTCCTGCAGAACACACTTATGCAGAATTAC 2940**

**GQ918272.1 CTTCATCTATTGAATGATATCATACATTACCTCCTGCAGAACACACTTATGCAGAATTAC 2940**

************************ ***************************************

**GQ918273.1 CATTATTAACTAATAACTTCTAATATGGCAGATTAGTGCAATGAATTTAAGCTTCATATA 3000**

**mtAD CATTATTAACTAATAACTTCTAATATGGCAGATTAGTGCAATGAATTTAAGCTTCATATA 3000**

**GQ918272.1 CATTATTAACTAATAACTTCTAATATGGCAGATTAGTGCAATGAATTTAAGCTTCATATA 3000**

****************************************************************

**GQ918273.1 TAAAGATTTTATCTTTTGTTAGAATAATGGCAACATGAGCAAATTTAGGGTTACAAGATA 3060**

**mtAD TAAAGATTTTATCTTTTGTTAGAATAATGGCAACATGAGCAAATTTAGGGTTACAAGATA 3060**

**GQ918272.1 TAAAGATTTTATCTTTTGTTAGAATAATGGCAACATGAGCAAATTTAGGGTTACAAGATA 3060**

****************************************************************

**GQ918273.1 GTTCTTCTCCTTTAATAGAACAATTAAACTTTTTTCATGATCACACATTATTAATTTTAA 3120**

**mtAD GTTCTTCTCCTTTAATAGAACAATTAAACTTTTTTCATGATCACACATTATTAATTTTAA 3120**

**GQ918272.1 GTTCTTCTCCTTTAATAGAACAATTAAACTTTTTTCATGATCACACATTATTAATTTTAA 3120**

****************************************************************

**GQ918273.1 CTATAATTACAATTTTAGTTGGATATATTATAGGAATATTAATATTTAATAAATTTACTA 3180**

**mtAD CTATAATTACAATTTTAGTTGGATATATTATAGGAATATTAATATTTAATAAATTTACTA 3180**

**GQ918272.1 CTATAATTACAATTTTAGTTGGATATATTATAGGAATATTAATATTTAATAAATTTACTA 3180**

****************************************************************

**GQ918273.1 ATCGATATTTATTGCATGGACAAACTATTGAAATTATTTGAACAGTATTACCTGCAATTA 3240**

**mtAD ATCGATATTTATTGCATGGACAAACTATTGAAATTATTTGAACAGTATTACCTGCAATTA 3240**

**GQ918272.1 ATCGATATTTATTACATGGACAAACTATTGAAATTATTTGAACAGTATTACCTGCAATTA 3240**

*************** ************************************************

**GQ918273.1 TTTTAATATTTATTGCGTTCCCTTCATTACGATTATTATACCTAATAGATGAAATTAATA 3300**

**mtAD TTTTAATATTTATTGCGTTCCCTTCATTACGATTATTATACCTAATAGATGAAATTAATA 3300**

**GQ918272.1 TTTTAATATTTATTGCATTCCCTTCATTACGATTATTATACCTAATAGATGAAATTAATA 3300**

****************** *********************************************

**GQ918273.1 CTCCATCAATTACATTAAAGTCAGTAGGACATCAATGATATTGAAGTTATGAATATTCAG 3360**

**mtAD CTCCATCAATTACATTAAAGTCAGTAGGACATCAATGATATTGAAGTTATGAATATTCAG 3360**

**GQ918272.1 CTCCATCAATTACATTAAAGTCAGTAGGACATCAATGATATTGAAGTTATGAATATTCAG 3360**

****************************************************************

**GQ918273.1 ATTTTCTAAATTTAGAATTTGATTCATATATAATTCCAACAAATGAATTAGAATTAAGTG 3420**

**mtAD ATTTTCTAAATTTAGAATTTGATTCATATATAATTCCAACAAATGAATTAGAATTAAGTG 3420**

**GQ918272.1 ATTTTCTAAATTTAGAATTTGATTCCTATATAATTCCAACAAATGAATTAGAATTAAGTG 3420**

*************************** ************************************

**GQ918273.1 GATTTCGATTATTAGACGTTGATAATCGAGTAGTCTTACCAATGAATAATCAGATTCGAG 3480**

**mtAD GATTTCGATTATTAGACGTTGATAATCGAGTAGTCTTACCAATGAATAATCAAATTCGAG 3480**

**GQ918272.1 GATTTCGATTATTAGATGTTGATAATCGAGTAGTCTTACCAATAAATAATCAAATTCGAG 3480**

****************** ************************** ******** *********

**GQ918273.1 TTTTAGTAACAGCTACAGATGTTCTTCATTCTTGAACAGTTCCTTCTTTAGGGGTAAAGG 3540**

**mtAD TTTTAGTAACAGCTACAGATGTTCTTCATTCTTGAACAGTTCCTTCTTTAGGGGTAAAGG 3540**

**GQ918272.1 TTTTAGTAACAGCTACAGATGTTCTTCATTCTTGAACAGTTCCTTCTTTAGGGGTAAAGG 3540**

****************************************************************

**GQ918273.1 TAGATGCAACACCAGGCCGACTAAATCAACTTAATTTTTTAATTAATCGACCAGGATTGT 3600**

**mtAD TAGATGCAACACCAGGCCGACTAAATCAACTTAATTTTTTAATTAATCGACCAGGATTAT 3600**

**GQ918272.1 TAGATGCAACACCAGGCCGACTAAATCAACTTAATTTTTTAATCAATCGACCAGGATTAT 3600**

********************************************* ************** ***

**GQ918273.1 TTTTTGGTCAATGTTCTGAAATTTGTGGAGCTAATCATAGATTTATACCAATTGTAATTG 3660**

**mtAD TTTTTGGTCAATGTTCTGAAATTTGTGGAGCTAATCATAGATTTATACCAATTGTAATTG 3660**

**GQ918272.1 TTTTTGGTCAATGTTCAGAAATTTGTGGAGCTAATCATAGATTTATACCAATTGTAATTG 3660**

****************** *********************************************

**GQ918273.1 AAAGTATTCCTATAAATTTTTTTATTAAATGAATTACTTCTATAACTAATTCATTAGATG 3720**

**mtAD AAAGTATTCCTATAAATTTTTTTATTAAATGAATTACTTCTATAACTAATTCATTAGATG 3720**

**GQ918272.1 AAAGTATCCCTATAAATTTTTTTATTAAATGAATTACTTCTATAACTAATTCATTAGATG 3720**

********* ******************************************************

**GQ918273.1 ACTGAAAGCAAGTAATGATCTCTTAAATCATATTATAGTAAATTAGCACTTACTTCTAAT 3780**

**mtAD ACTGAAAGCAAGTAATGATCTCTTAAATCATATTATAGTAAATTAGCACTTACTTCTAAT 3780**

**GQ918272.1 ACTGAAAGCAAGTAATGATCTCTTAAATCATATTATAGTAAATTAGCACTTACTTCTAAT 3780**

****************************************************************

**GQ918273.1 GATATTTAACTAAAAAATTAGTTTCATAAAAACCTTAGTTTGTCAGACTAAAAAAATTAG 3840**

**mtAD GATATTTAACTAAAAAATTAGTTTCATAAAAACCTTAGTTTGTCAAACTAAAAAAATTAG 3840**

**GQ918272.1 GATACTTAACTAAAAAATTAGTTTCATAAAAACCTTAGTTTGTCAAACTAAAAAAATTAG 3840**

****** **************************************** ****************

**GQ918273.1 TTAAATCTAATATTTTTTAATCCCACAAATAGCTCCAATTAATTGGTTAATTTTATTCTT 3900**

**mtAD TTAAATCTAATATTTTTTAATCCCACAAATAGCTCCAATTAATTGGTTAATTTTATTCTT 3900**

**GQ918272.1 TTAAATCTAATATTTTTTAATCCCACAAATAGCTCCAATTAATTGGTTAATTTTATTTTT 3900**

*********************************************************** ****

**GQ918273.1 TGTTTTTTCAATTACATTAGTAATTTTTAATATTTTAAATTACTTTTGTTTTTTTTATAC 3960**

**mtAD TGTTTTTTCAATTACATTAGTAATTTTTAATATTTTAAATTACTTTTGTTTTTTTTATAC 3960**

**GQ918272.1 TGTTTTTTCAATTACATTAGTAATTTTTAATATTTTAAATTACTTTTGTTTTTTTTATAC 3960**

****************************************************************

**GQ918273.1 TCCATTAAAAACATCTCAACATCTTAATATTAAATTTAATAAACTTAATTGAAAATGATA 4020**

**mtAD TCCATTAAAAACATCTCAACATCTTAATATTAAATTTAATAAACTTAATTGAAAATGATA 4020**

**GQ918272.1 TCCATTAAAAACATCTCAACATCTTAATATTAAATTTAATAAGCTTAATTGAAAATGATA 4020**

******************************************** *******************

**GQ918273.1 ACAAACTTATTCTCTGTTTTCGATCCTTCAACAACTATTTTAAATTTGTCTTTAAACTGA 4080**

**mtAD ACAAACTTATTCTCTGTTTTCGATCCTTCAACAACTATTTTAAATTTGTCTTTAAACTGA 4080**

**GQ918272.1 ACAAACTTATTCTCTGTTTTCGATCCTTCAACAACTATTTTAAATTTGTCTTTAAACTGA 4080**

****************************************************************

**GQ918273.1 TTAAGAACATTTTTAGGATTATTTTTAATCCCTGCTTCTTTCTGATTATTACCTAATCGA 4140**

**mtAD TTAAGAACATTTTTAGGATTATTTTTAATCCCTGCTTCTTTCTGATTATTACCTAATCGA 4140**

**GQ918272.1 TTAAGTACATTTTTAGGATTATTTTTAATCCCTGCTTCTTTCTGATTATTACCTAATCGA 4140**

******* ********************************************************

**GQ918273.1 TTCCAAGTAGTTTGAAATAAAATTTTATTAACATTACATAATGAATTCAAAACTTTATTA 4200**

**mtAD TTCCAAGTAGTTTGAAATAAAATTTTATTAACATTACATAATGAATTCAAAACTTTATTA 4200**

**GQ918272.1 TTCCAAATAGTTTGAAATAAAATTTTATTAACATTACATAATGAATTCAAAACTTTATTA 4200**

******** *******************************************************

**GQ918273.1 GGACCTTCAGGACATAATGGAAGAACATTAATGTTTATTTCTTTATTTTCTTTAATTATA 4260**

**mtAD GGACCTTCAGGACATAATGGAAGAACATTAATGTTTATTTCTTTATTTTCTTTAATTATA 4260**

**GQ918272.1 GGACCTTCAGGACATAATGGAAGAACATTAATGTTTATTTCTTTATTTTCTTTAATTATA 4260**

****************************************************************

**GQ918273.1 TTTAATAATTTTTTAGGATTATTTCCTTATATTTTTACAAGCACTAGTCATTTAACTTTA 4320**

**mtAD TTTAATAATTTTTTAGGATTATTTCCTTATATTTTTACAAGCACTAGTCATTTAACTTTA 4320**

**GQ918272.1 TTTAATAATTTTTTAGGATTATTCCCTTATATTTTTACAAGCACTAGTCATTTAACTTTA 4320**

************************* **************************************

**GQ918273.1 ACTTTAGCCTTAGCATTCCCATTATGATTAAGTTTTATATTATATGGATGAATTAATCAT 4380**

**mtAD ACTTTAGCCTTAGCATTCCCATTATGATTAAGTTTTATATTATATGGATGAATTAATCAT 4380**

**GQ918272.1 ACTTTAGCCTTAGCATTCCCATTATGATTAAGTTTTATATTATATGGATGAATTAATCAT 4380**

****************************************************************

**GQ918273.1 ACACAACATATATTTGCTCATTTAGTTCCTCAAGGAACTCCTGCAGTTTTAATGCCATTT 4440**

**mtAD ACACAACATATATTTGCTCATTTAGTTCCTCAAGGAACTCCTGCAGTTTTAATGCCATTT 4440**

**GQ918272.1 ACACAACATATATTTGCTCATTTAGTTCCTCAAGGAACTCCTGCAGTTTTAATACCATTT 4440**

******************************************************* ********

**GQ918273.1 ATGGTATGCATTGAAACAATTAGAAATGTAATTCGACCAGGAACTTTAGCAGTGCGATTA 4500**

**mtAD ATGGTATGCATTGAAACAATTAGAAATGTAATTCGACCAGGAACTTTAGCAGTGCGATTA 4500**

**GQ918272.1 ATGGTATGCATTGAAACAATTAGAAATGTAATTCGACCAGGAACTTTAGCAGTACGATTA 4500**

******************************************************* ********

**GQ918273.1 ACAGCAAATATAATTGCTGGACATTTGCTAATAACTCTCTTAGGGAATACTGGACCAATG 4560**

**mtAD ACAGCAAATATAATTGCTGGACATTTGCTAATAACTCTCTTAGGGAATACTGGACCAATG 4560**

**GQ918272.1 ACAGCAAATATAATTGCTGGACATTTGCTAATAACTCTTTTAGGAAATACTGGACCAATA 4560**

**************************************** ***** ****************

**GQ918273.1 TCAGCTAATTACTTAATTTTATCATTAATTTTAATAACACAAATTGCATTATTAGTATTA 4620**

**mtAD TCAGCTAATTACTTAATTTTATCATTAATTTTAATAACACAAATTGCATTATTAGTATTA 4620**

**GQ918272.1 TCAGCTAATTACTTAATTTTATCATTAATTTTAACAACACAAATTGCATTATTAGTATTA 4620**

************************************ ***************************

**GQ918273.1 GAATCAGCTGTAGCAATTATTCAATCTTATGTTTTCGCAGTATTAAGAACTCTTTACTCA 4680**

**mtAD GAATCAGCTGTAGCAATTATTCAATCTTATGTTTTCGCAGTATTAAGAACTCTTTACTCA 4680**

**GQ918272.1 GAATCAGCTGTAGCAATTATTCAATCTTATGTTTTCGCAGTATTAAGAACTCTTTACTCA 4680**

****************************************************************

**GQ918273.1 AGTGAAGTTAATTAATGTCAACACATGCAAATCACCCCTTTCATTTAGTTGATTATAGCC 4740**

**mtAD AGTGAAGTTAATTAATGTCAACACATGCAAATCACCCCTTTCATTTAGTTGATTATAGCC 4740**

**GQ918272.1 AGTGAAGTTAATTAATGTCAACACATGCAAATCACCCCTTTCATTTAGTTGATTATAGCC 4740**

****************************************************************

**GQ918273.1 CATGACCATTAACTGGAGCAATTGGAGCTATAACAACTGTTTCAGGATTAGTCCAATGAT 4800**

**mtAD CATGACCATTAACTGGAGCAATTGGAGCTATAACAACTGTTTCAGGATTAGTCCAATGAT 4800**

**GQ918272.1 CATGACCACTAACTGGAGCAATTGGAGCTATAACAACTGTTTCAGGATTAGTCCAATGAT 4800**

********** *****************************************************

**GQ918273.1 TTCATCAATATACAATAACTTTATTTATTCTAGGGAATATTATCACAATTTTAACAATAT 4860**

**mtAD TTCATCAATATACAATAACTTTATTTATTCTAGGGAATATTATCACAATTTTAACAATAT 4860**

**GQ918272.1 TTCATCAATACACAATAACTTTATTTATTCTAGGGAATATTATCACAATTTTAACAATAT 4860**

************ ***************************************************

**GQ918273.1 ATCAATGATGACGAGATATTTCTCGAGAAGGAACTTTTCAAGGATTACATACTTACCCAG 4920**

**mtAD ATCAATGATGACGAGATATTTCTCGAGAAGGAACTTTTCAAGGATTACATACTTACCCAG 4920**

**GQ918272.1 ATCAATGATGACGAGATATTTCTCGAGAAGGAACTTTTCAAGGATTACATACTTATCCAG 4920**

********************************************************* ******

**GQ918273.1 TAACAATTGGATTACGATGAGGAATAATTTTATTTATTGTATCAGAAGTATTTTTTTTTA 4980**

**mtAD TAACAATTGGATTACGATGAGGAATAATTTTATTTATTGTATCAGAAGTATTTTTTTTTA 4980**

**GQ918272.1 TAACAATTGGATTACGATGAGGAATAATTTTATTTATTGTATCAGAAGTATTTTTTTTTA 4980**

****************************************************************

**GQ918273.1 TTTCTTTTTTTTGAGCATTTTTTCATAGTAGTTTATCCCCAACTATTGAATTAGGAATAA 5040**

**mtAD TTTCTTTTTTTTGAGCATTTTTTCATAGTAGTTTATCCCCAACTATTGAATTAGGAATAA 5040**

**GQ918272.1 TTTCTTTTTTTTGAGCATTTTTTCATAGTAGTTTATCCCCAACTATTGAATTAGGAATAA 5040**

****************************************************************

**GQ918273.1 CATGACCTCCAGTAGGAGTAATTGCTTTTAATCCATTTCAAATTCCTTTATTAAATACTG 5100**

**mtAD CATGACCTCCAGTAGGAGTAATTGCTTTTAATCCATTTCAAATTCCTTTATTAAATACTG 5100**

**GQ918272.1 CATGACCTCCAGTAGGAGTAATTGCTTTTAATCCATTTCAAATTCCTTTATTAAATACTG 5100**

****************************************************************

**GQ918273.1 CTATTTTATTGGCCTCAGGAGTAACAGTAACATGGGCTCATCATAGCTTAATAGAAGGTA 5160**

**mtAD CTATTTTATTGGCCTCAGGAGTAACAGTAACATGGGCTCATCATAGCTTAATAGAAGGTA 5160**

**GQ918272.1 CTATTTTATTAGCTTCAGGGGTGACAGTAACATGAGCTCATCATAGCTTAATAGAAAGTA 5160**

************ ** ***** ** *********** ********************* *****

**GQ918273.1 ATCATTCTCAAACAACACAAGGATTATTTTTTACTATTGTATTAGGAGTATATTTTTCAA 5220**

**mtAD ATCATTCTCAAACAACACAAGGATTATTTTTTACTATTGTATTAGGAGTATATTTTTCAA 5220**

**GQ918272.1 ATCATTCTCAAACAACACAAGCATTATTTTTTACTATTGTATTAGGAGTATATTTTTCAA 5220**

*********************** ****************************************

**GQ918273.1 TTCTTCAAGCATATGAATACATCGAAGCACCATTTACTATTGCTGATGCAGTATACGGAT 5280**

**mtAD TTCTTCAAGCATATGAATACATCGAAGCACCATTTACTATTGCTGATGCAGTATACGGAT 5280**

**GQ918272.1 TTCTTCAAGCATATGAATACATCGAAGCACCATTTACTATTGCTGATGCAGTATATGGAT 5280**

********************************************************* ******

**GQ918273.1 CTACTTTTTATATAGCAACAGGATTCCATGGATTACATGTATTGATTGGAACAACTTTCT 5340**

**mtAD CTACTTTTTATATAGCAACAGGATTCCATGGATTACATGTATTGATTGGAACAACTTTCT 5340**

**GQ918272.1 CTACTTTTTATATAGCAACAGGATTCCATGGATTACATGTATTAATTGGAACAACTTTCT 5340**

********************************************* ******************

**GQ918273.1 TATTAATTTGTTTTTTACGTCACATTAATTTTCATTTTTCAAAAAATCATCATTTTGGAT 5400**

**mtAD TATTAATTTGTTTTTTACGTCACATTAATTTTCATTTTTCAAAAAATCATCATTTTGGAT 5400**

**GQ918272.1 TATTAATTTGTTTTTTACGTCACATTAATTTTCATTTTTCAAAAAATCATCATTTTGGAT 5400**

****************************************************************

**GQ918273.1 TTGAAGCTGCAGCATGATACTGACATTTTGTAGATGTTGTATGATTATTTTTATATATTT 5460**

**mtAD TTGAAGCTGCAGCATGATACTGACATTTTGTAGATGTTGTATGATTATTTTTATATATTT 5460**

**GQ918272.1 TTGAAGCTGCAGCATGATACTGACATTTTGTAGATGTTGTATGATTATTTTTATATATTT 5460**

****************************************************************

**GQ918273.1 CAATTTACTGATGAGGTAGATATTTATAAAGTATATATTTGTATATGTGACTTCCAATCA 5520**

**mtAD CAATTTACTGATGAGGTAGATATTTATAAAGTATATATTTGTATATGTGACTTCCAATCA 5520**

**GQ918272.1 CAATTTACTGATGAGGTAGATATTTATAAAGTATATATTTGTATATGTGACTTCCAATCA 5520**

****************************************************************

**GQ918273.1 CAAGGACTAAATAATTTTAGTATAAATAATATTAATATTATCGACAATAACTTTAATTAT 5580**

**mtAD CAAGGACTAAATAATTTTAGTATAAATAATATTAATATTATCGACAATAACTTTAATTAT 5580**

**GQ918272.1 CAAGGACTAAATAATTTTAGTATAAATAATATTAATATTATCAATAATAACTTTAATTAT 5580**

******************************************** * *****************

**GQ918273.1 TATAATCATCACTATTGTAGTAATAATTCTTGCTACATTATTATCAAAAAAAACTTTAAC 5640**

**mtAD TATAATCATCACTATTGTAGTAATAATTCTTGCTACATTATTATCAAAAAAAACTTTAAC 5640**

**GQ918272.1 TATAATCATCACTATTGTAGTAATAATACTTGCTACACTATTATCAAAAAAAACTTTAAC 5640**

***************************** ********* ************************

**GQ918273.1 AGATCGAGAAAAATGTTCACCATTTGAATGTGGATTTGATCCAATAAATTCATCTCGTTT 5700**

**mtAD AGATCGAGAAAAATGTTCACCATTTGAATGTGGATTTGATCCAATAAATTCATCTCGTTT 5700**

**GQ918272.1 AGATCGAGAAAAATGTTCACCATTTGAATGTGGATTTGATCCAATAAATTCATCTCGTTT 5700**

****************************************************************

**GQ918273.1 ACCATTTTCTTTACGATTTTTTTTAATTGCAATTATTTTTTTAATTTTTGATGTAGAAAT 5760**

**mtAD ACCATTTTCTTTACGATTTTTTTTAATTGCAATTATTTTTTTAATTTTGTATGTAGAAAT 5760**

**GQ918272.1 ACCATTTTCTTTACGATTTTTTTTAATTGCAATTATTTTTTTAATTTTTGATGTAGAAAT 5760**

************************************************** ************

**GQ918273.1 TGCTTTATTACTACCAATAATCATAATTATTAAATCTTCAAATTTAATTAATTGAACTAT 5820**

**mtAD TGCTTTATTACTACCAATAATCATAATTATTAAATCTTCAAATTTAATTAATTGAACTAT 5820**

**GQ918272.1 TGCTTTATTACTACCAATAATCATAATTATTAAATCTTCAAATTTAATTAATTGAACTAT 5820**

****************************************************************

**GQ918273.1 TACTAGATTATTCTTTATTTTTATTTTAATTGTTGGATTATACCATGAATGAAATCAAGG 5880**

**mtAD TACTAGATTATTCTTTATTTTTATTTTAATTGTTGGATTATACCATGAATGAAATCAAGG 5880**

**GQ918272.1 TACTAGATTATTCTTTATTTTTATTTTAATTGTTGGATTATACCATGAATGAAATCAAGG 5880**

****************************************************************

**GQ918273.1 AGCATTAGAATGAAATGAATAAATATGAAGCGATTTATTGCAATTAGTTTCGGCCTAATC 5940**

**mtAD AGCATTAGAATGAAATGAATAAATATGAAGCGATTTATTGCAATTAGTTTCGGCCTAATC 5940**

**GQ918272.1 AGCATTAGAATGAAATGAATAAATATGAAGCGATTTATTGCAATTAGTTTCGGCCTAATC 5940**

****************************************************************

**GQ918273.1 TTAGGTGAAATTCACCCATATTTTAGGGTAATAGTTAACTATAACATTTAATTTGCATTT 6000**

**mtAD TTAGGTGAAATTCACCCATATTTTAGGGTAATAGTTAACTATAACATTTAATTTGCATTT 6000**

**GQ918272.1 TTAGGTGAAATTCACCCATATTTTAGGGTAATAGTTAACTATAACATTTAATTTGCATTT 6000**

****************************************************************

**GQ918273.1 AAAAAGTATTGAATTATTCAATTTACCTTATTAATTGAAACCAAAAAGAGGTATATCACT 6060**

**mtAD AAAAAGTATTGAATTATTCAATTTACCTTATTAATTGAAACCAAAAAGAGGTATATCACT 6060**

**GQ918272.1 AAAAAGTATTGAATTATTCAATTTACCTTATTAATTGAAACCAAAAAGAGGTATATCACT 6060**

****************************************************************

**GQ918273.1 GTTAATGATAAAATTGAATTTTTATAATTCCAATTAAAGAAATATAAATGGAATTAAACC 6120**

**mtAD GTTAATGATAAAATTGAATTTTTATAATTCCAATTAAAGAAATATAAATGGAATTAAACC 6120**

**GQ918272.1 GTTAATGATAAAATTGAATTTTTATAATTCCAATTAAAGAAATATAAATGGAATTAAACC 6120**

****************************************************************

**GQ918273.1 ATTAAAGTTAAAAGTTAGCAGCTTTTACTTGATCAACATATTTCAATTTATATAGTTTAA 6180**

**mtAD ATTAAAGTTAAAAGTTAGCAGCTTTTACTTGATCAACATATTTCAATTTATATAGTTTAA 6180**

**GQ918272.1 ATTAAAGTTAAAAGTTAGCAGCTTTTACTTGATCAACATATTTCAATTTATATAGTTTAA 6180**

****************************************************************

**GQ918273.1 ACAAAACATTACATTTTCAATGTAAAAATAAAAATTTATTTTTTATAAATATCTAAAGAT 6240**

**mtAD ACAAAACATTACATTTTCAATGTAAAAATAAAAATTTATTTTTTATAAATATCTAAAGAT 6240**

**GQ918272.1 ACAAAACATTACATTTTCAATGTAAAAATAAAAATTTATTTTTTATAAATATCTAAAGAT 6240**

****************************************************************

**GQ918273.1 TAAAATAATCACCCTAACATCTTCAGTGTCATGCTCTAAATTTAAGCTATTTAAATAATT 6300**

**mtAD TAAAATAATCACCCTAACATCTTCAGTGTCATGCTCTAAATTTAAGCTATTTAAATAATT 6300**

**GQ918272.1 TAAAATAATCACCCTAACATCTTCAGTGTCATGCTCTAAATTTAAGCTATTTAAATAATT 6300**

****************************************************************

**GQ918273.1 AATAAAACTAATTATTATTAATAAAATAATAAATCATAATATATATCTTAATAAATAAAT 6360**

**mtAD AATAAAACTAATTATTATTAATAAAATAATAAATCATAATATATATCTTAATAAATAAAT 6360**

**GQ918272.1 AATAAAACTAATTATTATTAATAAAATAATAAATCATAATATATATCTTAATAAATAAAT 6360**

****************************************************************

**GQ918273.1 TTTTAAACTATTATTTTGAAATTCTTGTAAGTATAAAGAATAATTTTTTAATTGATTATA 6420**

**mtAD TTTTAAACTATTATTTTGAAATTCTTGTAAATATAAAGAATAATTTTTTAATTGATTATA 6420**

**GQ918272.1 TTTTAAACTATTATTTTGAAATTCTTGTAAATATAAAGAATAATTTTTTAATTGATTATA 6420**

******************************** *******************************

**GQ918273.1 TAATATTTGACCTCCAAAAAACTCTCTTCAACCTTGATCAAAACTTTTAAATGAATATAG 6480**

**mtAD TAATATTTGACCCCCAAAAAACTCTCTTCAACCTTGATCAAAACTTTTAAATGAATATAA 6480**

**GQ918272.1 TAATATTTGACCTCCAAAAAACTCTCTTCAACCTTGATCAAAACTTTTAAATGAATATAA 6480**

************** ************************************************

**GQ918273.1 TCCTAATTTTAATGGATAATTAATAATACCTACAGTAGAAAGAACAGGTATAAATCATAT 6540**

**mtAD TCCTAATTTTAATGGATAATTAATAATACCTACAGTAGAAAGAACAGGTATAAATCATAT 6540**

**GQ918272.1 TCCTAATTTTAATGGATAATTAATAATACCTACAGTAGAAAGAACAGGTATAAATCATAT 6540**

****************************************************************

**GQ918273.1 TGAACCAGCAAAATAAACAAAATTATAATAATACAACCCCTTATTAATAAAAAATAACTT 6600**

**mtAD TGAACCAGCAAAATAAGCAAAATTATAATAATACAACCCCTTATTAATAAAAAATAACTT 6600**

**GQ918272.1 TGAACCAGCAAAATAAATAAAATTATAATAATACAACCCCTTATTAATAAAAAATAACTT 6600**

****************** ********************************************

**GQ918273.1 AACATTAGTTAATAAATACCCTATTCTTCCTCCTAATAAACATACAATTAATGTTAAAAT 6660**

**mtAD AACATTAGTTAATAAATACCCTATTCTTCCTCCTAATAAACATACAATTAATGTTAAAAT 6660**

**GQ918272.1 AACATTAGTTAATAAATATCCTATTCTTCCTCCTAATAAACATACAATTAATGTTAAAAT 6660**

******************** *******************************************

**GQ918273.1 TTTTATATCAAAAGGTAAACAAATTATTGAAGGATTTAAAAATATTAATCATCTTAATAT 6720**

**mtAD TTTTATATCAAAAGGTAAACAAATTATTGAAGGATTTAAAAATATTAATCATCTTAATAT 6720**

**GQ918272.1 TTTTATATCAAAAGGTAAACAAATTATTGAAGGATTTAAAAATATTAATCATCTTAATAT 6720**

****************************************************************

**GQ918273.1 TCTCCCCCCAATAATTGCTATAATTATTAAAAAAAAAATACTAAATAATATAATTATACT 6780**

**mtAD TCTCCCCCCAATAATTGCTATAATTATTAAAAAAAAAATACTAAATAATATAATTATACT 6780**

**GQ918272.1 TCTCCCCCCAATAATTGCTATAATTATTAAAAAAAAAATACTAAATAATATAATTATACT 6780**

****************************************************************

**GQ918273.1 TTTATCATTTAAAGGATGTAAAGATCTTCTATTAAATTCTCCAGTTATTGAGTAATAAAC 6840**

**mtAD TTTATCATTTAAAGGATGTAAAGATCTTCTATTAAATTCTCCAGTTATTGAATAATAAAC 6840**

**GQ918272.1 TTTATCATTTAAAGGATGTAAAGATCTTCTATTAAATTCTCCAGTTATTGAGTAATAAAC 6840**

***************************************************** **********

**GQ918273.1 TAAACGAAATGAATAACATACTGTTAACCCTGTACTAAAAAAAAAAAGAAAAAAAGAAAA 6900**

**mtAD TAAACGAAATGAATAACATACTGTTAACCCTGTACTAAAAAAAAAAAGAAAAAAAGAAAA 6900**

**GQ918272.1 TAAACGAAATGAATAGCATACTGTTAACCCCGTACTAAAAAAAAAAAGAAAAAAAGAAAA 6900**

***************** ************** *******************************

**GQ918273.1 ACTATTCACATATGATAATATTACTATTTCTAAAATTAAATCCTTAGAATAAAATCCAGC 6960**

**mtAD ACTATTCACATATGATAATATTACTATTTCTAAAATTAAATCCTTAGAATAAAATCCAGC 6960**

**GQ918272.1 ACTATTCACATATGATAATATTACTATTTCTAAAATTAAATCCTTAGAATAAAATCCAGC 6960**

****************************************************************

**GQ918273.1 TAAAAAAGGTATTCCACATAAAGCTAGATTAGCAATATTAAAACAACTACAAGTTAAAGG 7020**

**mtAD TAAAAAAGGTATTCCACATAAAGCTAGATTAGCAATATTAAAACAACTACAAGTTAAAGG 7020**

**GQ918272.1 TAAAAAAGGTATTCCACATAAAGCTAGATTAGCAATATTAAAACAACTACACGTTAAAGG 7020**

***************************************************** **********

**GQ918273.1 CATTCTTATATTTAATCTTCCTATTATACGAATATCTTGAGAATTTTTTATATTATGAAT 7080**

**mtAD CATTCTTATATTTAATCTTCCTATTATACGAATATCTTGAGAATTTTTTATATTATGAAT 7080**

**GQ918272.1 CATTCTTATATTTAATCTTCCTATTATACGAATATCTTGAGAATTTTTTATATTATGAAT 7080**

****************************************************************

**GQ918273.1 AATAGAACCAGCACATATAAACAAAAGAGCTTTAAATAAAGCATGGGTTAATAAATGAAA 7140**

**mtAD AATAGAACCAGCACATATAAACAAAAGAGCTTTAAATAAAGCATGGGTTAATAAATGAAA 7140**

**GQ918272.1 AATAGAACCAGCACATATAAACAAAAGAGCCTTGAATAAAGCATGGGTTAATAAATGAAA 7140**

******************************** ** ****************************

**GQ918273.1 AAAGGCTAATTTATAAAATCCTATTGATAAAATACTTATTATCAATCCTAATTGACTTAA 7200**

**mtAD AAAGGCTAATTTATAAAATCCTATTGATAAAATACTTATTATCAATCCTAATTGACTTAA 7200**

**GQ918272.1 AAAGGCTAATTTATAAAATCCTATTGATAAAATACTTATTATCAATCCTAATTGACTTAA 7200**

****************************************************************

**GQ918273.1 AGTAGATAAGGCAATAATTTTTTTTAAATCAAACTCAAAATTTGCTCCTAATCCAGCTAT 7260**

**mtAD AGTAGATAAGGCAATAATTTTTTTTAAATCAAACTCAAAATTTGCTCCTAATCCAGCTAT 7260**

**GQ918272.1 AGTAGATAAAGCAATAATTTTTTTTAAATCAAATTCAAAATTTGCTCCTAATCCAGCTAT 7260**

*********** *********************** ****************************

**GQ918273.1 AAATATAGTTAATCCTGATACTAATAATAAAAATTGACCTATTCATAAATCTATTAATAA 7320**

**mtAD AAATATAGTTAATCCTGATACTAATAATAAAAATTGACCTATTCATAAATCTGTTAATAA 7320**

**GQ918272.1 AAATATAGTTAGTCCTGATACTAATAATAAAAATTGACCTATTCATAAATCTGTTAATAA 7320**

************* **************************************** *********

**GQ918273.1 TACATTAAATCGAATTAATAAATAAACTCCTGCAGTAACTAAAGTTGATGAATGAACTAA 7380**

**mtAD TACATTAAATCGAATTAATAAATAAACTCCTGCAGTAACTAAAGTTGATGAATGAACTAA 7380**

**GQ918272.1 TACATTAAATCGAATTAATAAATAAACTCCTGCAGTAACTAAAGTTGATGAATGAACTAA 7380**

****************************************************************

**GQ918273.1 AGCAGAGACCGGAGTAGGGGCTGCTATAGCAGCAGGTAATCATGATGAAAAAGGAATTTG 7440**

**mtAD AGCAGAGACCGGAGTAGGGGCTGCTATAGCAGCAGGTAATCATGATGAAAAAGGAATTTG 7440**

**GQ918272.1 AGCAGAGACAGGAGTAGGAGCTGCTATAGCAGCAGGTAATCATGATGAAAAAGGAATTTG 7440**

*********** ******** *******************************************

**GQ918273.1 AGCTCTTTTAGTTATAGCAGCTAACATTACTAATGCTCCAATAATTATTATTTCAAACTT 7500**

**mtAD AGCTCTTTTAGTTATAGCGGCTAACATTACTAATGCTCCAATAATTATTATTTCAAACTT 7500**

**GQ918272.1 AGCTCTTTTAGTTATAGCGGCTAACATTACTAATGCTCCAATAATTATTATTTCAAACTT 7500**

******************** *******************************************

**GQ918273.1 AGTTGATAATATATCTAAATAAAAAATGTAATTTCAACTTCCATAATTTAATATTCAAGC 7560**

**mtAD AGTTGATAATATATCTAAATAAAAAATGTAATTTCAACTTCCATAATTTAATATTCAAGC 7560**

**GQ918272.1 AGTTGATAATATATCTAAATAAAAAATGTAATTTCAACTTCCATAATTTAATATTCAAGC 7560**

****************************************************************

**GQ918273.1 AATAGCTAATAATAAAGCAACATCTCCTACTCGATTTGATAAAGCAGTTAATATCCCAGC 7620**

**mtAD AATAGCTAATAATAAAGCAACATCTCCTACTCGATTTGATAAAGCAGTTAATATCCCAGC 7620**

**GQ918272.1 AATGGCTAATAATAAAGCAACATCACCTACTCGATTTGATAAAGCAGTTAATATCCCAGC 7620**

***** ******************** *************************************

**GQ918273.1 GTTATAAGATTTAACATTTTGAAAATAAATAACTAAACAATAAGAAACAAGTCCAAGTCC 7680**

**mtAD GTTATAAGATTTAACATTTTGAAAATAAATAACTAAACAATAAGAAACAAGTCCAAGTCC 7680**

**GQ918272.1 GTTATAAGATTTAACATTTTGAAAATAAATAACTAAACAATAAGAAACAAGTCCAAGTCC 7680**

****************************************************************

**GQ918273.1 ATCTCATCCTAATAAAATTCTAATTAAATTTGGACTAATAATTAACAATATTATAGATAT 7740**

**mtAD ATCTCATCCTAATAAAATTCTAATTAAATTTGGACTAATAATTAACAATATTATAGATAT 7740**

**GQ918272.1 ATCTCATCCTAATAAAATTCTAATTAAATTTGGACTAATAATTAATAATATTATAGATAT 7740**

*********************************************** ****************

**GQ918273.1 AACAAATATTAAAACTAATAAAATAAATCGATTAATGTTATAATCTTCCTCTATATATTG 7800**

**mtAD AACAAATATTAAAACTAATAAAATAAATCGATTAATGTTATAATCTTCCTCTATATATTG 7800**

**GQ918272.1 AACAAATATTAAAACTAATAAAATAAATCGATTAATGTTATAATCTTCTTCTATATATTG 7800**

************************************************** *************

**GQ918273.1 ATTACTATAAAAAATAACTAAAGAAGAAATCAATAAAACAAAAGATATAAATATTAATCT 7860**

**mtAD ATTACTATAAAAAATAACTAAAGAAGAAATCAATAAAACAAAAGATATAAATATTAATCT 7860**

**GQ918272.1 ATTACTATAAAAAATAACTAAAGAAGAAATCAATAAAACAAAAGATATAAATATTAATCT 7860**

****************************************************************

**GQ918273.1 TATTCAATCAAATAAAAAAGTTATAACAATAGATATTGATTGCAAAGATAAAACTTCTCA 7920**

**mtAD TATTCAATCAAATAAAAAAGTTATAACAATAGATATTGATTGCAAAGATAAAACTTCTCA 7920**

**GQ918272.1 TATTCAATCAAATAAAAAAGTTATAACAATAGATATTGATTGCAAAGATAAAACTTCTCA 7920**

****************************************************************

**GQ918273.1 TTCAATAAAATAAACTAAATCTTTTAGAATAAACTTTATTCTTAATAAAAATAAACTAAT 7980**

**mtAD TTCAATAAAATAAACTAAATCTTTTAGAATAAACTTTATTCTTAATAAAAATAAACTAAT 7980**

**GQ918272.1 TTCAATAAAATAAACTAAATCTTTTAGAATAAACTTTATTCTTAATAAAAATAAACTAAT 7980**

****************************************************************

**GQ918273.1 TCTAATAAAAATTAAAAAATAAAAACTTGTTTTACAATAATTTACTAAGCTATTCACGAT 8040**

**mtAD TCTAATAAAAATTAAAAAATAAAAACTTGTTTTACAATAATTTACTAAGCTATTCACGAT 8040**

**GQ918272.1 TCTAATAAAAATTAAAAAATAAAAACTTGTTTTACAATAATTTACTAAGCTATTCACGAT 8040**

****************************************************************

**GQ918273.1 CTAAAATGAATTAATCATATCATTGACACCACAAATCAATATTTTTTTTAAACTATTTAA 8100**

**mtAD CTAAAATGAATTAATCATATCATTGACACCACAAATCAATATTTTTTTTAAACTATTTAA 8100**

**GQ918272.1 CTAAAATGAATTAATCATATCATTGACACCACAAATCAATATTTTTTTTAAACTATTTAA 8100**

****************************************************************

**GQ918273.1 ATAAATTCATAATATACAAAAACTTCTCTTTAAAATTAATAAATTTAAAGGAAGTCAATG 8160**

**mtAD ATAAATTCATAATATACAAAAACTTCTCTTTAAAATTAATAAATTTAAAGGAAGTCAATG 8160**

**GQ918272.1 ATAAATTCATAATATACAAAAACTTCTCTTTAAAATTAATAAATTTAAAGGAAGTCAATG 8160**

****************************************************************

**GQ918273.1 TAATATTAATAAAGAAAATTCTCGAACAGATCCTACTGAAAAAAAATGAACTCCAGAATA 8220**

**mtAD TAATATTAATAAAGAAAATTCTCGAACAGATCCTACTGAAAAAAAATGAACTCCAGAATA 8220**

**GQ918272.1 TAACATTAATAAAGAAAATTCTCGAACAGATCCTACTGAAAAAAAATGAACTCCAGAATA 8220**

***** **********************************************************

**GQ918273.1 AATTTTTCCATGTTGACTATAAGCAAATAAATATAAAGAATAAGCAGCTCTAAAAAAAGA 8280**

**mtAD AATTTTTCCATGTTGACTATAAGCAAATAAATATAAAGAATAAGCAGCTCTAAAAAAAGA 8280**

**GQ918272.1 AATTTTTCCATGTTGACTATAAGCAAATAAATATAAAGAATAAGCAGCTCTAAAAAAAGA 8280**

****************************************************************

**GQ918273.1 TAAAAAAGTTAACATAATCATAGTAATTCAAGACCACCCAACAATTCTATTTAATAAAGA 8340**

**mtAD TAAAAAAGTTAACATAATCATAGTAATTCAAGACCACCCAACAATTCTATTTAATAAAGA 8340**

**GQ918272.1 TAAAAAAGTTAACATAATCATAGTAATTCAAGACCACCCAACAATTCTATTTAATAAAGA 8340**

****************************************************************

**GQ918273.1 AATTTCACCTAATAAATTAAGAGTAGGAGGTGCTGCCATATTACCTGAACATAATAAAAA 8400**

**mtAD AATTTCACCTAATAAATTAAGAGTAGGAGGTGCTGCCATATTACCTGAACATAATAAAAA 8400**

**GQ918272.1 AATTTCACCTAATAAATTAAGAGAAGGAGGTGCTGCCATATTACCTGAACATAATAAAAA 8400**

************************* **************************************

**GQ918273.1 TCATCATAAACTTAATGTAGGTATAAAATTTAGAAGCCCCTTATTAATTAATAAACTTCG 8460**

**mtAD TCATCATAAACTTAATGTAGGTATAAAATTTAGAAGCCCCTTATTAATTAATAAACTTCG 8460**

**GQ918272.1 TCATCATAAACTTAATGTAGGCATAAAATTTAGAAGTCCCTTATTAATTAATAAACTTCG 8460**

*********************** ************** *************************

**GQ918273.1 TCTTCCTATTCGTTCATATGAAATATTTGCTAAACAAAATAATCCAGATGAACATAACCC 8520**

**mtAD TCTTCCTATTCGTTCATATGAAATATTTGCTAAACAAAATAATCCAGATGAACATAACCC 8520**

**GQ918272.1 TCTTCCTATTCGTTCATACGAAATATTTGCTAAACAAAATAATCCAGATGAACATAATCC 8520**

******************** ************************************** ****

**GQ918273.1 ATGAGCAATTATTAATGCGTATGAACCAGTTAATCCTCAATAAGTTAAAGTTAATAAACC 8580**

**mtAD ATGAGCAATTATTAATGCGTATGAACCAGTTAATCCTCAATAAGTTAAAGTTAATAAACC 8580**

**GQ918272.1 ATGAGCGATTATTAATGCGTATGAACCAGTTAATCCTCAATAAGTTAAAGTTAATAAACC 8580**

******** *******************************************************

**GQ918273.1 TCTTAACACAATTCCTATATGTGCTACAGATGAATAAGCAATTAAAGCCTTTAAATCAGT 8640**

**mtAD TCTTAACACAATTCCTATATGTGCTACAGATGAATAAGCAATTAAAGCCTTTAAATCAGT 8640**

**GQ918272.1 TCTTAACACAATTCCTATATGTGCTACAGATGAATAAGCAATTAAAGCCTTTAAGTCAGT 8640**

******************************************************** *******

**GQ918273.1 CTGACGTAAACAAATTAATCTAATTAATACTCCTCCTACTAAACTAATACTAATTCATCA 8700**

**mtAD CTGACGTAAACAAATTAATCTAATTAATACTCCTCCTACTAAACTAATACTAATTCATCA 8700**

**GQ918272.1 TTGACGTAAACAAATTAATCTAATTAATACTCCTCCTACTAAACTAATACTAATTCATCA 8700**

***************************************************************

**GQ918273.1 ATAATTATATTTAACTCCTGATATTTGTAATAACGAAAATATTCGTAATAATCCATATCC 8760**

**mtAD ATAATTATATTTAACTCCTGATATTTGTAATAACGAAAATATTCGTAATAATCCATATCC 8760**

**GQ918272.1 ATAATTATATTTAACTCCTGATATTTGTAATAACGAAAATATTCGTAATAATCCATATCC 8760**

****************************************************************

**GQ918273.1 TCCTAATTTTAATAAAATACCTGCTAAAATCATAGATCCAGAAACAGGAGCTTCTACATG 8820**

**mtAD TCCTAATTTTAATAAAATACCTGCTAAAATCATAGATCCAGAAACAGGAGCTTCTACATG 8820**

**GQ918272.1 TCCTAATTTTAATAAAATACCTGCTAAAATCATAGATCCAGAAACAGGAGCTTCTACATG 8820**

****************************************************************

**GQ918273.1 AGCTTTGGGTAATCAAAGATGAACTAAAAATATTGGTATTTTTACTAAAAAAGCAAAAAT 8880**

**mtAD AGCTTTGGGTAATCAAAGATGAACTAAAAATATTGGTATTTTTACTAAAAAAGCAAAAAT 8880**

**GQ918272.1 AGCTTTTGGCAATCAAAGATGAACTAAAAATATTGGTATTTTTACTAAAAAAGCAAAAAT 8880**

******** ** ****************************************************

**GQ918273.1 TAACGAAAGATATAATAAATTTAAATCTATAAAAACATAATTTAATAACAAATTAAAAGA 8940**

**mtAD TAACGAAAGATATAATAAATTTAAATCTATAAAAACATAATTTAATAACAAATTAAAAGA 8940**

**GQ918272.1 TAACGAAAGATATAATAAATTTAAATCTATAAAAACATAATTTAACAACAAATTAAAAGA 8940**

*********************************************** ****************

**GQ918273.1 TAAAGTGTTATTAATTTCTAAAATATAAAAAATACCAATTAATAAAGGTAAAGAAGCTAA 9000**

**mtAD TAAAGTGTTATTAATTTCTAAAATATAAAAAATACCAATTAATAAAGGTAAAGAAGCTAA 9000**

**GQ918272.1 TAAAGTGTTATTAATTTCTAAAATATAAAAAATACCAATTAATAAAGGTAAAGAAGCTAA 9000**

****************************************************************

**GQ918273.1 TAAAGTATAAAATAATAAATAAACCCCTGCTTGCAATCGTTCAGGCTGATACCCTCATCC 9060**

**mtAD TAAAGTATAAAATAATAAATAAACCCCTGCTTGCAATCGTTCAGGCTGATACCCTCATCC 9060**

**GQ918272.1 TAAAGTATAAAATAATAAATAGACTCCTGCTTGCAATCGTTCAGGCTGATATCCTCATCC 9060**

*********************** ** ************************** **********

**GQ918273.1 TAAAATTAAAAATAAAGTAGGAATTAATCTAGCTTCAAAAAATAAATAAAATATAAAAAC 9120**

**mtAD TAAAATTAAAAATAAAGTAGGAATTAATCTAGCTTCAAAAAATAAATAAAATATAAAAAC 9120**

**GQ918272.1 TAAAATTAAAAATAAAGTAGGAATTAATCTAGCTTCAAAAAATAAATAAAATATAAAAAC 9120**

****************************************************************

**GQ918273.1 TCTCATTGAACTAAAAGTTAATACTAATATTATTAATAAAAACAAAATTATAAAAATAAA 9180**

**mtAD TCTCATTGAACTAAAAGTTAATACTAATATTATTAATAAAAACAAAATTATAAAAATAAA 9180**

**GQ918272.1 TCTCATTGAACTAAAAGTTAATACTAATATTATTAATAAAAACAAAATTATAAAAATAAA 9180**

****************************************************************

**GQ918273.1 TAAATTTTTATAATTATTATATAAATAAACTTTTTCACTTGCTATTAGTATTAATCCACA 9240**

**mtAD TAAATTTTTATAATTATTATATAAATAAACTTTTTCACTTGCTATTAGTATTAATCCACA 9240**

**GQ918272.1 TAAATTTTTATAATTATTATATAAATAAACTTTTTCACTTGCTATTAATATTAATCCACA 9240**

************************************************* **************

**GQ918273.1 AATTCAAAAACTCAATAAAATTAAACCAAACGAAATTATATCTAATCCAAAATAATAAGA 9300**

**mtAD AATTCAAAAACTCAATAAAATTAAACCAAACGAAATTATATCTAATCCAAAATAATAAGA 9300**

**GQ918272.1 AATTCAAAAACTCAATAAAATTAAACCAAATGAAATTATATCTAATCCAAAATAATAAGA 9300**

******************************** *******************************

**GQ918273.1 AATATAATTAAAATAATTTAATGATCTAAAATTAATTATAAATAAAAAAGTAAAAAAAAA 9360**

**mtAD AATATAATTAAAATAATTTAATGATCTAAAATTAATTATAAATAAAAAAGTAAAAAAAAA 9360**

**GQ918272.1 AATATAATTAAAATAATTTAAAGATCTAAAATTAATTATAAATAGAAAAGTAAAAAAAAA 9360**

*********************** ********************** *****************

**GQ918273.1 CAATAAATTTTGAACCGTTCAATAATTACTTTTTAAAAAAGAAATAGGTAATATAAAAAT 9420**

**mtAD CAATAAATTTTGAACCGTTCAATAATTACTTTTTAAAAAAGAAATAGGTAATATAAAAAT 9420**

**GQ918272.1 CAATAAATTTTGAACCGTTCAATAATTACTTTTTAAAAAAGAAATAGGTAGTATAAAAAT 9420**

**************************************************** ***********

**GQ918273.1 TAATATAAAAACAAACTTTAACATTGCAAAATTGAAAATCTTTGAAAATAATCATTACCA 9480**

**mtAD TAATATAAAAACAAACTTTAACATTGCAAAATTGAAAATCTTTGAAAATAATCATTACCA 9480**

**GQ918272.1 TAATATAAAAACAAACTTTAACATTGCAAAATTGAAAATCTTTGAAAATAATCATTACCA 9480**

****************************************************************

**GQ918273.1 TGAGTTCGAATTATTGATACTAAAATTGAAAGACCTAACACTCCTTCACAAACACAAAAA 9540**

**mtAD TGAGTTCGAATTATTGATACTAAAATTGAAAGACCTAACACTCCTTCACAAACACAAAAA 9540**

**GQ918272.1 TGAGTTCGAATTATTGATACTAAAATTGAAAGACCTAACACTCCTTCACAAACACAAAAG 9540**

***************************************************************

**GQ918273.1 GTTAAAAAAAATATTCTAAAATAAAGTTCATAATTTATAAAATTTAAATAAAAAAATAAA 9600**

**mtAD GTTAAAAAAAATATTCTAAAATAAAGTTCATAATTTATAAAATTTAAATAAAAAAATAAA 9600**

**GQ918272.1 GTTAAAAAAAATATTCTAAAATAAAGTTCATAATTTATAAAATTTAAATAAAAAAATAAA 9600**

****************************************************************

**GQ918273.1 AAAATAAATAATCTTAATACTATAAACTCTAATCTTAATAAAGTTGATAATAAATGTTTT 9660**

**mtAD AAAATAAATAATCTTAATACTATAAACTCTAATCTTAATAAAGTTGATAATAAATGTTTT 9660**

**GQ918272.1 AAAATAAATAATCTTAATACTATAAACTCTAATCTTAATAAAGTTGATAATAAATGTTTT 9660**

****************************************************************

**GQ918273.1 CGATTAGATACAAATACTAAACAACCGAAAATAAATATAATTATCGATAAAATAAATAAT 9720**

**mtAD CGATTAGATACAAATACTAAACAACCGAAAATAAATATAATTATCGATAAAATAAATAAT 9720**

**GQ918272.1 CGATTAGATACAAATACTAAACAACCGAAAATAAATATAATTATCGATAAAATAAATAAT 9720**

****************************************************************

**GQ918273.1 AAATATATATTTGCCATTAATTAGTTTAAATAGTTTAACACCAAAACATTAGTCTTGTAA 9780**

**mtAD AAATATATATTTGCCATTAATTAGTTTAAATAGTTTAACACCAAAACATTAGTCTTGTAA 9780**

**GQ918272.1 AAATATATATTTGCCATTAATTAGTTTAAATAGTTTAACACCAAAACATTAGTCTTGTAA 9780**

****************************************************************

**GQ918273.1 ACTAAAATTAAGATATAATTCTTTTTAAACTTCAAGAAAAAAGAAATCTCTTTTTCACTA 9840**

**mtAD ACTAAAATTAAGATATAATTCTTTTTAAACTTCAAGAAAAAAGAAATCTCTTTTTCACTA 9840**

**GQ918272.1 ACTAAAATTAAGATATAATTCTTTTTAAACTTCAAGAAAAAAGAAATCTCTTTTTCACTA 9840**

****************************************************************

**GQ918273.1 ACTCCCAAAGTTAATATTTTAAATAAACTATTTCTTGATATTACAAAATTAATTATTATA 9900**

**mtAD ACTCCCAAAGTTAATATTTTAAATAAACTATTTCTTGATATTACAAAATTAATTATTATA 9900**

**GQ918272.1 ACTCCCAAAGTTAATATTTTAAATAAACTATTTCTTGATATTACAAAATTAATTATTATA 9900**

****************************************************************

**GQ918273.1 ACATTATGTTTAATTATAAGATTTATTTTTATACAAATAAAACATCCTTTATCAATAGGA 9960**

**mtAD ACATTATGTTTAATTATAAGATTTATTTTTATACAAATAAAACATCCTTTATCAATAGGA 9960**

**GQ918272.1 ACATTATGTTTAATTATAAGATTTATTTTTATACAAATAAAACATCCTTTATCAATAGGA 9960**

****************************************************************

**GQ918273.1 TTAATATTATTAATTCAAACTTTTTTAACTTGTTTAATTACAAGAATTTATGTAAAAACA 10020**

**mtAD TTAATATTATTAATTCAAACTTTTTTAACTTGTTTAATTACAAGAATTTATGTAAAAACA 10020**

**GQ918272.1 TTAATATTATTAATTCAAACTTTTTTAACTTGTTTAATTACAAGAATTTATGTAAAAACA 10020**

****************************************************************

**GQ918273.1 TTTTGATTTTCATATGTATTATTTTTAATTTTTTTAGGAGGAATATTAATTTTATTTATT 10080**

**mtAD TTTTGATTTTCATATGTATTATTTTTAATTTTTTTAGGAGGAATATTAATTTTATTTATT 10080**

**GQ918272.1 TTTTGATTTTCATATGTATTATTTTTAATTTTTTTAGGAGGTATATTAATTTTATTTATT 10080**

******************************************* ********************

**GQ918273.1 TATGTAACTTCATTATCTTCAAATGAAATATTTTCAATATCTTTTAGTTTATCAATAATT 10140**

**mtAD TATGTAACTTCATTATCTTCAAATGAAATATTTTCAATATCTTTTAGTTTGTCAATAATT 10140**

**GQ918272.1 TATGTAACTTCATTATCTTCAAATGAAATATTTTCAATATCTTTTAGTTTATCAATAATT 10140**

**************************************************** ***********

**GQ918273.1 AGATTAATAATCTTTTCTTTTTTTACAATTATTTTTTTTATTATAGATAAATCATTAATT 10200**

**mtAD AGATTAATAATCTTTTCTTTTTTTACAATTATTTTTTTTATTATAGATAAATCATTAATT 10200**

**GQ918272.1 AGATTAATAATCTTTTCTTTTTTTACAATTATTTTTTTTATTATAGATAAATCATTAATT 10200**

****************************************************************

**GQ918273.1 GAACAATTTATTATAAATATAGAAATAGAACAATTATCTAATATAAATAATTTAATTAAT 10260**

**mtAD GAACAATTTATTATAAATATAGAAATAGAACAATTATCTAATATAAATAATTTAATTAAT 10260**

**GQ918272.1 GAACAATTTATTATAAATATAGAAATAGAACAATTATCTAATATAAATAATTTAATTAAT 10260**

****************************************************************

**GQ918273.1 GAAAATATTTTGTCTTTAAATAAAATATATAATTTTCCTACTAATTTAATTACTTTATTA 10320**

**mtAD GAAAATATTTTGTCTTTAAATAAAATATATAATTTTCCTACTAATTTAATTACTTTATTA 10320**

**GQ918272.1 GAAAATATTTTGTCTTTAAATAAAATATATAATTTTCCTACTAATTTAATTACTTTATTA 10320**

****************************************************************

**GQ918273.1 TTAATTAATTACTTATTTTTAACTTTATTAGTAACTGTAAAAATTACTAAAAAATTTTAT 10380**

**mtAD TTAATTAATTACTTATTTTTAACTTTATTAGTAACTGTAAAAATTACTAAAAAATTTTAT 10380**

**GQ918272.1 TTAATTAATTACTTATTTTTAACTTTATTAGTAACTGTAAAAATTACTAAAAAATTTTAT 10380**

****************************************************************

**GQ918273.1 GGTCCACTACGACCAATAAATTAATGTTTAAACCAATTCGAAAAAATCACCCCTTAATTA 10440**

**mtAD GGTCCACTACGACCAATAAATTAATGTTTAAACCAATTCGAAAAAATCACCCCTTAATTA 10440**

**GQ918272.1 GGTCCACTACGACCAATAAATTAATGTTTAAACCAATTCGAAAAAATCACCCCTTAATTA 10440**

****************************************************************

**GQ918273.1 GCATTGCTAACAATGCTTTAGTAGATTTACCCGCACCATCAAATATTTCAGCATGATGAA 10500**

**mtAD GCATTGCTAACAATGCTTTAGTAGATTTACCCGCACCATCAAATATTTCAGCATGATGAA 10500**

**GQ918272.1 GCATTGCTAACAATGCTTTAGTAGATTTACCCGCACCATCAAATATTTCAGCATGATGAA 10500**

****************************************************************

**GQ918273.1 ATTTTGGTTCATTATTAGGATTATGCTTAATAATTCAAATTTTAACCGGATTATTCTTAG 10560**

**mtAD ATTTTGGTTCATTATTAGGATTATGCTTAATAATTCAAATTTTAACCGGATTATTCTTAG 10560**

**GQ918272.1 ATTTTGGTTCATTATTAGGATTATGCTTAATAATTCAAATTTTAACCGGATTATTCTTAG 10560**

****************************************************************

**GQ918273.1 CAATACATTATGCTGCTGATATTGAAACAGCATTTAATAGAGTTAATCATATTTGTCGAG 10620**

**mtAD CAATACATTATGCTGCTGATATTGAAACAGCATTTAATAGAGTTAATCATATTTGTCGAG 10620**

**GQ918272.1 CAATACATTATGCTGCTGATATTGAAACAGCATTTAATAGAGTTAATCATATTTGTCGAG 10620**

****************************************************************

**GQ918273.1 ATGTAAATAATGGATGATTCCTACGAATTTGTCACGCAAATGGAGCATCTTTTTTTTTTG 10680**

**mtAD ATGTAAATAATGGATGATTCCTACGAATTTGTCACGCAAATGGAGCATCTTTTTTTTTTG 10680**

**GQ918272.1 ATGTAAATAATGGATGATTCTTACGAATTTGTCACGCAAATGGAGCATCTTTTTTTTTTG 10680**

********************** *****************************************

**GQ918273.1 CATGTTTATTTATCCATGTAGGACGAGGAGTTTATTATGAATCTTATTTATACCATATAA 10740**

**mtAD CATGTTTATTTATCCATGTAGGACGAGGAGTTTATTATGAATCTTATTTATACCATATAA 10740**

**GQ918272.1 CATGTTTATTTATCCATGTAGGACGAGGAGTTTATTATGAATCTTATTTATATCATATAA 10740**

****************************************************** *********

**GQ918273.1 CATGAAATACAGGAGTAATTATTTTATTTTTAACAATAGCAACAGGATTTTTAGGATATG 10800**

**mtAD CATGAAATACAGGAGTAATTATTTTATTTTTAACAATAGCAACAGGATTTTTAGGATATG 10800**

**GQ918272.1 CATGAAATACAGGAGTAATTATTTTATTTTTAACAATAGCGACAGGATTTTTAGGATATG 10800**

****************************************** *********************

**GQ918273.1 TATTACCTTGAGGACAAATATCATTTTGAGGAGCTACTGTAATTACTAATTTATTATCCG 10860**

**mtAD TATTACCTTGAGGACAAATATCTTTCTGAGGAGCTACTGTAATTACTAATTTATTATCAG 10860**

**GQ918272.1 TATTACCTTGAGGACAAATATCTTTCTGAGGAGCTACTGTAATTACTAATTTATTATCAG 10860**

************************ ** ******************************** ***

**GQ918273.1 CTGTACCTTACTTAGGAATAGATCTAGTACAATGAATTTGAGGAGGATTTGCTGTAGATA 10920**

**mtAD CTGTACCTTATTTAGGAATAGATCTAGTACAATGAATTTGAGGAGGATTTGCTGTAGATA 10920**

**GQ918272.1 CTGTACCTTATTTAGGAATAGATCTAGTACAATGAATTTGAGGAGGATTTGCTGTAGATA 10920**

************ ***************************************************

**GQ918273.1 ACGCAACATTAACTCGATTTTTTACTTTTCATTTTATTTTCCCTTTCATTATTCTTGCTT 10980**

**mtAD ACGCAACATTAACTCGATTTTTTACTTTTCATTTTATTTTCCCTTTCATTATTCTTGCTT 10980**

**GQ918272.1 ACGCAACATTAACTCGATTTTTTACTTTTCATTTTATTTTCCCTTTCATTATTCTTGCTT 10980**

****************************************************************

**GQ918273.1 TAATAATAATTCATTTATTATTCTTACACCAAACAGGATCAAATAATCCATTAGGATTAA 11040**

**mtAD TAATAATAATTCATTTACTATTTTTACACCAAACAGGATCAAATAATCCATTAGGATTAA 11040**

**GQ918272.1 TAATAATAATTCATTTATTATTTTTACACCAAACAGGATCAAATAATCCATTAGGATTAA 11040**

******************* **** ***************************************

**GQ918273.1 ATAGAAATGTTGATAAAATTCCTTTTCATCCTTACTTTATCTATAAGGATATTTTTGGAT 11100**

**mtAD ATAGAAATGTTGATAAAATTCCTTTTCATCCTTACTTTATCTATAAGGATATTTTTGGAT 11100**

**GQ918272.1 ATAGAAATGTTGATAAAATTCCTTTTCATCCTTACTTTATCTATAAGGATATTTTTGGAT 11100**

****************************************************************

**GQ918273.1 TTATTGTATTTTTATGAATTTTAGTAGCATTTATTTGAAAATTTAATTATTTATTAATAG 11160**

**mtAD TTATTGTATTTTTATGAATTTTAGTAGCATTTATTTGAAAATTTAATTATTTATTAATAG 11160**

**GQ918272.1 TTATTGTATTTTTATGAATTTTAGTAGCATTTATTTGAAAATTTAATTATTTATTAATAG 11160**

****************************************************************

**GQ918273.1 ATCCAGAAAATTTCATTCCAGCTAATCCTTTAGTAACTCCAGTTCATATTCAACCCGAAT 11220**

**mtAD ATCCAGAAAATTTCATCCCAGCTAATCCTTTAGTAACTCCAGTCCATATTCAACCCGAAT 11220**

**GQ918272.1 ATCCAGAAAATTTCATTCCAGCTAATCCTTTAGTAACTCCAGTCCATATTCAACCCGAAT 11220**

****************** ************************** ******************

**GQ918273.1 GATATTTTTTATTTGCTTATGCAATTTTACGATCAATTCCTAATAAGTTAGGGGGAGTAA 11280**

**mtAD GATATTTTTTATTTGCTTATGCAATTTTACGATCAATTCCTAATAAATTAGGAGGAGTAA 11280**

**GQ918272.1 GATATTTTTTATTTGCTTATGCAATTTTACGATCAATTCCTAATAAATTAGGAGGAGTAA 11280**

************************************************ ***** *********

**GQ918273.1 TTGCATTAGTATTATCAATTGCTATTTTATTAATTTTACCTTTTACTCATGCTAGTAAAT 11340**

**mtAD TTGCATTAGTATTATCAATTGCTATTTTATTAATTTTACCTTTTACTCATGCTAGTAAAT 11340**

**GQ918272.1 TTGCATTAGTATTATCAATTGCTATTTTATTAATTTTACCTTTTACTCATGCTAGTAAAT 11340**

****************************************************************

**GQ918273.1 TCCGAGGTTTACAATTTTATCCATTAAATCAAATTTTATATTGAAATATAGTAATTGTTG 11400**

**mtAD TCCGAGGTTTACAATTTTATCCATTAAATCAAATTTTATATTGAAATATAGTAATTGTTG 11400**

**GQ918272.1 TCCGAGGTTTACAATTTTATCCATTAAATCAAATTTTATATTGAAATATAGTAATTGTTG 11400**

****************************************************************

**GQ918273.1 CTTCATTATTAACATGAATTGGAGCACGACCAGTAGAAGATCCATATATTTTAACAGGAC 11460**

**mtAD CTTCATTATTAACATGAATTGGAGCACGACCAGTAGAAGATCCATATATTTTAACAGGAC 11460**

**GQ918272.1 CTTCATTATTAACATGAATTGGAGCACGACCAGTAGAAGATCCATATATTTTAACAGGAC 11460**

****************************************************************

**GQ918273.1 AAATTCTTACAGTATTATATTTTTCATATTTTATTATTAACCCATTAGTAGCTAAGTATT 11520**

**mtAD AAATTCTTACAGTATTATATTTTTCATATTTTATTATTAACCCATTAGTAGCTAAGTATT 11520**

**GQ918272.1 AAATTCTTACAGTATTATATTTTTCATATTTTATTATTAACCCATTAGTAGCTAAGTATT 11520**

****************************************************************

**GQ918273.1 GAGATAAATTATTAAATTAATTAATAAGCTTTTATAGCATATGTCTTGAAAACATAAGAA 11580**

**mtAD GAGATAAATTATTAAATTAATTAATAAGCTTTTATAGCATATGTCTTGAAAACATAAGAA 11580**

**GQ918272.1 GAGATAAATTATTAAATTAATTAATAAGCTTTTATAGCATATGTCTTGAAAACATAAGAA 11580**

****************************************************************

**GQ918273.1 AGAAGTAAAGTCTTCTATTAATTTATACTAAAAATTATTCATTAAAATAATAAAGAAATT 11640**

**mtAD AGAAGTAAAGTCTTCTATTAATTTATACTAAAAATTATTCATTAAAATAATAAAGAAATT 11640**

**GQ918272.1 AGAAGTAAAGTCTTCTATTAATTTATACTAAAAATTATTCATTAAAATAATAAAGAAATT 11640**

****************************************************************

**GQ918273.1 AAAAAAATTTTAAACCCTACAAAAAAAAATAAATAATTCAAAGAAAGAGGTAAAAAACTT 11700**

**mtAD AAAAAAATTTTAAACCCTACAAAAAAAAATAAATAATTCAAAGAAAGAGGTAAAAAACTT 11700**

**GQ918272.1 AAAAAAATTTTAAACCCTACAAAAAAAAATAAATAATTCAAGGAAAGAGGTAAAAAACTT 11700**

******************************************* ********************

**GQ918273.1 TTTCAAGCTAAATATATTAACTTATCATATCGAAATCGAGGTAAAGTCCCTCGCACTCAA 11760**

**mtAD TTTCAAGCTAAATATATTAACTTATCATATCGAAATCGAGGTAAAGTCCCTCGCACTCAA 11760**

**GQ918272.1 TTTCAAGCTAAGTATATTAACTTATCATATCGAAATCGAGGTAAAGTCCCTCGCACTCAA 11760**

************* **************************************************

**GQ918273.1 ATAAAAATAAAAGAAATAAAAGTTAACTTAAAAAAAAATAATAAACTATAAATATCTCCT 11820**

**mtAD ATAAAAATAAAAGAAATAAAAGTTAACTTAAAAAAAAATAATAAACTATAAATATCTCCT 11820**

**GQ918272.1 ATAAAAATAAAAGAAATAAAAGTTAACTTAAAAAAAAATAATAAACTATAAATATCCCCT 11820**

********************************************************** *****

**GQ918273.1 CCTAAAAAAATCACTACAAATAATATACTTATAAATAAAATTCTTGAATATTCTGCTAAA 11880**

**mtAD CCTAAAAAAATCACTACAAATAATATACTTATAAATAAAATTCTTGAATATTCTGCTAAA 11880**

**GQ918272.1 CCTAAAAAAATCACTACAAATAATATACTTATAAATAAAATTCTTGAATATTCTGCTAAA 11880**

****************************************************************

**GQ918273.1 AAAATTAAAGCAAATCCTCCTCTTCTATATTCAACATTAAACCCAGAAACTAATTCAGAT 11940**

**mtAD AAAATTAAAGCAAATCCTCCTCTTCTATATTCAACATTAAACCCAGAAACTAATTCAGAT 11940**

**GQ918272.1 AAAATTAAAGCAAATCCTCCTCTTCTATATTCAACATTAAACCCAGAAACTAATTCAGAT 11940**

****************************************************************

**GQ918273.1 TCTCCTTCGGCAAAATCAAAAGGAGTTCGATTAGTTTCAGCTAAACAAGAAGCTAATCAA 12000**

**mtAD TCTCCTTCGGCAAAATCAAAGGGAGTTCGATTAGTTTCAGCTAAACAAGAAGCTAATCAA 12000**

**GQ918272.1 TCTCCTTCGGCAAAATCAAAAGGAGTCCGATTAGTTTCAGCTAAACAAGAAGCTAATCAA 12000**

********************** ***** ***********************************

**GQ918273.1 ACTAGCCCTAAAGGAAAACAAAAAAAAATAAACCAAATATAAGACTGAAAATTATAAAAA 12060**

**mtAD ACTAATCCTAAAGGAAAACAAAAAAAAATAAACCAAATATAAGACTGAAAATTATAAAAA 12060**

**GQ918272.1 ACTAATCCTAAAGGAAAACAAAAAAAAATAAACCAAATATAAGACTGAAAATTATAAAAA 12060**

****** ********************************************************

**GQ918273.1 TTTATAAAATTATAATTTCCAATTAAAAAAATAAAACTTAATAAAATTAAAGCTAATCTA 12120**

**mtAD TTTATAAAATTATAATTTCCAATTAAAAAAATAAAACTTAATAAAATTAAAGCTAATCTA 12120**

**GQ918272.1 TTTATAAAATTATAATTTCCAATTAAAAAAATAAAACTTAATAAAATTAAAGCTAATCTA 12120**

****************************************************************

**GQ918273.1 ACTTCATAAGAAATAGTTTGAGCAACCGCTCGTAACCCTCCTAATAAAGCATAATTAGAA 12180**

**mtAD ACTTCATAAGAAATAGTTTGAGCAACCGCTCGTAACCCTCCTAATAAAGCATAATTAGAA 12180**

**GQ918272.1 ACTTCATAAGAAATAGTTTGAGCAACCGCTCGTAATCCTCCTAATAAAGCATAATTAGAG 12180**

************************************* *************************

**GQ918273.1 TTTGAAGATCACCCCGCAATTATTACTGTATAAACTCCTAAACTAGTACAACATAAAAAA 12240**

**mtAD TTTGAAGATCACCCCGCAATTATTACTGTATAAACTCCTAAACTAGTACAACATAAAAAA 12240**

**GQ918272.1 TTTGAAGATCACCCCGCAATTATTACTGTATAAACACCTAAACTAGTACAACATAAAAAA 12240**

************************************* **************************

**GQ918273.1 AACAAAACACCCAAATTAAAAGAGTATAATTTAATTAAATAAGGAATACATATTCAAATC 12300**

**mtAD AACAAAACACCCAAATTAAAAGAGTATAATTTAATTAAATAAGGAATACATATTCAAATC 12300**

**GQ918272.1 AACAAAACACCCAAATTAAAAGAGTATAACTTAATTAAATAAGGAATACATATTCAAATC 12300**

******************************* ********************************

**GQ918273.1 AATAAAGATAAAAACAAAGAAAACACTGGAGAAAAATAATAAGAAATATAATTAGATACT 12360**

**mtAD AATAAAGATAAAAACAAAGAAAACACTGGAGAAAAATAATAAGAAATATAATTAGATACT 12360**

**GQ918272.1 AATAAAGATAAAAACAAAGAAAACACTGGAGAAAAATAATAAGAAATATAATTAGATACT 12360**

****************************************************************

**GQ918273.1 AAAGGATAAGTTTGTTCTTTAGTAAACAATTTTACAGCATCGCTAAAAGGTTGTAATAAT 12420**

**mtAD AAAGGATAAGTTTGTTCTTTAGTAAACAATTTTACAGCATCACTAAAAGGTTGTAATAAT 12420**

**GQ918272.1 AAAGGATAAGTTTGTTCTTTAGTAAACAATTTTACAGCATCACTAAAAGGTTGTAATAAT 12420**

******************************************* ********************

**GQ918273.1 CCGTTAAACCCTACTTTATTTGGTCCTTTACGAATTTGAATATACCCTAATACCTTACGT 12480**

**mtAD CCGTTAAACCCTACTTTATTTGGTCCTTTACGAATTTGAATATACCCTAATACCTTACGT 12480**

**GQ918272.1 CCGTTAAACCCTACTTTATTTGGTCCTTTACGGATTTGAATATATCCTAATACCTTACGT 12480**

********************************** *********** *****************

**GQ918273.1 TCTAATAAAGTTAAAAATGCTACTCCTACTATTACACAAATTACTAATAATAATCTTCCA 12540**

**mtAD TCTAATAAAGTTAAAAATGCTACTCCTACTATTACACAAATTACTAATAATAATCTTCCA 12540**

**GQ918272.1 TCTAATAAAGTTAAAAATGCTACTCCTACTATTACACAAATTACTAATAATAATCTTCCA 12540**

****************************************************************

**GQ918273.1 ATTAAAGGTAAAATTAAATCATATATAAACAATACTATTTATAATTAAAAATTATATTTA 12600**

**mtAD ATTAAAGGTAAAATTAAATCATATATAAACAATACTATTTATAATTAAAAATTATATTTA 12600**

**GQ918272.1 ATTAAAGGTAAAATTAAATCATATATAAACAATACTATTTATAATTAAAAATTATATTTA 12600**

****************************************************************

**GQ918273.1 TAAATTCTAAATTTATTGCACTAATCTGCCAAAATAGTAAATAAAATTATTAATTTTCAA 12660**

**mtAD TAAATTCTAAATTTATTGCACTAATCTGCCAAAATAGTAAATAAAATTATTAATTTTCAA 12660**

**GQ918272.1 TAAATTCTAAATTTATTGCACTAATCTGCCAAAATAGTAAATAAAATTATTAATTTTCAA 12660**

****************************************************************

**GQ918273.1 ATAAATTAAAATTATATTTTTTATATTAGGTCCTTTCGTACTACAATATAATAATTAATT 12720**

**mtAD ATAAATTAAAATTATATTTTTTATATTAGGTCCTTTCGTACTACAATATAATAATTAATT 12720**

**GQ918272.1 ATAAATTAAAATTATATTTTTTATATTAGGTCCTTTCGTACTACAATATAATAATTAATT 12720**

****************************************************************

**GQ918273.1 AAGGATAGAAACCAACCTGGCTTACGCCGGTTTGAACTCAGATCATGTAAGAATTCAAAG 12780**

**mtAD AAGGATAGAAACCAACCTGGCTTACGCCGGTTTGAACTCAGATCATGTAAGAATTCAAAG 12780**

**GQ918272.1 AAGGATAGAAACCAACCTGGCTTACGCCGGTTTGAACTCAGATCATGTAAGAATTCAAAG 12780**

****************************************************************

**GQ918273.1 GTCGAACAGACCTAAACTTTAAACTTCTACACCTAAAAATAACTCTTAATCCAACATCGA 12840**

**mtAD GTCGAACAGACCTAAACTTTAAACTTCTACACCTAAAAATAACTCTTAATCCAACATCGA 12840**

**GQ918272.1 GTCGAACAGACCTAAACTTTAAACTTCTACACCTAAAAATAATTCTTAATCCAACATCGA 12840**

******************************************** *******************

**GQ918273.1 GGTCGCAATCTTTTTTATCGATATGAACTCTCTAAAAAAATTACGCTGTTATCCCTAAGG 12900**

**mtAD GGTCGCAATCTTTTTTATCGATATGAACTCTCTAAAAAAATTACGCTGTTATCCCTAAGG 12900**

**GQ918272.1 GGTCGCAATCTTTTTTATCGATATGAACTCTCTAAAAAAATTACGCTGTTATCCCTAAGG 12900**

****************************************************************

**GQ918273.1 TAACTTAATTTTTTAATCCATAATAAAGGATCTAAAATTCATATATCAATGTCAATAATA 12960**

**mtAD TAACTTAATTTTTTAATCCATAATAAAGGATCTAAAATTCATATATCAATGTCAATAATA 12960**

**GQ918272.1 TAACTTAATTTTTTAATCCATAATAAAGGATCTAAAATTCATATATCAATGTCAATAATA 12960**

****************************************************************

**GQ918273.1 AATAAAAGTTTATTAAATTTTAATACCACCCCAGTAAAATTTTATCAAAAATATAAATTT 13020**

**mtAD AATAAAAGTTTATTAAATTTTAATACCACCCCAGTAAAATTTTATCAAAAATATAAATTT 13020**

**GQ918272.1 AATAAAAGTTTATTAAATTTTAATACCACCCCAGTAAAATTTTATCAAAAATATAAATTT 13020**

****************************************************************

**GQ918273.1 TAAAATTCTTTATAATTTATAATTTATAAAAATAAAGATCTATAGGGTCTTCTCGTCCTT 13080**

**mtAD TAAAATTCTTTATAATTTATAATTTATAAAAATAAAGATCTATAGGGTCTTCTCGTCCTT 13080**

**GQ918272.1 TAAAATTCTTTATAATTTATAATTTATAAAAATAAAGATCTATAGGGTCTTCTCGTCCTT 13080**

****************************************************************

**GQ918273.1 TAATTACATTTTAACTTTTTAATTAAAAAATAAAGTTCTATAAAAATTTAAAAAAAACAG 13140**

**mtAD TAATTACATTTTAACTTTTTAATTAAAAAATAAAGTTCTATAAAAATTTAAAAAAAACAG 13140**

**GQ918272.1 TAATTACATTTTAACTTTTTAATTAAAAAATAAAATTCTATAAAAATTTAAAAAAAACAG 13140**

************************************ ***************************

**GQ918273.1 TATATATCTCATTCAACCATTCATACCAGCCTTCAATTAAAAGACTATTGATTATGCTAC 13200**

**mtAD TATATATCTCATTCAACCATTCATACCAGCCTTCAATTAAAAGACTATTGATTATGCTAC 13200**

**GQ918272.1 TATATATCTCATTCAACCATTCATACCAGCCTTCAATTAAAAGACTATTGATTATGCTAC 13200**

****************************************************************

**GQ918273.1 CTTCGCACGGTCAAAATACCGCGGCCCTTTAAATTTCAGTGGGCAGGCTAGACTTTATAT 13260**

**mtAD CTTCGCACGGTCAAAATACCGCGGCCCTTTAAATTTCAGTGGGCAGGCTAGACTTTATAT 13260**

**GQ918272.1 CTTCGCACGGTCAAAATACCGCGGCCCTTTAAATTTCAGTGGGCAGGCTAGACTTTATAT 13260**

****************************************************************

**GQ918273.1 ATAAATCAAAAAGACATGTTTTTGTTAAACAGGTGAACATATTTAATTTGCCGAATTCTT 13320**

**mtAD ATAAATCAAAAAGACATGTTTTTGTTAAACAGGTGAACATATTTAATTTGCCGAATTCTT 13320**

**GQ918272.1 ATAAATCAAAAAGACATGTTTTTGTTAAACAGGTGAACATATTTAATTTGCCGAATTCTT 13320**

****************************************************************

**GQ918273.1 CATTTAAACCTATCAAATTAACTACATTATATAAATTTATATACTAATTTTATCATAATT 13380**

**mtAD CATTTAAACCTATCAAATTAACTACATTATATAAATTTATATACTAATTTTATCATAATT 13380**

**GQ918272.1 CATTTAAACCTATCAAATTAACTACATTATATAAATTTATATACTAATTTTATCATAATT 13380**

****************************************************************

**GQ918273.1 TATAATTTTAAATAATTAAAAATTATATTTTAATAAAAAATTTAATTTAAAAATAAATAA 13440**

**mtAD TATAATTTTAAATAATTAAAAATTATATTTTAATAAAAAATTTAATTTAAAAATAAATAA 13440**

**GQ918272.1 TATAATTTTAAATAATTAAAAATTATATTTTAATAAAAAATTTAATTTAAAAATAAATAA 13440**

****************************************************************

**GQ918273.1 TTTTAAATAAAAAATAAATTATAACAAATTTATTAATAATAGCTATTTTTAAGCTTATAT 13500**

**mtAD TTTTAAATAAAAAATAAGTTATAACAAATTTATTAATAATAGCTATTTTTAAGCTTATAT 13500**

**GQ918272.1 TTTTAAATAAAAAATAAATTATAACAAATTTATTAATAATAGCTATTTTTAAGCTTATAT 13500**

******************* ********************************************

**GQ918273.1 TTATTTTTTAATTATTAAAAATATAAAAATTTATTTTAAAGCTTATCCCTTAAAATATTA 13560**

**mtAD TTATTTTTTAATTATTAAAAATATAAAAATTTATTTTAAAGCTTATCCCTTAAAATATTA 13560**

**GQ918272.1 TTATTTTTTAATTATTAAAAATATAAAAATTTATTTAAAAGCTTATCCCTTAAAATATTA 13560**

************************************** *************************

**GQ918273.1 TTTTATTCATTTATTAAATTAAAAAAAATTAAATTAATAAAATAAATAAACTAAATTAAA 13620**

**mtAD TCTTATTCATTTATTAAATTAAAAAAAATTAAATTAATAAAATAAATAAACTAAATTAAA 13620**

**GQ918272.1 TTTTATTCATTTATTAAATTAAAAAAAATTAAATTAATAAAATAAATAAACTAAATTAAA 13620**

*** ************************************************************

**GQ918273.1 TTTATTTCTTAAAAAACTAGATATATTTTAAAACGATTAACATTTCATTTCTAATCATAT 13680**

**mtAD TTTATTTCTTAAAAAACTAGATATATTTTAAACCGATTAACATTTCATTTCTAATCATAT 13680**

**GQ918272.1 TTTATTTCTTAAAAAACTAGATATATTTTAAAACGATTAACATTTCATTTCTAATCATAT 13680**

********************************** *****************************

**GQ918273.1 ATTAAAAATAGTTATTCCACAATAACTTTTATATACAATTAAATCTTTAAAATTCGAGAA 13740**

**mtAD ATTAAAAATAGTTATTCCACAATAACTTTTATATACAATTAAATCTTTAAAATTCGAGAA 13740**

**GQ918272.1 ATTAAAAATAGTTATTCCACAATAACTTTTATATACAATTAAATCTTTAAAATTCGAGAA 13740**

****************************************************************

**GQ918273.1 AAATTATTATAATAATTAATTATTTAATAAACCCTGATACACAAGGTACAATAAATAAAA 13800**

**mtAD AAATTATTATAATAATTAATTATTTAATAAACCCTGATACACAAGGTACAATAAATAAAA 13800**

**GQ918272.1 AAATTATTATAATAATTAATTATTTAATAAACCCTGATACACAAGGTACAATAAATAAAA 13800**

****************************************************************

**GQ918273.1 TTTTCTTCTAAAATAAAAATTTTTCAAATTATTTCAATATTCTTTTACAATACTAATACA 13860**

**mtAD TTTTCTTCTAAAATAAAAATTTTTCAAATTATTTCAATATTCTTTTACAATACTAATACA 13860**

**GQ918272.1 TTTTCTTCTAAAATAAAAATTTTTCAAATTATTTCAATATTCTTTTACAATACTAATACA 13860**

****************************************************************

**GQ918273.1 CTATAATTAAAATTATTATTTCATTATAAATTACTTAAACTAAAAATATTAAAATTATTT 13920**

**mtAD CTATAATTAAAATTATTATTTCATTATAAATTACTTAAACTAAAAATATTAAAATTATTT 13920**

**GQ918272.1 CTATAATTAAAATTATTATTTCATTATAAATTACTTAAACTAAAAATATTAAAATTATTT 13920**

****************************************************************

**GQ918273.1 TTATTAATAATTAACTAAAAATAAAATAAATTAATAAATAAATATTATCAATTTAAATTG 13980**

**mtAD TTATTAATAATTAACTAAAAATAAAATAAATTAATAAATAAATATTATCAATTTAAATTG 13980**

**GQ918272.1 TTATTAATAATTAACTAAAAATAAAATAAATTAATAAATAAATATTATCAATTTAAATTG 13980**

****************************************************************

**GQ918273.1 AATTGCACAAATTTCTTTTCAATGTAAATGAAATACTTTGCAAATTAAGCTTTAAATTGT 14040**

**mtAD AATTGCACAAATTTCTTTTCAATGTAAATGAAATGCTTTACTAATTAAGCTTTAAATTGT 14040**

**GQ918272.1 AATTGCACAAATTTCTTTTCAATGTAAATGAAATGCTTTACTAATTAAGCTTTAAATTGT 14040**

************************************ **** * ********************

**GQ918273.1 CATTCTAGATACACTTTCCAGTACATCTACTATGTTACGACTTATCTCATTTTAAAAATG 14100**

**mtAD CATTCTAGATACACTTTCCAGTACATCTACTATGTTACGACTTATCTCATTTTAAAAATG 14100**

**GQ918272.1 CATTCTAGATACACTTTCCAGTACATCTACTATGTTACGACTTATCTCATTTTAAAAATG 14100**

****************************************************************

**GQ918273.1 AGAGCGACGGGCGATGTGTGCATGTTTTAGAGCTTTAATCATATAAATAATCTATTTTAT 14160**

**mtAD AGAGCGACGGGCGATGTGTGCATGTTTTAGAGCTTTAATCATATAAATAATCTATTTTAT 14160**

**GQ918272.1 AGAGCGACGGGCGATGTGTGCATGTTTTAGAGCTTTAATCATATAAATAATCTATTTTAT 14160**

****************************************************************

**GQ918273.1 ATTACTATTAAATCCACCTTCATATCTTTATTTCAAAAAATATTCCGTATAAATAATTTT 14220**

**mtAD ATTACTATTAAATCCACCTTCATATCTTTATTTCAAAAAATATTCCGTATAAATAATTTT 14220**

**GQ918272.1 ATTACTATTAAATCCACCTTCATATCTTTATTTCAAAAAATATTCCGTATAAATAATTTT 14220**

****************************************************************

**GQ918273.1 ATTGTAATCCATTTCTACTTAACTATAAACTGCACCTTGACCTGACATTTTATTTAATAA 14280**

**mtAD ATTGTAATCCATTTCTACTTAACTATAAACTGCACCTTGACCTGACATTTTATTTAATAA 14280**

**GQ918272.1 ATTGTAATCCATTTCTACTTAACTATAAACTGCACCTTGACCTGACATTTTATTTAATAA 14280**

****************************************************************

**GQ918273.1 AATATTTAGAAAATTATTAATCTTATAATATATTCTGATGACGGCGATATACAAATTGAA 14340**

**mtAD AATATTTAGAAAATTATTAATCTTATAATATATTCTGATGACGGCGATATACAAATTGAA 14340**

**GQ918272.1 AATATTTAGAAAATTATTAATCTTATAATATATTCTGATGACGGCGATATACAAATTGAA 14340**

****************************************************************

**GQ918273.1 AACAAAATTAAGTTAGGTCCAACGTGGATTATCAATAACAGAACAGATTCCTCTAAATAG 14400**

**mtAD AACAAAGTTAAGTTAGGTCCAACGTGGATTATCAATAACAGAACAGATTCCTCTAAATAG 14400**

**GQ918272.1 AACAAAGTTAAGTTAGGTCCAACGTGGATTATCAATAACAGAACAGATTCCTCTAAATAG 14400**

******** *******************************************************

**GQ918273.1 ACTAAAACACCGCCAAATTCTTTAAATTTTAAGAATATAACTAATACTACTTTAGCATTT 14460**

**mtAD ACTAAAACACCGCCAAATTCTTTAAATTTTAAGAATATAACTAATACTACTTTAGCATTT 14460**

**GQ918272.1 ACTAAAACACCGCCAAATTCTTTAAATTTTAAGAATATAACTAATACTACTTTAGCATTT 14460**

****************************************************************

**GQ918273.1 TAATTATTTAATTTTAATAATAGGGTATCTAATCCTAGTTTATTATAAAATTTTTATAGC 14520**

**mtAD TAATTATTTAATTTTAATAATAGGGTATCTAATCCTAGTTTATTATAAAATTTTTATAGC 14520**

**GQ918272.1 TAATTATTTAATTTTAATAATAGGGTATCTAATCCTAGTTTATTATAAAATTTTTATAGC 14520**

****************************************************************

**GQ918273.1 TTAAATTAATTAAAA-TTAATATTAAATAAATTTAAAAATTTCACCTAATAGATGTATAA 14579**

**mtAD TTAAATTAATTAAAAATTAATATTAAATAAATTTAAAAATTTCACCTAATAAATTTATAA 14580**

**GQ918272.1 TTAAATTAATTAAAAGTTAATATTAAATAAATTTAAAAATTTCACCTAATAAATTTATAA 14580**

***************** *********************************** ** *******

**GQ918273.1 ATATATTAAATAAGTGTAATGGAACTAATAGATAAAAATTTTTATTTGCATCATTTGTAT 14639**

**mtAD ATATATTAAATAAATATAATTTAACTAATAC-TAAAAATTTTTATTTGCATCATTTGTAT 14639**

**GQ918272.1 ATATATTAAATAAATATAATTTAACTAATAC-TAAAAATTTTTATTTGCATCATTTGTAT 14639**

*************** * **** ******** ******************************

**GQ918273.1 AACCGCAGTAGCTGGCACAAATTTTACCAATACTATATATTATTACTAATTCAAAATTTC 14699**

**mtAD AACCGCAGTAGCTGGCACAAATTTTACCAATACTATATATTATTACTAATTCAAAATTTC 14699**

**GQ918272.1 AACCGCAGTAGCTGGCACAAATTTTACCAATACTATATATTATTACTAATTCAAAATTTC 14699**

****************************************************************

**GQ918273.1 TTTTATAATTAATATTAATTACTGCGAATAATTTATAATTATTTATTTTTAAAATTAATA 14759**

**mtAD TTTTATAATTAATACTAATTACTGCGAATAATTTATAATTATTTATTTTTAAAATTAATA 14759**

**GQ918272.1 TTTTATAATTAATATTGATTACTGCGAATAATTTACAATTATTTATTTTTAAAATTAATA 14759**

**************** * ****************** **************************

**GQ918273.1 ATAATTCATACAAAAATTTACATGTAAAATAAAATATAAATAAAATATTTAACTAGAATA 14819**

**mtAD ATAATTCATACAAAAATTTACATGTAAAATAAAATATAAATAAAATATTTAACTAGAATA 14819**

**GQ918272.1 ATAATTCATACAAAAATTTACATGTAAAATAAAATATAAATAAAATATTTAACTAGAATA 14819**

****************************************************************

**GQ918273.1 AAATAATATTAATACATTTAAATTTATAATAATAAATTATAATAATATTTTTTATTTATC 14879**

**mtAD AAATAATATTAATACATTTAAATTTATAATAATAAATTATAATAATATTTTTTATTTATT 14879**

**GQ918272.1 AAATAATATTAATACATTTAAATTTATAATAATAAATTATAATAATATTTTTTATTTATC 14879**

***************************************************************

**GQ918273.1 ATTAAAATAATTAATAAAATTTTATTAATTTATTAATATTAAATAATTAAATTAAATAAA 14939**

**mtAD ATTAAAATAATTATTAAAATTTTATTAATTTATTAATATTAAATAATTAAATTAAATAAA 14939**

**GQ918272.1 ATTAAAATAATTATTAAAATTTTATTAATTTATTAATATTAAATAATTAAATTAAATAAA 14939**

*************** ************************************************

**GQ918273.1 TAATTTTAGTACATTTCTCCCC-ATAAACCCCCTATTTTTTTTTTTTTTTTTATTATTTA 14998**

**mtAD TAATTTTAGTACATTTCTCCCCTATAAACCCCCTATTTTTTTTTTTTTTTTTATTATTTA 14999**

**GQ918272.1 TAATTTTAGTACATTTCTTCCC-ATAAACCCCCTATTTTTTTTTTTTTTTTTATTATTTA 14998**

******************** *** ***************************************

**GQ918273.1 ATTTATAAAAATTATTATTATTATATTATTATATTAAATTAATTAATTTAATTAATTATT 15058**

**mtAD ATTTATAAAAATTATTATTATTATATTATTATATTAAATTAATTAATTTAATTAATTATT 15059**

**GQ918272.1 ATTTATAAAAATTATTATTATTACATTATTATATTAAATTAATTAATTTAATTAATTATT 15058**

************************* **************************************

**GQ918273.1 TTTAATTAATTAAATTATATTATTTATTTTATATAATAATATAAAATAGTATATATATAT 15118**

**mtAD TTTAATTAATTAAATTATATTATTTATTTTATATAATAATATAAAATAGTATATATATAT 15119**

**GQ918272.1 TTTAATTAATTAAATTATATTATTTATTTTATATAATAATATAAAATAGTATATATATAT 15118**

****************************************************************

**GQ918273.1 ATATAAATAT--TTAATATAAATATAAATATATATATATTTAAATTTATATATTAATATA 15176**

**mtAD ATATATAAATA-TTAATATAAATATAAATATATATATATTTAAATTTATATATTAATATA 15178**

**GQ918272.1 ATATATAAATATTTAATATAAATATAAATATA----TATTTAAATTTATATATTAATATA 15174**

******* * ** ******************** **************************

**GQ918273.1 TATTTAATATAAATAATTTATATATATATATATATTTTTTTC-CTTTAAATATAAGACTA 15235**

**mtAD TATTTAATATAAATAAGT-ATATATATATATATATTTTTTTC-CTTTAAATATAAGACTA 15236**

**GQ918272.1 TATTTAATATAAATAATTTATATATATATATATATTTTTTTTTCTTTAAATATAAGACTA 15234**

****************** * ********************** *******************

**GQ918273.1 TTTGGTTTATAAATATATACCCATTTTATATAAATATATTATATGTATAAATATTTATAT 15295**

**mtAD TTTGGTTTATAAATATACACCCATTTTATATAAATATATTATATGTATAAATATTTATAT 15296**

**GQ918272.1 TTTGGTTTATAAATATACACCCATTTTATATAAATATATTATATGTATAAATATTTATAT 15294**

******************* ********************************************

**GQ918273.1 TAATATAAATTATTTATATAGTTAAAAGAACTGAGTTCTAAAAAATAAATTTTTCATTCC 15355**

**mtAD TAATATAAATTATTTATATAGTTAAAAGAACTGAGTTCTAAAAAATAAATTTTTCATTCC 15356**

**GQ918272.1 TAATATAAATTATTTATATAGTTAAAAGAACTGAGTTCTAAAAAATAAAATTTTCATTCC 15354**

*************************************************** ************

**GQ918273.1 GTTATTTTTAATATTAATATAAATAAAA-TA- 15385**

**mtAD GTTATTTTTAATATTAATATAAATAAAA-TAA 15387**

**GQ918272.1 GTTATTTTTAATATTAATATAAATAAAAATAA 15386**

********************************

**Figure** **S-C1. Anopheles darlingi mitochondrial genome alignment.** GQ918273.1 - *Anopheles darlingi* isolate Southern mitochondrion, complete genome (From Manaus, Amazonas State, Brazil)**.**

GQ918272.1 - *Anopheles darlingi* isolate Northern mitochondrion, complete genome (From Central Cayo District of Belize).

mtAD - *Anopheles darlingi* mitochondrion, complete genome assembled in this study (From Coari, Amazonas State, Brazil).
