# Supplementary material for: The Genome of Anopheles darlingi, the main neotropical malaria vector
Source: Nucleic Acids Res. 2013 Jun 12;41(15):7387–400. doi: 10.1093/nar/gkt484 (PMC3753621; doi:10.1093/nar/gkt484)
Supplement: Supplementary Data [file supp_gkt484_nar-00257-h-2013-File006_updated.zip › S-D.docx]

**S-D A physical map of *Anopheles darlingi* chromosomes**

*Anopheles darlingi* possesses a karyotype of 2n=6 chromosomes, as do most Anophelinae species. A detailed *An. darlingi* cytogenetic map [23] and FISH (Fluorescence in situ Hybridization) techniques were used to map rDNA [24], Hsp70 genes [25] and two actin genes [26] to unique sites on chromosomal arms (S-D). According to the obtained data, the *An. darlingi* chromosomal arm 2L is homologous to arm 3L in *An. gambiae*, 2L in *An. stephensi*, 3L in *An. funestus* and 3R in *An. albimanus* [27,28]. Therefore, whole-arm translocations are common evolutionary events among species of the subgenus *Cellia* (*An. gambiae*, *An. stephensi*, *An. funestus*) [28] and the subgenus *Nyssorhynchus* (*An. albimanus*, *An. darlingi*). These data provide an initial basis for establishing chromosomal homologies among major malaria vectors.


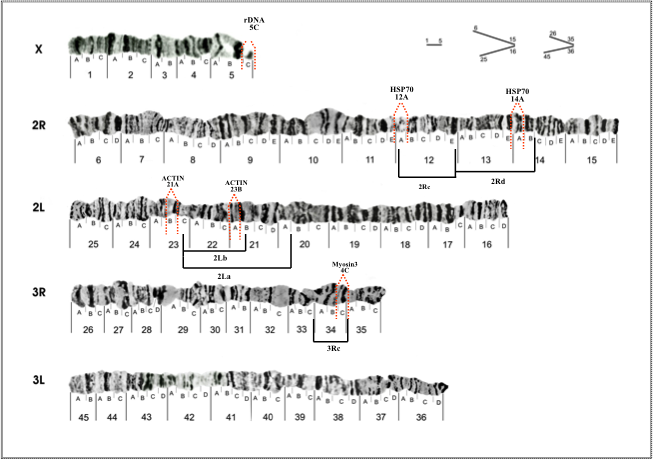


**Figure S-D1**. **Locations of rDNA, HSP70, Actins and Myosin probes marks on *An. darlingi* polytene chromosomes** **Photomap** [23-26].
