# Supplementary material for: The Genome of Anopheles darlingi, the main neotropical malaria vector
Source: Nucleic Acids Res. 2013 Jun 12;41(15):7387–400. doi: 10.1093/nar/gkt484 (PMC3753621; doi:10.1093/nar/gkt484)
Supplement: Supplementary Data [file supp_gkt484_nar-00257-h-2013-File006_updated.zip › S-K.doc]

**S- K. *Anopheles darlingi* immunity related genes**

**Table S-K1. Immune-related genes identified in the *An. darlingi* genome and their closest homologs from *An. gambiae***. Genes involved in the Toll and the IMD pathways as well as antimicrobial peptide genes and Thioesterproteins (TEPs) were identified. Amongst the PGRPs, those predicted to encode for type 2 amidases, which degrade peptidoglycan, are marked with an asterix.

| **Gene name (product)** | ***An. gambiae* gene ID** | ***An. darlingi* gene ID** |
| --- | --- | --- |
| Spz1 | AGAP000346 | AD03608, 03336 |
| Spz3 | AGAP008360 | AD04669 |
| Spz6 | AGAP005126 | AD08912 |
| Toll Receptors | AGAP006974(Toll9), AGAP006974 (Toll9), AGAP01l187 (Toll10), AGAP011186 (Toll11), AGAP012326 (Toll7), AGAP001004 (Toll1A), AGAP010669 (Toll1B), AGAP010636 (Toll5B), AGAP000999 (Toll5A) | AD00052, AD08087, AD02906, AD00106, AD10060, AD01495, AD01498 |
| MyD88 | AGAP005252 | AD10657 |
| Tube | AGAP003062 | AD02968 |
| Pelle | AGAP002966 | AD03846 |
| TRAF6 | AGAP003004 | AD09927 |
| Cactus (IκB like) | AGAP007938 | AD06079 |
| NFkB (REL1) | AGAP009515 | AD01392 |
| PGRPs | AGAP005552 (LD), AGAP005204 (LA2), AGAP005203 (LC2), AGAP005203 (LC3)  AGAP000536 (S1), AGAP006342, AGAP006343 (S2/S3), AGAP001212 (LB) | AD00043, AD03232, AD03233, AD03246, AD08274, AD07851*, AD02673*, AD09269* |
| IMD | AGAP004959 | AD11085 |
| Caspar/FAS-associated factor1 | AGAP006473 | AD05975 |
| FADD | AGAP007173 | AD08782 |
| DREDD | AGAP011693 | AD03947 |
| DIAP2 | AGAP007294 | AD02192 |
| TAK1 | AGAP013516 | AD09872 |
| TAB2 | AGAP002953 | AD00593 |
| IKKgama | AGAP005933 | AD05684 |
| IKKbeta | AGAP009166 | AD05408 |
| REL2 | AGAP006747 | AD02697 |
| Defensins | AGAP011294 (DEF1), AGAP004632 (DEF2), AGAP007199 (DEF3), AGAP005416 (DEF4) | AD01122 |
| Cecropins | AGAP000692, AGAP000693, AGAP000694, AGAP006722 | AD03801, AD03802, AD03803 |
| TEPs | AGAP008654 (TEP12), AGAP008407 (TEP13), AGAP008364 (TEP15), AGAP008366 (TEP2), AGAP010812 (TEP4), AGAP008368 (TEP14)  AGAP010815, 010816, 010814, 010813, 010830, 010819, 010818 (TEP1/3/5/6/9/10/11) | AD10630, AD07871, AD09512, AD09518, AD08670, AD09520  AD10169, 01651, 03391  AD10172 |

**A**

**B**

**C**

**Figure S-K1. Phylogeny of Spatzle 3A (A), MyD88 (B) and SPZ gene family (C).** The reconstructions were done by the Neighbor-Joining method of sequences from *An. darling*, *An. gambiae*, *Ae. aegypti*, *Cu. quinquefasciatus* and *D. melanogaster*. The accession numbers or gene IDs for the sequences are listed below.

| ***An. gambiae SPZ*** | ***An. darlingi SPZ*** | ***Ae. aegypti*** | ***D. melanogaster*** |
| --- | --- | --- | --- |
| AGAP 000346 (SPZ1) | AD03608 | XP_001663628.1 | NP_001138116.1 |
|  | AD03336 |  |  |
| AGAP 006483 (SPZ2) |  | XP_001662706.1 | NP_001014576.1 |
| AGAP 008360 (SPZ3) | AD04669 | XP_001653322.1 | NP_609160.2 |
| AGAP 007866 (SPZ4) | - | ABM68624.1 | NP_609504 |
| AGAP 007177 (SPZ5) | - | XP_001654338 | NP_647753.1 |
| AGAP 005126 (SPZ6) | AD08912 | XP_001655918.1 | NP_611961.1 |
